# Expanding the chemical diversity of an endophytic fungus *Bulgaria inquinans*, an ascomycete associated with mistletoe, through an OSMAC approach†

**DOI:** 10.1039/c9ra03678d

**Published:** 2019-08-13

**Authors:** Ni P. Ariantari, Georgios Daletos, Attila Mándi, Tibor Kurtán, Werner E. G. Müller, Wenhan Lin, Elena Ancheeva, Peter Proksch

**Affiliations:** Institute of Pharmaceutical Biology and Biotechnology, Heinrich Heine University Düsseldorf Universitätsstrasse 1 40225 Düsseldorf Germany Elena.Ancheeva@uni-duesseldorf.de proksch@uni-duesseldorf.de +49 211 81 11923 +49 211 81 14175 +49 211 81 14163; Department of Pharmacy, Faculty of Mathematic and Natural Sciences, Udayana University 80361 Bali Indonesia; Department of Organic Chemistry, University of Debrecen P.O.B. 400 4002 Debrecen Hungary; Institute for Physiological Chemistry, University Medical Center of the Johannes Gutenberg University Mainz Duesbergweg 6 55128 Mainz Germany; State Key Laboratory of Natural and Biomimetic Drugs, Peking University 100191 Beijing China

## Abstract

An endophytic fungus *Bulgaria inquinans* (isolate MSp3-1), isolated from mistletoe (*Viscum album*), was subjected to fermentation on solid Czapek medium. Chromatographic workup of the crude EtOAc extract yielded five new natural products (1–5). Subsequent application of the “One Strain, MAny Compounds” (OSMAC) strategy on this strain by the addition of a mixture of salts (MgSO_4_, NaNO_3_ and NaCl) to solid Czapek medium induced the accumulation of nine additional new secondary metabolites (6–13, 16), with most of them (8, 10–12) not detectable in cultures lacking the salt mixture. The structures of the new compounds were established on the basis of the 1D/2D NMR and HRESIMS data. The TDDFT-ECD method was applied to determine the absolute configurations of the new compounds 1, 4 and 6 as well as of the previously reported bulgarialactone B (14), for which the absolute configuration was unknown so far. The modified Mosher's method was performed to assign the absolute configurations of 12 and 13. TDDFT-ECD analysis also allowed determining the absolute configuration of (+)-epicocconone, which had an enantiomeric absolute configuration in the tricyclic moiety compared to that of bulgarialactone B (14). All the isolated metabolites were evaluated for their cytotoxic activity. Compound 2 was found to possess strong cytotoxic activity against the murine lymphoma cell line L5178Y with an IC_50_ value of 1.8 μM, while the remaining metabolites were shown to be inactive.

## Abstract 2

OSMAC approach on endophytic *Bulgaria inquinans* by addition of a mixture of salts (MgSO_4_, NaNO_3_ and NaCl) to solid Czapek medium induced the accumulation of new secondary metabolites.

## Graphical abstract

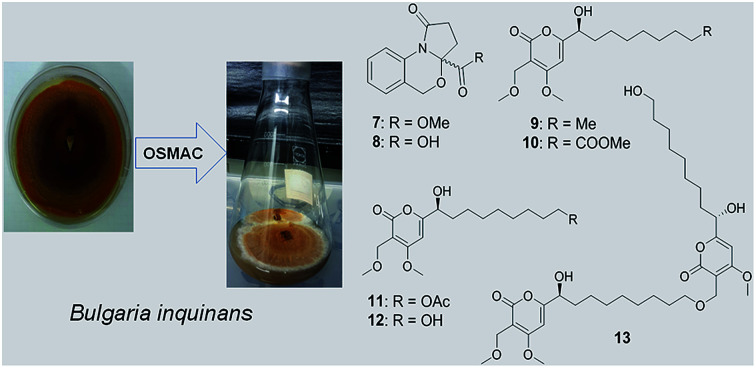

## Introduction

Fungi are well-known producers of novel drug leads, as exemplified by the fascinating discovery of beta-lactam antibiotics, cyclosporin A, caspofungin, lovastatin and fingolimod.^1–3^ In particular, endophytic fungi, which live asymptomatically within plant tissues, have been recognized for their capability to produce therapeutically interesting natural products.^4^ Remarkable examples include the antimycotic natural products cryptocandin^5^ and cryptocin,^6^ the insecticidal compound nodulisporic acid A,^7^ the mitochondrial toxin phomoxanthone A^8,9^ as well as the immunosuppressant diterpene pyrones subglutinols A and B,^10^ among others. Interestingly, isolation of the important anticancer agents, paclitaxel from *Taxomyces andreanae*, an endophyte of *Taxus brevifolia*,^11^ and camptothecin produced by *Entrophospora infrequens*, a fungus associated with *Nothapodytes foetida*,^12^ as well as the identification of lycopodine-type alkaloids recently detected in an UV-irradiated strain of *Paraboeremia*, a fungal endophyte of *Lycopodium serratum* var. *longipetiolatum*,^13^ highlights the special importance of endophytes as a reservoir of metabolites previously known only from the host plant.^14,15^

Gene clusters involved in the biosynthesis of fungal secondary metabolites often remain silent under standard laboratory culture conditions, leading to a frequent rediscovery of known metabolites.^16,17^ To overcome this problem, several strategies for enhancing the biosynthetic potential of fungi can be applied. One of them is the OSMAC (One Strain, MAny Compounds) approach,^18^ a powerful experimental method used to enhance the chemical diversity of microorganisms using the selective modification of the fermentation parameters, such as the media type and composition, the physical parameters (pH value, temperature, aeration conditions), the addition of enzyme inducers/inhibitors and chemical elicitors.^16–19^ Even a difference in water quality in preparing the culture media, such as exchanging tap water for distilled water, was reported to influence the pattern of the main metabolites of *Paraphaeosphaeria quadriseptata*, due to the presence of traces of metal ions (Cu^2+^, Cd^2+^ and Cr^3+^) in tap water.^20^ Successful application of the OSMAC approach on the fungal endophyte, *Dothideomycete* sp. CRI7 by changing the medium type (PDB *vs.* Czapek malt medium) as well as by using different nutrient sources (potato for PDB medium and malt extract for Czapek malt medium) resulted in distinct secondary metabolite production of this fungus.^21,22^ Interestingly, a series of studies have reported the isolation of halogenated natural products from fungal cultures grown on media to which different halide salts had been added, thus highlighting the fungal capability to utilize different halogen sources when present in the media.^23–26^ In addition, supplementing media with trace elements, *e*.*g*. the addition of CuCl_2_ to cultures of *Pestalotiopsis* sp. Z233 or the addition of ZnSO_4_ to cultures of *Aspergillus clavatus*, induced the production of new sesquiterpenes possessing tyrosinase inhibitory activity,^27^ and the production of a new metabolite, clavatustide C,^28^ respectively.

As part of our ongoing studies aimed at influencing the biosynthetic capacity of endophytic fungi utilizing the OSMAC approach, we investigated the metabolic profiles of *Bulgaria inquinans* (isolate MSp3-1), an ascomycete fungus isolated from sprouts of common mistletoe (*Viscum album*). Previous chemical investigations of this fungus resulted in the isolation of azaphilone pigments, namely bulgarialactones A–D,^29,30^ of which bulgarialactones A and B exhibited antimicrobial, cytotoxic and nematocidal activities.^29^ Moreover, quinones containing a benzofluoranthenequinone nucleus, bulgarhodin and bulgarein^31^ as well as cytotoxic anthraquinone dimers, bulgareones A and B,^32^ were isolated from this fungus.

In the present study, we report the isolation and structure elucidation of 14 new natural products, including five new natural products (1–5) derived from *B. inquinans* cultured on solid Czapek medium and nine new compounds (6–13, 16) isolated from this fungus when grown on solid Czapek medium with the addition of a salt mixture (MgSO_4_, NaNO_3_ and NaCl). Furthermore, determination of the absolute configurations of the new compounds and of the known derivative, bulgarialactone B (14), is described herein. The cytotoxicity assay results of the isolated natural products are briefly discussed.

## Results and discussion

**Fig. 1:**
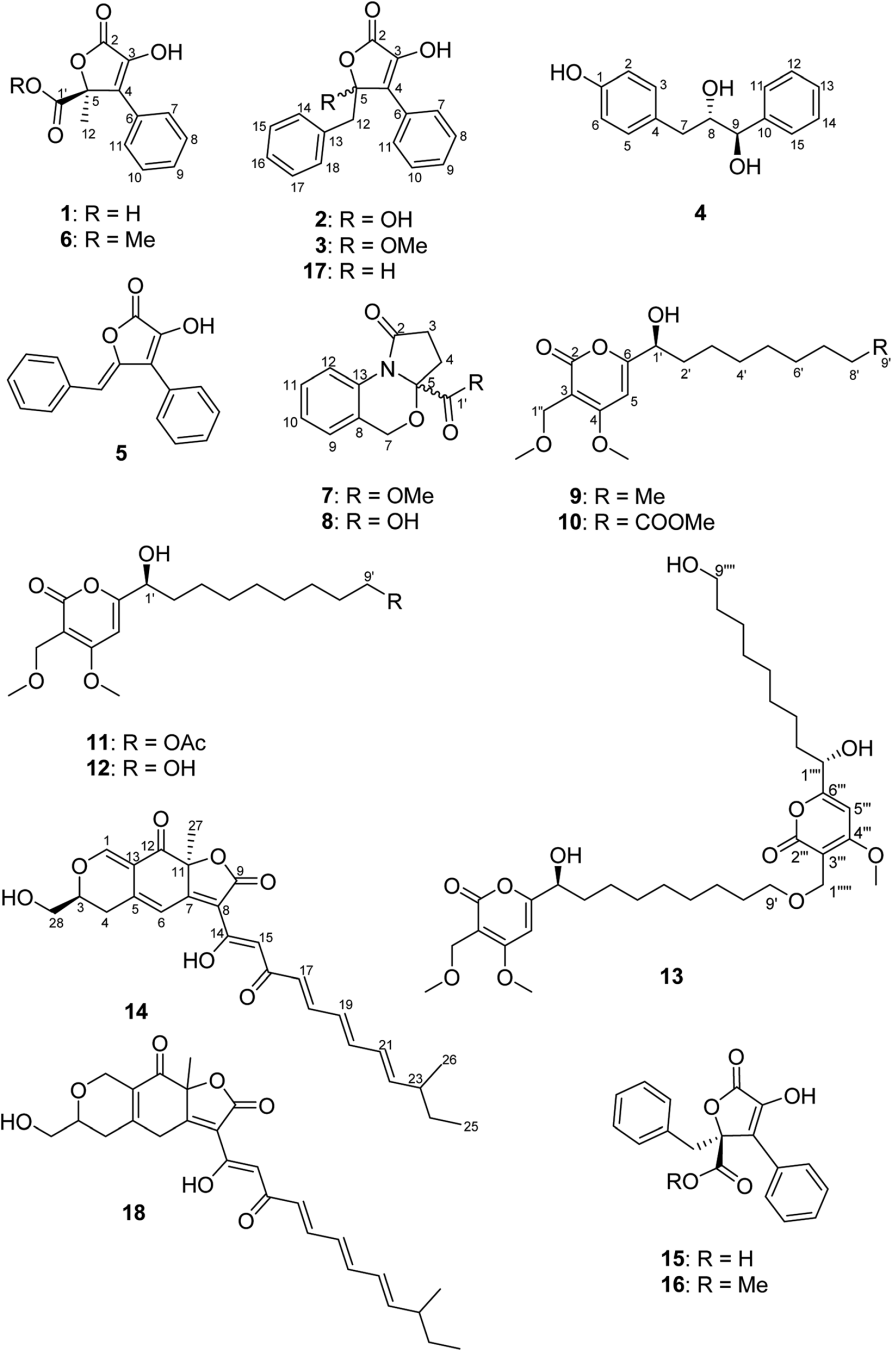
Structures of compounds 1–18 isolated from *B. inquinans*.

Chemical investigation of *B. inquinans* grown on solid Czapek medium resulted in the isolation of five new natural products, namely butyrolactones (1–3, 5) and a new metabolite bearing a diol moiety (4) together with known compounds, such as the azaphilone pigment bulgarialactone B (14)^29^ as well as phenylbutyrolactone IIa (15)^33,34^ and xenofuranone B (17)^35^ (Fig. 1). After this fermentation, we studied the chemical profiles of the fungus when grown in the presence of different salts that had been added to solid Czapek medium. *B*. *inquinans* was cultured on solid Czapek medium following the addition of either NaCl, NaBr, NaI, NaNO_3_, or (NH_4_)_2_SO_4_ (3.5 g of each) or following the addition of salt mixtures: (a) MgSO_4_·7H_2_O, NaNO_3_ and NaCl (2.5 g each), (b) FeSO_4_·7H_2_O, NaNO_3_ and NaCl (2.5 g each) or (c) ZnSO_4_·7H_2_O, NaNO_3_ and NaCl (2.5 g each), as described in the Experimental section. *B. inquinans* failed to grow on media containing either (NH_4_)_2_SO_4_, or mixtures (b) and (c). No changes in the chromatographic profiles were observed following the addition of either NaCl, NaBr, NaI or NaNO_3_ to solid Czapek medium when compared to chromatograms of the fungus grown on the medium without salts. However, the presence of a mixture of MgSO_4_, NaNO_3_ and NaCl in the medium resulted in a significant change in the metabolite profile of *B. inquinans*, as indicated by HPLC-DAD analysis (Fig. S1, ESI†). Chromatographic workup of this fungal extract led to the isolation of nine new secondary metabolites (6–13, 16), including a butyrolactone derivative 6, two unusual 1,3-oxazine containing natural products (7 and 8), five new α-pyrones (9–13) and (−)-(*S*)-flavipesin B (16), together with the known compound bulgarialactone D (18)^30^ (Fig. 1). Compounds 9, 13 and 18 were subsequently also detected (albeit in minor amounts) in HPLC chromatograms of the fungus following cultivation on solid Czapek medium without the addition of salts. The OSMAC approach nevertheless enhanced their production, enabling isolation and structural characterization of these compounds. Moreover, the accumulation of compounds 8 and 10–12 was only induced in the presence of the salt mixture, whereas these latter compounds were not detected in fungal cultures lacking salts.

**Fig. 2:**
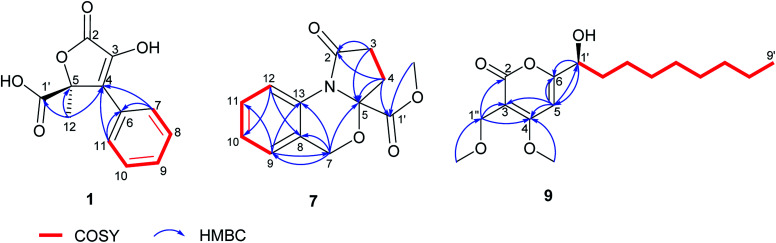
COSY and selected HMBC correlations of 1, 7 and 9.

Compound 1 was obtained as a yellow solid. Its UV spectrum displayed a maximum absorption at 288 nm, which was characteristic for butyrolactone-type metabolites.^36^ The HRESIMS spectrum exhibited a prominent pseudomolecular ion peak at *m*/*z* 253.0600 [M + H]^+^, attributed to the molecular formula C_12_H_10_O_5_, indicating 8 degrees of unsaturation. The ^1^H NMR data of 1 (Table 1) revealed signals of one methyl at *δ*_H_ 1.74 (H_3_-12) and five aromatic protons resonating at *δ*_H_ 7.74 (H-7/H-11), 7.41 (H-8/H-10) and 7.34 (H-9), implying the presence of a mono-substituted benzene ring. Moreover, the ^13^C NMR data of 1 (Table 1) displayed signals of a carbonyl carbon at *δ*_C_ 170.3 (C-2), two sp^2^ carbons at *δ*_C_ 140.6 (C-3) and 131.2 (C-4), and one oxygenated sp^3^ carbon at *δ*_C_ 85.2 (C-5), attributed to a conjugated five-membered lactone ring. The HMBC correlations observed from H-7/H-11 to C-4 and from H_3_-12 to C-4, C-5 and C-1′ (Fig. 2) provided the connections of the aromatic ring and of the methyl group to the butyrolactone ring as well as the attachment of the carboxylic acid group to C-5.

**Table 1:** ^1^H and ^13^C NMR data (MeOH-*d*_4_)^a^ for compounds 1–3 and 5–6

Position	1		2		3		5		6	
	*δ*_C,_ type	*δ*_H_ (*J* in Hz)	*δ*_C,_ type	*δ*_H_ (*J* in Hz)	*δ*_C,_ type	*δ*_H_ (*J* in Hz)	*δ*_C,_ type	*δ*_H_ (*J* in Hz)	*δ*_C,_ type	*δ*_H_ (*J* in Hz)
2	170.3, C		168.9, C		168.1, C		166.6, C		169.9, C	
3	140.6, C		141.4, C		143.0, C		141.4, C		140.8, C	
4	131.2, C		127.3, C		123.9, C		126.5, C		130.6, C	
5	85.2, C		107.4, C		110.4, C		148.1, C		84.9, C	
6	131.4, C		132.3, C		131.8, C		130.5, C		131.2, C	
7/11	128.7, CH	7.74, br d (7.5)	129.4, CH	8.00, br d (7.5)	128.8, CH	7.94, br d (7.4)	130.4, CH	7.57, br d (7.5)	128.6, CH	7.66, br d (7.3)
8/10	129.6, CH	7.41, br t (7.5)	129.5, CH	7.46, br t (7.5)	129.8, CH	7.48, br d (7.4)	129.8, CH	7.51, br t (7.5)	129.8, CH	7.42, br t (7.3)
9	129.7, CH	7.34, tt (7.5, 1.2)	129.4, CH	7.37, tt (7.5, 1.6)	129.8, CH	7.39, tt (7.4, 1.1)	128.9, CH	7.45, tt (7.5, 1.3)	129.8, CH	7.35, tt (7.3, 1.2)
12	22.1, CH_3_	1.76, s	44.9, CH_2_	3.39, d (13.8); 3.34, d (13.8)	44.6, CH_2_	3.37, d (13.8); 3.34, d (13.8)	109.6, CH	5.97, s	22.2, CH_3_	1.79, s
13			135.7, C		135.2, C		135.3, C			
14/18			131.4, CH	6.85, br d (6.9)	131.5, CH	6.85, br d (6.9)	130.9, CH	7.68, br d (7.4)		
15/17			128.8, CH	7.10, br t (6.9)	128.8, CH	7.10, br t (6.9)	129.6, CH	7.35, br t (7.4)		
16			127.9, CH	7.12, tt (6.9, 1.6)	128.0, CH	7.12, tt (6.9, 1.5)	128.8, CH	7.25, tt (7.4, 1.1)		
5-OMe					50.9, CH_3_	3.24, s				
1′	172.4, C								171.4, C	
1′-OMe									53.9, CH_3_	3.76, s

a Recorded at 600 MHz (^1^H) and 150 MHz (^13^C).

**Fig. 3:**
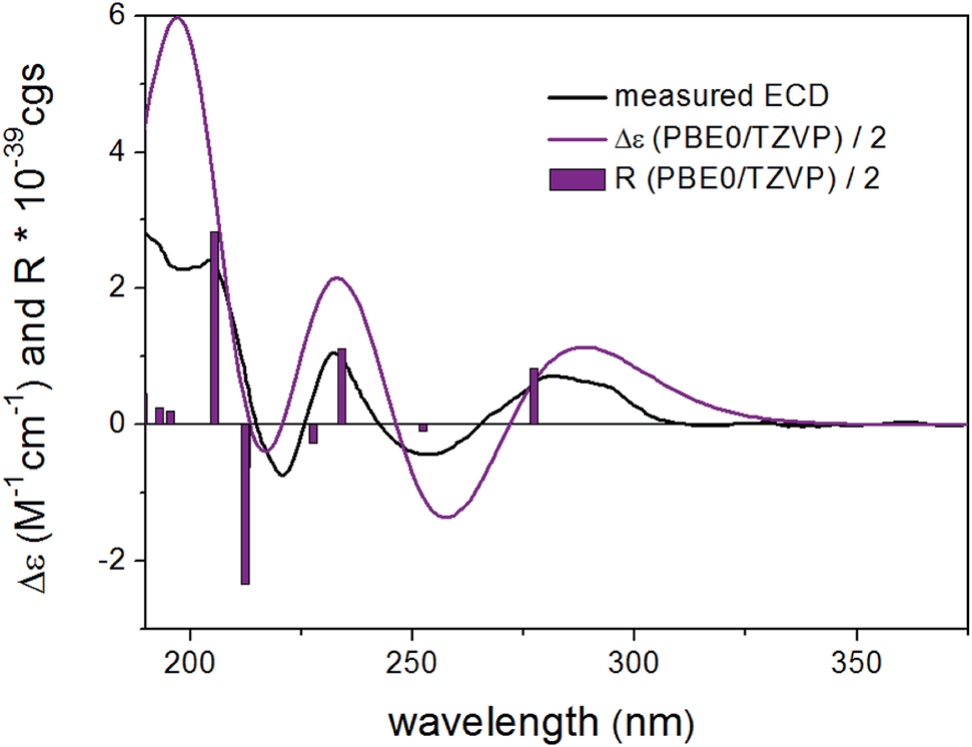
Experimental ECD spectrum (black) of 1 in MeCN compared with the Boltzmann-weighted PBE0/TZVP PCM/MeCN ECD spectrum (purple) of (*S*)-1 computed for the eight low-energy CAM-B3LYP/TZVP PCM/MeCN conformers. The bars represent the rotational strength of the lowest-energy conformer.

In order to elucidate the absolute configuration of 1, the TDDFT-ECD protocol was performed on the arbitrarily chosen (*S*) stereoisomer. B3LYP/6-31+G(d,p) and CAM-B3LYP/TZVP PCM/MeCN reoptimization of the initial 13 MMF conformers resulted in three and eight low-energy conformers over a 1% Boltzmann-population, respectively. ECD spectra computed at various levels for both sets of conformers effectively reproduced the experimental ECD spectrum of 1 (Fig. 3), allowing the elucidation of the absolute configuration as (*S*). Accordingly, the structure of 1 was established as a new natural product, for which the name bulgariline A is proposed (Fig. 1).

The molecular formulae of 2 and 3 were assigned as C_17_H_14_O_4_ and C_18_H_16_O_4_, respectively, based on their prominent pseudomolecular ion peaks in the HRESIMS spectra. The ^1^H and ^13^C NMR data of 2 (Table 1) were almost identical to those of phenylbutyrolactone IIa (15), except for the replacement of the carboxylic acid functionality with a hydroxyl group, as indicated by the absence of a carbonyl signal in the ^13^C NMR spectrum, as well as a 28 amu difference in the molecular weight of 2 compared with that of phenylbutyrolactone IIa (15). The NMR data of 3 (Table 1) were in good agreement with those of 2, apart from the presence of a methoxy signal at *δ*_H_ 3.24 (*δ*_C_ 50.9). Accordingly, a methoxy substituent was assigned to C-5 instead of a hydroxyl group as in 2, based on the evident HMBC correlation from 5-OMe to C-5. Thus, 2 and 3 were elucidated as new butyrolactone derivatives and were named as bulgarilines B and C, respectively. The zero value of their specific rotations indicated that 2 and 3 were obtained as racemic mixtures.

The molecular formula of 4 was determined as C_15_H_16_O_3_ based on its HRESIMS data, implying 8 degrees of unsaturation. Investigation of its ^1^H NMR data (Table 2) revealed typical signals of phenyl and *p*-hydroxy phenyl moieties in the structure of 4. In addition, consecutive COSY correlations were observed between a set of methylene protons resonating at *δ*_H_ 2.51/2.84 (H_2_-7) and two *O*-methine protons at *δ*_H_ 3.87 and 4.53 (H-8 and H-9), which permitted the assignment of a 1,2-propanediol moiety. The HMBC correlations from H_2_-7 to C-3, C-4, C-5, C-8 and C-9 and in turn from H-9 to C-10, C-11 and C-15 established the connectivity of the partial structures. The relative configuration of 4 was deduced to be *erythro*, based on the large coupling constant value between the two vicinal methines at positions 8 and 9 (5.8 Hz), while a smaller value (*J* = 2.5 Hz) is suggested for *threo*, as previously reported for related vicinal diols.^37,38^ Hence, the structure of 4 was elucidated and the trivial name bulgariol is suggested for this compound.

**Table 2:** ^1^H and ^13^C NMR data (MeOH-*d*_4_) for compound 4

Position	4^a^	
	*δ*_C,_ type	*δ*_H_ (*J* in Hz)
1	156.6, C	
2/6	115.9, CH	6.68, br d (8.5)
3/5	131.4, CH	7.02, br d (8.5)
4	131.6, C	
7	38.9, CH_2_	2.51, dd (14.2, 9.2)
		2.84, dd (14.2, 3.1)
8	77.8, CH	3.87, ddd (9.2, 5.8, 3.1)
9	78.2, CH	4.53, d (5.8)
10	143.5, C	
11/15	128.4, CH	7.41, br d (7.0)
12/14	129.0, CH	7.33, br t (7.0)
13	128.3, CH	7.26, tt (7.0, 1.5)

a Recorded at 600 MHz (^1^H) and 150 MHz (^13^C).

**Fig. 4:**
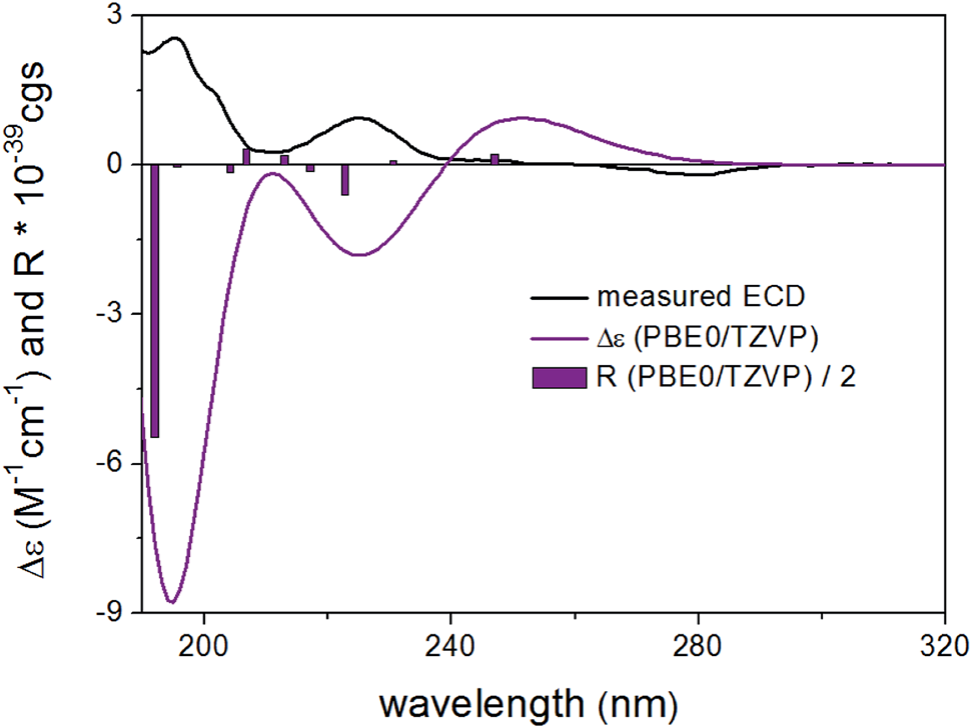
Experimental ECD spectrum (black) of 4 in MeCN compared with the Boltzmann-weighted PBE0/TZVP PCM/MeCN ECD spectrum (purple) of (8*R*,9*S*)-4 computed for the 22 low-energy CAM-B3LYP/TZVP PCM/MeCN conformers. The bars represent the rotational strength of the lowest-energy conformer.

DFT reoptimization of the initial 41 MMFF conformers of (8*R*,9*S*)-4 resulted in 15 and 22 low-energy conformers over a 1% Boltzmann-population. Despite the flexibility of the molecule and the substantially different ECD spectra of the individual low-energy conformers, the Boltzmann-averaged ECD spectra computed at various levels for both sets of conformers gave moderate to good mirror-image agreement with the experimental ECD spectrum (Fig. 4). Furthermore, the sign of the highest-wavelength ECD transition was the same for all the conformers over a 1.2% Boltzmann-population, allowing elucidation of the absolute configuration as (8*S*,9*R*).

Compound 5 had the molecular formula C_17_H_12_O_3_, requiring 12 degrees of unsaturation. The ^1^H and ^13^C NMR data of 5 (Table 1) were similar to those of 2 and 3. However, an additional olefinic signal appeared at *δ*_H_ 5.97 (H-12), which provided HMBC correlations to C-4, C-5, C-13, C-14/C-18, along with a long-range correlation to C-2. Thus, the position of the double bond was deduced at Δ^5(12)^, contributing to one additional degree of unsaturation of 5 compared to 2 and 3. The geometry of the exocyclic double bond C-5/C-12 in 5 was determined based on the NOESY spectrum. The NOE correlations observed between H-12 with H-7/H-11 implied the close proximity of H-12 to the aromatic ring, thus confirming the *Z* configuration of the double bond.^39,40^ Compound 5, (5*Z*)-3-hydroxy-4-phenyl-5-(phenylmethylene)-2(5*H*)-furanone, was reported previously only as a synthetic product.^41^ In the present study, we report 5 for the first time as a natural product and provide its complete NMR data (Table 1).

The molecular ion of 6 was 14 amu larger than that of 1, as indicated by the HRESIMS spectrum, corresponding to the molecular formula C_13_H_12_O_5_. The ^1^H and ^13^C NMR data of 6 (Table 1) were in perfect agreement with those of 1, except for the additional methoxy group signal at *δ*_H_ 3.76/*δ*_C_ 53.9, which showed the respective HMBC correlation to C-1′, hinting at a substitution of the carboxylic acid group with a carbomethoxy attached to C-5 in the structure of 6. Finally, 6 was elucidated as a methyl ester of bulgariline A to which the trivial name bulgariline D is given.

The absolute configuration of 6 was concluded to be the same as that of 1 on the basis of biogenetic considerations, similar positive values of their specific rotation, [*α*]^20^_D_ +16 for 1 and [*α*]^25^_D_ +26 for 6, as well as the identical ECD spectra of 1 and 6. Furthermore, TDDFT-ECD calculations performed on the arbitrarily chosen (*R*) enantiomer gave mirror-image agreement (Fig. S106, ESI†), verifying the (*S*) absolute configuration of 6. It is interesting to compare the ECD spectra of 1 and 6 with those of the related compounds chaetobutenolide C, WF-3681 and WF-3681 methyl ester described by some of us in 2017 and where the carboxylic acid/ester group is attached through two carbons to the central unit.^36^ The positive transition at *ca.* 260–280 nm, which is stronger in two recent cases, and the negative one around 220 nm, belonging to the homochiral derivatives, becomes more complex in 1 and 6, but they are still in accordance with the two characteristic bands. Moreover, 6 was proven to be a true natural product, by incubating 1.0 mg of 1 in 1.0 mL MeOH containing 0.1% formic acid at room temperature for 1 week, which did not result in methylation, as indicated by HPLC analysis.

Nitrogen-containing metabolites (7 and 8) were obtained as yellow solids. The HRESIMS spectra exhibited prominent pseudomolecular ion peaks at *m*/*z* 248.0919 [M + H]^+^ and *m*/*z* 234.0760 [M + H]^+^, consistent with the molecular formula C_13_H_13_NO_4_ and C_12_H_11_NO_4_, respectively, both corresponding to 8 degrees of unsaturation. Inspection of the ^1^H NMR spectrum of 7 (Table 3) revealed the resonances of four aromatic protons, three sets of methylenes and one methoxy group. The COSY correlations of the aromatic protons from H-9 until H-12 suggested a 1,2-disubstituted aromatic ring for 7, as corroborated by HMBC correlations from H-9 to C-11 and C-13 as well as from H-12 to C-8 and C-10. The last spin system observed in the COSY spectrum between *δ*_H_ 2.60/2.67 (H_2_-3) and 2.30/2.54 (H_2_-4) afforded HMBC correlations from H_2_-3 to C-2 and C-5 and from H_2_-4 to C-5 and C-1′, respectively. Additionally, the HMBC correlation from 1′-OMe to C-1′ established the substructure of an oxoproline residue bearing a 5-carbomethoxy group (Fig. 2). Further HMBC correlations from the methylene at *δ*_H_ 4.96/5.10 (H_2_-7) to C-5, C-8, C-9 and C-13 established the linkage between these two substructures, which accounted for the last degree of unsaturation in 7. The ^1^H and ^13^C NMR data of 8 were in a good agreement with those of 7, apart from the absence of an OMe signal, which is in accordance with the 14 amu difference in the molecular weight of 8 compared to 7, thus indicating a carboxylic acid group attached at C-5, instead of a carbomethoxy function. Accordingly, 7 and 8 possess unusual heterocyclic structures containing a 1,3-oxazine nucleus, which rarely occurs in natural products. A few examples of natural 1,3-oxazines include the antimycobacterial oxazinin A, isolated from the *Eurotiomycetes* strain 110162,^42^ as well as salinazinones A and B from the bacterial strain *Streptomyces* sp. KMF-004.^43^ The latter two compounds have been also reported from the marine-derived bacterium *Streptomyces spinoverrucosus* as spinoxazines A and B.^44^ However, compared to the aforementioned natural products, the structures of 7 and 8 lack a keto function in the 1,3-oxazine core, which is mostly attributed to synthetic compounds.^45,46^ The trivial names bulgarixines A (7) and B (8) were assigned to these compounds. Considering the possibility that 7 might arise as an artifact of its non-methoxylated analogue (8) during the isolation process, an experiment was carried out by incubating 0.5 mg of 8 in 0.5 mL MeOH containing 0.1% formic acid for 1 week at room temperature. HPLC analysis showed that no methylated product was present in the chromatogram, thus indicating that 7 is a natural product. The baseline ECD curves of both 7 and 8 indicated that both of them were isolated as a racemate.

**Table 3:** ^1^H and ^13^C NMR data (MeOH-*d*_4_) for compounds 7 and 8

Position	7^a^		8^a^	
	*δ*_C,_ type	*δ*_H_ (*J* in Hz)	*δ*_C,_ type^b^	*δ*_H_ (*J* in Hz)
2	174.3, C		174.3, C	
3	30.6, CH_2_	2.60, ddd (17.2, 10.1, 2.3)	30.5, CH_2_	2.59, ddd (17.2, 10.1, 2.2)
		2.67, dt (17.2, 9.6)		2.70, dt (17.2, 9.7)
4	31.4, CH_2_	2.30, dt (13.5, 10.1)	31.3, CH_2_	2.28, dt (13.5, 10.1)
		2.54, ddd (13.5, 9.6, 2.3)		2.54, ddd (13.5, 9.7, 2.2)
5	92.6, C		92.6, C	
7	66.1, CH_2_	4.96, d (15.8);	65.7, CH_2_	4.93, d (15.7)
		5.10, d (15.8)		5.19, d (15.7)
8	124.4, C		124.6, C	
9	125.5, CH	7.10, br d (8.0)	125.2, CH	7.10, br d (8.0)
10	125.9, CH	7.14, td (8.0, 1.2)	125.3, CH	7.13, td (8.0, 1.2)
11	128.5, CH	7.27, br t (8.0)	128.0, CH	7.26, br t (8.0)
12	120.4, CH	8.27, br d (8.0)	120.3, CH	8.29, br d (8.0)
13	134.0, C		133.8, C	
1′	171.4, C		172.6, C	
1′-OMe	53.7, CH_3_	3.73, s		

a Recorded at 600 MHz (^1^H) and 150 MHz (^13^C).

b Chemical shifts extracted from HSQC and HMBC spectra.

Compound 9 was afforded as a brown solid. Its molecular formula was determined as C_17_H_28_O_5_ on the basis of its HRESIMS spectrum, requiring 4 degrees of unsaturation. Its UV spectrum displayed a maximum absorption at around 299 nm, suggesting a pyrone nucleus.^47,48^ Detailed analysis of the ^1^H and ^13^C NMR data of 9 (Table 4) indicated signals of an isolated olefinic proton at *δ*_H_ 6.65 (H-5), an oxygenated methine at *δ*_H_ 4.40 (H-1′) and an isolated methylene at *δ*_H_ 4.31 (H_2_-1′′), in addition to seven aliphatic methylenes observed at *δ*_H_ 1.31–1.82 (H_2_-2′–H_2_-8′), two methoxy groups at *δ*_H_ 4.00 and 3.32, one methyl group at *δ*_H_ 0.90 (H_3_-9′), along with signals for a carbonyl at *δ*_C_ 166.7 (C-2) and sp^2^ carbons at *δ*_C_ 101.8 (C-3), 171.7 (C-4) and 171.4 (C-5). COSY correlations observed from H-1′ to H_3_-9′ allowed the assignment of a 1-nonanol side-chain (Fig. 2). The HMBC correlations from H-5 to C-3, C-4 and C-6, as well as from H_2_-1′′ to C-2, C-3 and C-4, confirmed the presence of a conjugated α-pyrone nucleus in the structure of 9. Additionally, the position of two methoxy groups (4-OMe and 1′′-OMe) as substituents of the α-pyrone ring was deduced based on their respective HMBC correlations. Finally, the presence of HMBC correlations from H-5 to C-1′ and in turn from H-1′ to C-5 and C-6, indicated the connection of the 1-nonanol side-chain with the α-pyrone nucleus at C-6 (Fig. 2). The structure of 9 is closely related to dothideopyrone B, which was isolated from the endophyte *Dothideomycete* sp. LRUB20,^49^ and with dothideopyrone F, a recently reported α-pyrone from the endolichenic fungus *Dothideomycetes* sp. EL003334.^47^ However, 9 features two extra methylene protons in the saturated aliphatic chain in comparison with dothideopyrone B,^49^ and an additional OMe at C-1′′ in comparison with dothideopyrone F.^47^ Therefore, compound 9 was elucidated as a new α-pyrone derivative, for which the trivial name bulgariapyrone A is suggested.

**Table 4:** ^1^H and ^13^C NMR data (MeOH-*d*_4_) for compounds 9–13

Position	9^a^		10^a^		11^a^		12^a^		13^b^^,^^d^	
	*δ*_C,_ type^c^	*δ*_H_ (*J* in Hz)	*δ*_C,_ type	*δ*_H_ (*J* in Hz)	*δ*_C,_ type^c^	*δ*_H_ (*J* in Hz)	*δ*_C,_ type	*δ*_H_ (*J* in Hz)	*δ*_C,_ type	*δ*_H_ (*J* in Hz)
2	166.7, C		166.8, C		166.7, C		166.8, C		166.7, C	
3	101.8, C		101.8, C		101.8, C		101.8, C		101.9, C	
4	171.7, C		171.7, C		171.7, C		171.7, C		171.7, C	
5	94.4, CH	6.65, s	94.4, CH	6.65, s	94.4, CH	6.65, s	94.4, CH	6.65, s	94.5, CH	6.65, br s
6	171.4, C		171.3, C		171.3, C		171.3, C		171.3, C	
1′	71.5, CH	4.40, dd (7.9, 4.5)	71.5, CH	4.40, m	71.5, CH	4.40, dd (7.9, 4.5)	71.5, CH	4.40, dd (8.0, 4.5)	71.5, CH	4.40, br dd (7.9, 4.4)
2′	36.3, CH_2_	1.68, m; 1.82, m	36.2, CH_2_	1.68, m; 1.83, m	36.2, CH_2_	1.69, m; 1.82, m	36.2, CH_2_	1.68, m; 1.82, m	36.3, CH_2_	1.68, m; 1.82, m
3′	26.2, CH_2_	1.42, m	26.1, CH_2_	1.43, m	26.1, CH_2_	1.44, m	26.1, CH_2_	1.44, m	26.1, CH_2_	1.44, m; 1.41 m
4′	30.4,^e^ CH_2_	1.27–1.37, ov^f^	30.2,^e^ CH_2_	1.31–1.38, ov	30.2,^e^ CH_2_	1.31–1.39, ov	30.4,^e^ CH_2_	1.31–1.39, ov	30.4,^e^ CH_2_	1.30–1.37, ov
5′	30.5,^e^ CH_2_	1.27–1.37, ov	30.1,^e^ CH_2_	1.31–1.38, ov	30.3,^e^ CH_2_	1.31–1.39, ov	30.5,^e^ CH_2_	1.31–1.39, ov	30.5,^e^ CH_2_	1.30–1.37, ov
6′	30.6,^e^ CH_2_	1.27–1.37, ov	30.2,^e^ CH_2_	1.31–1.38, ov	30.4,^e^ CH_2_	1.31–1.39, ov	30.6,^e^ CH_2_	1.31–1.39, ov	30.6,^e^ CH_2_	1.30–1.37, ov
7′	33.0, CH_2_	1.27–1.37, ov	26.0, CH_2_	1.61, p (7.4)	27.0, CH_2_	1.36, ov	26.9, CH_2_	1.34, ov	27.1, CH_2_	1.34, ov
8′	23.7, CH_2_	1.31, ov	34.8, CH_2_	2.31, t (7.4)	29.7, CH_2_	1.62, p (6.7)	33.6, CH_2_	1.52, p (6.7)	30.6, CH_2_	1.54, m
9′	14.4, CH_3_	0.90, t (7.0)	176.0, C		65.7, CH_2_	4.05, t (6.7)	63.0, CH_2_	3.53, t (6.7)	71.6, CH_2_	3.46, t (6.6)
11′					173.1, C					
12′					20.8, CH_3_	2.02, s				
1′′	64.1, CH_2_	4.31, s	64.1, CH_2_	4.30, s	64.1, CH_2_	4.31, s	64.1, CH_2_	4.30, s	64.1, CH_2_	4.30, s
4-OMe	57.7, CH_3_	4.00, s	57.7, CH_3_	4.00, s	57.7, CH_3_	4.00, s	57.7, CH_3_	4.00, s	57.7, CH_3_	4.00, s
9′-OMe			51.9, CH_3_	3.65, s						
1′′-OMe	58.3, CH_3_	3.32, s	58.3, CH_3_	3.32, s	58.3, CH_3_	3.32, s	58.3, CH_3_	3.32, s	58.3, CH_3_	3.32, s

a Recorded at 600 MHz (^1^H) and 150 MHz (^13^C).

b Recorded at 600 MHz (^1^H) and 125 MHz (^13^C).

c Chemical shifts extracted from the HSQC and HMBC spectra.

d Signals for another monomeric unit are identical, except for *δ*_C_ 166.8 (C, C-2′′′), 102.3 (C, C-3′′′), 171.5 (C, C-4′′′), 94.4 (CH, C-5′′′), 171.1 (C, C-6′′′), 26.9 (CH_2_, C-7′′′′), as well as signals at *δ*_C_ 33.6 (CH_2_, C-8′′′′)/*δ*_H_ 1.52 (2H, m, H-8′′′′), *δ*_C_ 63.0 (CH_2_, C-9′′′′)/*δ*_H_ 3.53 (2H, t, *J* = 6.7 Hz, H-9′′′′) and *δ*_C_ 62.3 (CH_2_, C-1′′′′′)/*δ*_H_ 4.34 (2H, s, H-1′′′′′).

e Signals can be interchangeable.

f ov stands for overlapped signals.

Compounds 10–12 exhibited similar UV spectra as 9, indicative of pyrone derivatives (see ESI†). Their molecular formulae were determined as C_18_H_28_O_7_, C_19_H_30_O_7_ and C_17_H_28_O_6_, respectively, as indicated by their HRESIMS spectra. The ^1^H and ^13^C NMR data of 10–12 (Table 4) were similar to those of 9 and indicated that 10–12 retained the same α-pyrone core structure as 9, differing from the latter only in the nature of their aliphatic side-chains. The molecular weight of 10 was 44 amu larger than that of 9, which together with an additional methoxy signal at *δ*_H_ 3.65/*δ*_C_ 51.9 and an additional carbonyl resonating at *δ*_C_ 176.0 (C-9′), implied that the aliphatic chain of 10 is terminated by a carbomethoxy group instead of a methyl group. This deduction was corroborated by the observed HMBC correlation from H_2_-7′/H_2_-8′ to C-9′, in addition to a further degree of unsaturation of 10 in comparison with 9. The ^1^H NMR data of 11 (Table 4) displayed an additional oxygenated methylene at *δ*_H_ 4.05 (H_2_-9′) and a signal of a downfield shifted methyl group at *δ*_H_ 2.02 (H_3_-12′), which along with the observed HMBC correlations from H_2_-9′ to C-7′, C-8′ and C-11′, as well as from H_3_-12′ to C-11, hinted at the presence of an acetoxy group at the terminus of the aliphatic side-chain in 11. The molecular weight of compound 12 was 16 amu larger than that of 9. Moreover, the ^1^H NMR data of 12 (Table 4) displayed an extra oxygenated methylene (*δ*_H_ 3.53) split into a triplet, which revealed a COSY correlation to *δ*_H_ 1.52 (H-8′), suggesting the presence of a hydroxyl group at C-9′. Accordingly, compounds 10–12 were established as new α-pyrone analogues, for which the trivial names bulgariapyrones B–D are proposed.

**Fig. 5:**
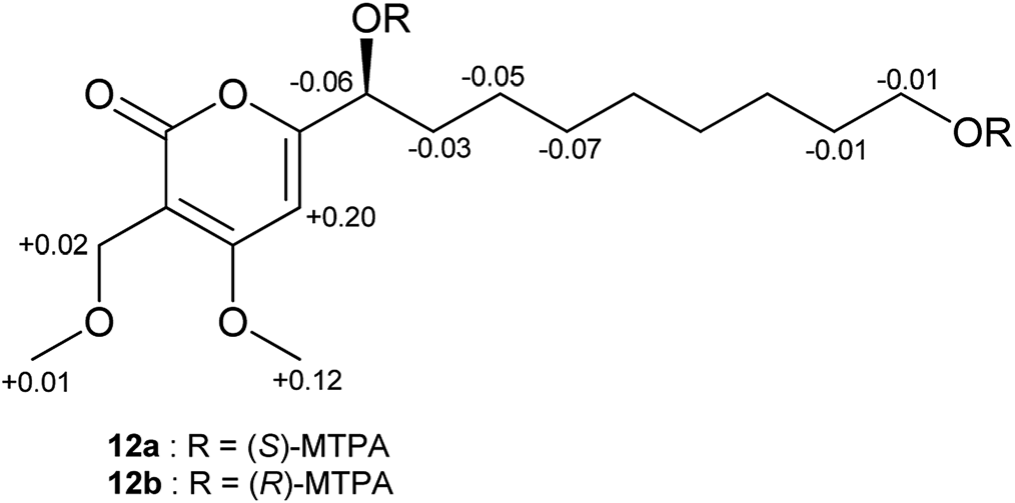
Δ*δ*_(*S*)–(*R*)_ values in ppm for the MTPA esters of 12.

In order to assign the absolute configurations of 9–12, the modified Mosher's reaction was carried out, and 12 was chosen as a model compound for this reaction. Both primary and secondary alcohol groups in 12 were converted to either (*S*)- or (*R*)-MTPA esters. Based on the calculated values of Δ*δ*_(*S*)–(*R*)_ of MTPA esters, the absolute configuration at C-1′ was established as (*S*), which is in agreement with the literature data of previously described α-pyrone derivatives related to 12 (Fig. 5).^47^ Similar specific optical rotation values of 9–11 suggested that these α-pyrones share the same (*S*) absolute configuration at C-1′.

The molecular formula of 13 was determined as C_33_H_52_O_11_, based on the prominent pseudomolecular ion peak at *m*/*z* 625.3587 [M + H]^+^ in the HRESIMS spectrum, corresponding to 8 degrees of unsaturation. Analysis of the molecular formula and of the 1D/2D NMR data of 13 (Table 4, ESI†) suggested it contained two substructures similar to bulgariapyrone D (12). However, the NMR data of 13 revealed additional signals of an isolated methylene at *δ*_H_ 4.34/*δ*_C_ 62.3 (H_2_-1′′′′′) and one set of methylene as a triplet at *δ*_H_ 3.46/*δ*_C_ 71.6 (H_2_-9′), suggesting an asymmetry of the molecule. The HMBC correlations observed from H_2_-1′′′′′ to C-2′′′, C-3′′′, C-4′′′ and C-9′, and in turn from H_2_-9′ to C-7′, C-8′ and C-1′′′′′, together with the NOESY correlation H_2_-9′/H_2_-1′′′′′ allowed connecting these two monomeric units *via* an ether bridge between C-1′′′′′ and C-9′, suggesting that 13 could be formed through a condensation reaction between two molecules of 12. Thus, the planar structure of 13 was elucidated as shown and the name bulgariapyrone E is proposed. Notably, only two related naturally occurring symmetrical α-pyrone dimers have been reported so far, multiforisin D, previously isolated from *Gelasinospora multiforis*,^50^ and dothideopyrone D, a metabolite from the endophytic fungus *Dothideomycete* sp. LRUB20,^49^ which emphasize the rare nature of these dimeric compounds. Moreover, compound 13 seems to be a product of a “head to tail” condensation, where the side chain of one monomeric unit is connected through an ether bond to the C-1′′′′′ of the α-pyrone nucleus of the other monomeric unit, which creates an asymmetry in the structure of 13 and makes it structurally different from known symmetrical C2-type bis-pyrone dimers. As the condensation of primary alcohols might take place under acidic conditions, an experiment was carried out by incubating 0.2 mg of 12 in 0.2 mL MeOH containing different concentrations of formic acid (0.1%, 1%, 5% and 10%), at room temperature for 1 week, followed by HPLC analysis. The HPLC chromatograms showed no formation of compound 13, which indicated that 13 is a true natural product, and not an artifact arising during the isolation procedure.

Following the same Mosher's reaction protocol for compound 13 as for 12, the (*S*)- or (*R*)-MTPA ester products were obtained (see the Experimental section). The MALDI-MS spectra of the reaction products indicated that all the primary and secondary alcohol groups in 13 reacted with the reagent (*m*/*z* 1295 [M + Na]^+^). Thus, the absolute configuration of 13 was unequivocally assigned as (*S*) for both stereocenters at C-1′ and C-1′′′′, which is in accordance with biosynthetic considerations and the reported configuration of the structurally related homodimer dothideopyrone D.^49^

Compound 14 was obtained as a dark red solid. Its molecular formula was determined as C_26_H_28_O_7_, on the basis of a prominent pseudomolecular ion peak at *m*/*z* 453.1910 [M + H]^+^ in the HRESIMS spectrum, requiring 13 degrees of unsaturation. Detailed analysis of the 1D and 2D NMR spectra showed that 14 is a known compound, bulgarialactone B,^29^ first isolated from the same fungus more than two decades ago. However, in this first paper, the absolute configuration was not determined for bulgarialactone B and the relative configuration was elucidated on the basis of the weak NOESY correlation between H-3 and H_3_-27. Further reports on bulgarialactone B focused on the Hsp90 inhibitory activity and were not engaged in the elucidation of the absolute configuration.^30,51^ The SciFinder database indicates the absolute configuration for the closely related compound epicocconone, which lacks a chirality centre in the unsaturated side-chain and exhibits potent fluorescent properties, but the original paper^52^ reported only the relative configuration of the two chirality centres as elucidated by the comparison of the HF/6-31G(d,p) optimized low-energy geometries of the high-temperature molecular dynamics trajectories with the experimental NMR data. Subsequent papers did not address the elucidation of the absolute configuration of epicocconone.

**Fig. 6:**
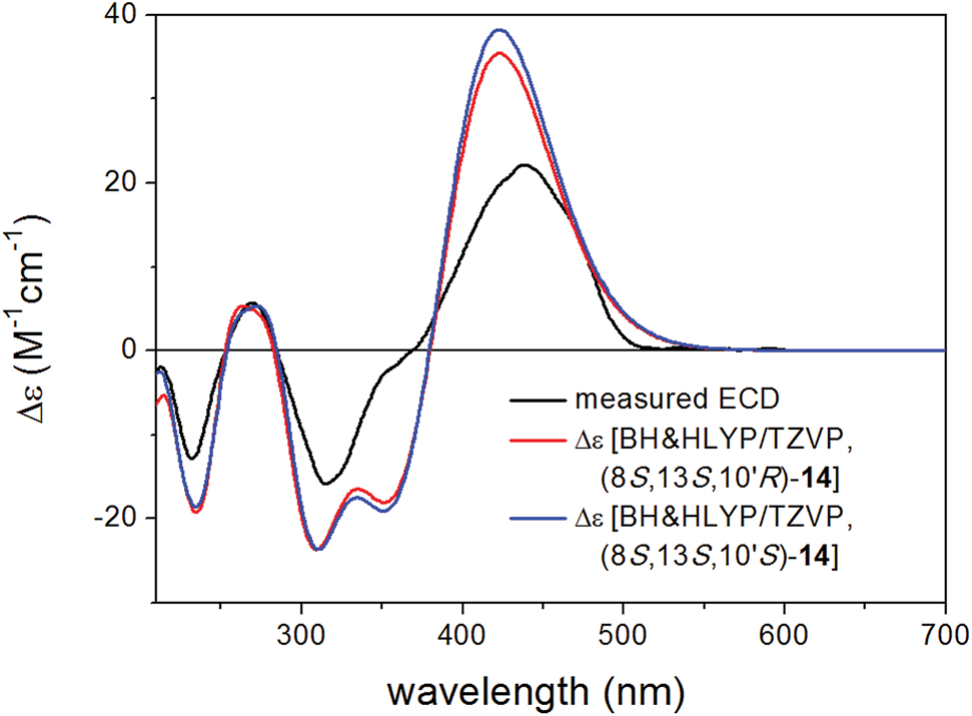
Experimental ECD spectrum of 14 in MeCN compared with the Boltzmann-weighted BH&HLYP/TZVP PCM/MeCN spectra of (3*S*,11*S*,23*R*)-14 and (3*S*,11*S*,23*S*)-14 computed for the low-energy (≥1%) CAM-B3LYP/TZVP PCM/MeCN conformers (26 and 26 conformers, respectively).

**Fig. 7:**
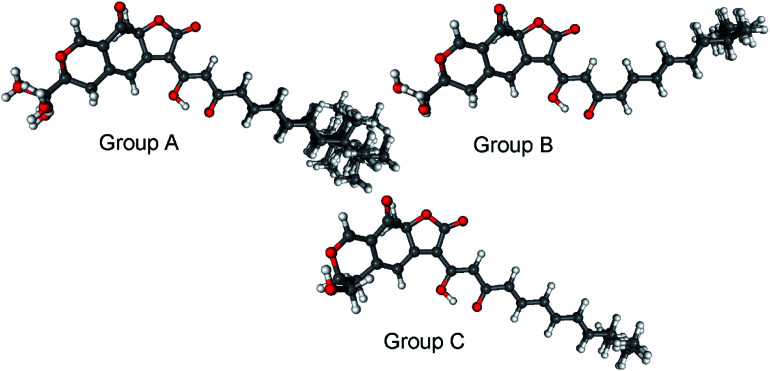
Classification of the 26 low-energy (≥1%) CAM-B3LYP/TZVP PCM/MeCN conformers of (3*S*,11*S*,23*R*)-14 into conformer groups. Group A (70.3%) contains conformers A, B, C, D, E, F, H, I, J, L, M, N, Q, R, S, T, U, V, W, Y, Z; group B (5.6%) contains conformers G, K, X; group C (3.2%) contains conformers O and P.

Bulgarialactone B (14) had positive Cotton effects (CEs) at 437 and 269 nm and negative ones at 315 and 232 nm. In order to determine the absolute configuration of bulgarialactone B, the solution TDDFT-ECD method was applied on the arbitrarily chosen (3*S*,11*S*,23*R*) and (3*S*,11*S*,23*S*) stereoisomers.^53,54^ Although the core part was expected to govern mostly the ECD spectrum, the C-23 chirality centre located in the allylic position of the conjugated π system of the side-chain was also supposed to have at least a minor contribution, which would have allowed distinguishing the C-23 epimers. A MMFF (Merck Molecular Force Field) conformational search of the two epimers resulted in 549 and 541 conformer clusters in a 21 kJ mol^−1^ energy window, respectively, indicating high conformational flexibility. These geometries were reoptimized at the CAM-B3LYP/TZVP PCM/MeCN level. Despite the large number of low-energy conformers, the Boltzmann-averaged ECD spectra of both epimers computed at four different levels (B3LYP/TZVP, BH&HLYP/TZVP, CAM-B3LYP/TZVP and PBE0/TZVP, all with PCM for MeCN) gave consistently moderate to good agreement with the experimental ECD spectrum (Fig. 6 and 7). Interestingly, the difference between the computed ECD spectra of the epimers was rather small, which suggested that the influence of C-23 on the overall ECD is marginal. Accordingly, the absolute configuration of the core part could be elucidated as (3*S*,11*S*), while the C-23 chirality centre remained unassigned. By analyzing the individual ECD spectra of the low-energy computed conformers, it turned out that the features of the ECD spectra were influenced by the helicity of the dihydropyrane ring and the orientation of the conjugating side-chain, the latter of which was also described by Syzgantseva *et al.* to influence the UV characteristics.^55^

The experimental ECD spectra of 14 and that of (+)-epicocconone reported by Bell and Karuso^52^ have a mirror image relationship, suggesting that (+)-epicocconone showing an intense negative CE above 400 nm has an (3*R*,11*R*) absolute configuration in *Epicoccum nigrum*,^52^ which is an example of the chiral switching of the tricyclic skeleton.^56^ It is also clear that the absolute configuration presented in the SciFinder database for epicocconone^57^ is incorrect and it should specify only the relative configuration.

The planar structure of compound 16 was shown to be identical to the previously described flavipesin B, isolated from the fungal endophyte *Aspergillus flavipes* AIL8,^58^ which has been previously reported as a synthetic compound.^59,60^ In a recent study, it was isolated from an engineered strain of *A. nidulans*.^61^ However, 16 possesses an opposite sign of the specific optical rotation, [*α*]^25^_D_ −72 (MeOH), compared to that of the reported value for flavipesin B: [*α*]^25^_D_ +133 (acetone).^58^ Therefore, in this study, we assigned 16 as (−)-(*S*)-flavipesin B.

The structures of the remaining known compounds isolated from *B. inquinans* were established as phenylbutyrolactone IIa (15),^33,34,59^ xenofuranone B (17)^35,67^ and bulgarialactone D (18),^30^ based on their spectroscopic data as well as on a comparison with the literature.

All the isolated compounds were evaluated for cytotoxicity towards the murine lymphoma cell line L5178Y. However, only 2 exhibited pronounced activity against the L5178Y cell line, with an IC_50_ value of 1.8 μM, which was stronger than that of the positive control kahalalide F (IC_50_ 4.3 μM). The presence of a hydroxy group attached to C-5 in the 2-furanone ring in the structure of 2 was apparently important for its cytotoxic activity as no activity was found for compounds 3, phenylbutyrolactone IIa (15), 16 and xenofuranone B (17). Furthermore, a closely related derivative, 4-*O*-demethylisobutyrolactone II, which bears an additional hydroxy group on both aromatic rings at the positions 9 and 16 (tyrosine-derived residues) compared to 2, was shown to be inactive against the L5178Y cell line,^62^ thus suggesting that the phenyl substituents were required for its cytotoxicity. The remaining compounds showed no significant cytotoxic properties. Interestingly, related α-pyrones were previously reported to be nontoxic or exhibited only moderate cytotoxicity against several cancer cell lines.^49^ A number of α-pyrone analogues, however, revealed promising biological activities as antibacterial agents,^63^ HIV-1 protease inhibitors,^64^ tyrosinase inhibitors^65^ or inhibitors of nitric oxide production,^47^ making these metabolites attractive scaffolds for synthetic chemical studies. In our study, bulgarialactone B (1) revealed no cytotoxicity against the tested L5178Y cell line, albeit in a previous study it was reported to possess antitumor activity against an ascitic ovarian carcinoma xenograft.^30^

Butyrolactone analogues similar to the derivatives isolated in this study were previously described from *Aspergillus* species,^58,62,66–68^ Here we report *B*. *inquinans* as a source of this type of metabolites for the first time. Moreover, the results obtained upon application of the OSMAC approach employing a mixture of salts (MgSO_4_, NaNO_3_ and NaCl) highlighted the usefulness of this strategy not only for diversifying secondary metabolites produced by this strain, but also to afford rare natural product scaffolds, as exemplified by the isolation of 1,3-oxazine derivatives (7 and 8) and of an unusual α-pyrone dimer 13. Another set of OSMAC experiments was performed to investigate which salt mainly contributes to the changes in the metabolic profile of *B*. *inquinans*, by adding separately MgSO_4_·7H_2_O (2.5 and 3.5 g), and a mixture of NaNO_3_ and NaCl (2.5 g each) to Czapek medium with and without MgSO_4_. The fungus failed to grow in the presence of the mixture of NaNO_3_ and NaCl, when MgSO_4_ was completely excluded from Czapek medium, suggesting that Mg^2+^ ions are apparently critical for fungal growth. However, the addition of MgSO_4_ alone did not result in any changes in the HPLC chromatogram in comparison with the chromatogram of the fungus grown on solid Czapek medium, suggesting that the fungus was only metabolically affected by adding the mixture of these three salts simultaneously. Interestingly, OSMAC studies with the marine-derived fungus, *Ascotricha* sp. ZJ-M-5, involving MgCl_2_ in Czapek Dox broth medium, previously reported that Mg^2+^ ions influenced the secondary metabolites profile of this fungus.^69^ With respect to the findings in this study, the compounds obtained from the OSMAC experiments in the presence of Mg^2+^ were mostly methoxylated derivatives. Thus, it may be speculated that Mg^2+^ ions under certain conditions might trigger *O*-alkylation reactions.

## Experimental section

### General procedures

A Jasco P-2000 polarimeter was used to measure the optical rotations. ^1^H (600 and 300 MHz), ^13^C (150, 125 and 75 MHz) and 2D NMR spectra were recorded on Bruker AVANCE DMX 600, 500 and 300 NMR spectrometers. The chemical shifts (*δ*) were referenced to the residual solvent peaks at *δ*_H_ 3.31 (MeOH-*d*_4_) ppm for ^1^H or *δ*_C_ 49.0 (MeOH-*d*_4_) ppm for ^13^C. Mass spectra (ESI) were measured with a Finnigan LCQ Deca mass spectrometer. HRESIMS spectra were recorded with an UHR-QTOF maXis 4G (Bruker Daltonics) mass spectrometer. HPLC analysis was performed with a Dionex UltiMate 3000 with an UltiMate 3000 pump coupled to a photodiode array detector (DAD 3000 RS). Detection wavelengths were set at 235, 254, 280 and 340 nm. The column was prefilled with Eurospher 100–10 C_18_, 125 × 4 mm (Knauer, Germany). The following gradient was used for routine analysis (MeOH: 0.1% HCOOH in H_2_O): 0 min (10% MeOH); 5 min (10% MeOH); 35 min (100% MeOH); 45 min (100% MeOH). Semipreparative HPLC was performed with a Merck Hitachi Chromaster HPLC system (UV detector 5410; pump 5110; column Eurospher 100–10 C_18_, 300 × 8 mm, Knauer, Germany; flow rate 5 mL min^−1^). Column chromatography was performed using Silica 60 M (0.040–0.063 mm; Macherey-Nagel, Germany), Silica gel 90 C_18_-reversed phase and Sephadex LH-20. TLC plates pre-coated with silica gel 60 F_254_ (Macherey-Nagel, Germany) were used for analysis, detection was under UV 254 and 366 nm. ECD spectra were recorded on a JASCO J-810 spectropolarimeter.

### Fungal material and fermentation

The fungus *Bulgaria inquinans* (isolate MSp3-1) was isolated from a healthy sprout of mistletoe (*Viscum album*),^70^ collected in January 2017 at Jülich, Germany. Fungal identification was carried out according to a standard molecular biology protocol,^70^ followed by a BlastN search in the NCBI database. The sequence was submitted to the GenBank (accession no. MK246763). The fungal strain is kept in one of the author's laboratory (P. P.).

The fungus was cultivated on solid Czapek medium, which was prepared by autoclaving 200 mL of liquid Czapek medium with the addition of 5.0 g of bacto agar in a 1 L Erlenmeyer flask. The composition of liquid Czapek medium was 10.0 g dextrose, 20.0 g mannitol, 20.0 g maltose, 3.0 g yeast extract, 1.0 g corn steep liquor, 0.5 g tryptophan, 0.5 g K_2_HPO_4_·3H_2_O, 0.3 g MgSO_4_·7H_2_O and 1 L of distilled water (pH value of the medium adjusted between 7.2–7.8). The fermentation was performed in 15 flasks at room temperature, under static conditions for 27 days.

OSMAC experiments were carried out by growing the fungus on solid Czapek medium containing either 3.5 g NaCl, 3.5 g NaBr, 3.5 g NaI, 3.5 g NaNO_3_, 3.5 g (NH_4_)_2_SO_4_ or mixtures of (a) MgSO_4_·7H_2_O, NaNO_3_ and NaCl (2.5 g of each), (b) FeSO_4_·7H_2_O, NaNO_3_ and NaCl (2.5 g of each), or (c) ZnSO_4_·7H_2_O, NaNO_3_ and NaCl (2.5 g of each), added to each 1 L flask followed by extraction when the flasks were completely overgrown by the fungus. Based on the chromatographic profiles obtained from these experiments, a large-scale fermentation of *B. inquinans* was carried out by adding a mixture of MgSO_4_·7H_2_O, NaNO_3_ and NaCl (2.5 g of each) to solid Czapek medium. The fungus was grown under static conditions for 33 days followed by extraction.

### Extraction and isolation

The fungal culture grown on solid Czapek medium was extracted with 500 mL EtOAc added to each flask followed by concentration *in vacuo* to afford the crude extract (5.1 g). The extract was then loaded on silica gel 60 (VLC) and eluted successively with *n*-hexane–EtOAc followed by CH_2_Cl_2_–MeOH to obtain 13 fractions (V1–V13). Fractions V3, V4 and V5 eluted with *n*-hexane–EtOAc (6 : 4), (4 : 6) and (2 : 8), respectively, were subjected to further separation based on their HPLC chromatograms. Fraction V5 (1.7 g) was separated over Sephadex LH-20 and eluted with MeOH to afford nine subfractions (V5.1–V5.9). Purification of the V5.3 subfraction (65.9 mg) was achieved by semipreparative HPLC using ACN–H_2_O (from 65% to 100% ACN, 20 min), to yield bulgarialactone B (14, 27.0 mg). Fraction V4 (507.9 mg) was submitted to Sephadex LH-20, employing CH_2_Cl_2_–MeOH (1 : 1) as the mobile phase to obtain 12 subfractions (V4.1–V4.12). Semipreparative HPLC was used to purify the subfraction V4.10 (270.0 mg), using MeOH-0.1% HCOOH in H_2_O (from 30% to 100% MeOH, 25 min), to afford the new compounds 1 (20.0 mg) and 4 (2.2 mg) along with phenylbutyrolactone IIa (15, 124.0 mg). In a similar manner, the separation of fraction V3 (150 mg) on Sephadex LH-20 resulted in 10 subfractions (V3.1–V3.10). Purification by semipreparative HPLC of the V3.6 subfraction (26.0 mg), employing MeOH-0.1% HCOOH in H_2_O as the eluent (from 65% to 100% MeOH, 20 min), yielded the new natural products 2 (2.8 mg), 3 (1.5 mg) and 5 (1.9 mg) together with xenofuranone B (17, 1.2 mg).

The fungal culture obtained from the large-scale fermentation on the Czapek medium with the salt mixture was extracted with 500 mL EtOAc added to each flask. Following the previously described procedure, the crude extract (7.7 g) obtained after removal of the solvent was chromatographed on Silica gel 60 (VLC) to afford 13 fractions (MV1–MV13). Fractions MV3, MV4, MV6 and MV9 eluted with *n*-hexane–EtOAc (6 : 4), (4 : 6), 100% EtOAc and DCM–MeOH (1 : 9), respectively, were selected for further isolation work-up, guided by their HPLC results. The separation of fraction MV9 (1.5 g) was carried out on a Silica gel 90 C_18_-reversed phase column by a step gradient elution employing mixtures of H_2_O–MeOH to yield 10 subfractions (MV9.1–MV9.10). The MV9.7 subfraction (95.1 mg) was submitted to Sephadex LH-20 using CH_2_Cl_2_–MeOH (1 : 1) as the eluent to yield seven subfractions (MV9.7.1–MV9.7.7). Purification of the MV9.7.2 subfraction (24.5 mg) was achieved by semipreparative HPLC using MeOH-0.1% HCOOH in H_2_O (from 65% to 100% MeOH, 20 min) to afford 13 (9.3 mg). The new compound 12 (30.4 mg) was afforded by the purification of MV9.7.5 (64.7 mg) with semipreparative HPLC using MeOH-0.1% HCOOH in H_2_O (from 40% to 100% MeOH, 20 min). The new compound 8 (1.5 mg) was obtained by the separation of the MV9.1 subfraction (40.0 mg) on Sephadex LH-20, and final purification was achieved by semipreparative HPLC using MeOH-0.1% HCOOH in H_2_O (from 25% to 100% MeOH, 20 min). Fraction MV6 (452.5 mg) was applied on Sephadex LH-20 employing MeOH as the eluent to yield nine subfractions (MV6.1–MV6.9). Purification of the MV6.6 subfraction (21.0 mg) was carried out by semipreparative HPLC using ACN–H_2_O (from 60% to 100% ACN, 25 min) to afford bulgarialactone D (18, 4.0 mg). The new compounds 9 (2.6 mg), 10 (9.1 mg) and 11 (2.6 mg) were obtained from purification of MV6.3 subfraction (59.3 mg) by semipreparative HPLC using MeOH-0.1% HCOOH in H_2_O (from 55% to 100% MeOH, 20 min). Furthermore, fraction MV4 (622.4 mg) was chromatographed on Sephadex LH-20, eluting with CH_2_Cl_2_–MeOH (1 : 1), to give eight subfractions (MV4.1–MV4.8). The MV4.6 subfraction (54.4 mg) was then purified by semipreparative HPLC using MeOH-0.1% HCOOH in H_2_O (from 40% to 100% MeOH, 25 min), which yielded 3 (14.6 mg) and 6 (3.2 mg) along with 16 (6.5 mg). Application of the latter procedure for the purification of fraction MV3 (164.9 mg) by semipreparative HPLC following separation on Sephadex LH-20 yielded compounds 5 (2.8 mg) and 7 (2.0 mg). The total amounts of 3 and 5, both from the fungal culture grown on solid Czapek medium and from the OSMAC experiment, were 16.1 and 4.7 mg, respectively.

Bulgariline A (1): yellow solid; [*α*]^20^_D_ +16 (*c* 0.20, MeOH); UV (MeOH, PDA): *λ*_max_ 288, 218 nm; ECD (MeCN, *λ* [nm] (Δ*ε*), *c* 0.529 mM): 294sh (+0.56), 282 (+0.71), 254 (−0.44), 232 (+1.05), 221 (−0.74), 205sh (+2.42); ^1^H and ^13^C NMR data, see Table 1; HRESIMS *m*/*z* 235.0600 [M + H]^+^ (calcd for C_12_H_11_O_5_, 235.0601).

Bulgariline B (2): yellow solid; [*α*]^20^_D_ 0 (*c* 0.20, MeOH); UV (MeOH, PDA): *λ*_max_ 291, 204 nm; ^1^H and ^13^C NMR data, see Table 1; HRESIMS *m*/*z* 283.0962 [M + H]^+^ (calcd for C_17_H_15_O_4_, 283.0965).

Bulgariline C (3): yellow solid; [*α*]^20^_D_ 0 (*c* 0.20, MeOH); UV (MeOH, PDA): *λ*_max_ 293, 201 nm; ^1^H and ^13^C NMR data, see Table 1; HRESIMS *m*/*z* 297.1123 [M + H]^+^ (calcd for C_18_H_17_O_4_, 297.1121).

Bulgariol (4): yellow, solid; [*α*]^20^_D_ −25 (*c* 0.20, MeOH); UV (MeOH, PDA): *λ*_max_ 277 nm; ECD (MeCN, *λ* [nm] (Δ*ε*), *c* 0.409 mM): 280 (−0.19), 245sh (+0.10), 225 (+0.95), 201sh (+1.52), 195 (+2.55); ^1^H and ^13^C NMR data, see Table 2; HRESIMS *m*/*z* 267.0989 [M + Na]^+^ (calcd for C_15_H_16_NaO_3_, 267.0992).

(5*Z*)-3-Hydroxy-4-phenyl-5-(phenylmethylene)-2(5*H*)-furanone (5): yellow solid; UV (MeOH, PDA): *λ*_max_ 336, 263, 245 nm; ^1^H and ^13^C NMR data, see Table 1; HRESIMS *m*/*z* 265.0861 [M + H]^+^ (calcd for C_17_H_13_O_3_, 265.0859).

Bulgariline D (6): yellow solid; [*α*]^25^_D_ +26 (*c* 0.20, MeOH); UV (MeOH, PDA): *λ*_max_ 287, 217 nm; ECD (MeCN, *λ* [nm] (Δ*ε*), *c* 0.201 mM): 296sh (+2.70), 281 (+3.94), 253sh (−0.95), 249 (−1.18), 231 (+2.99), 221 (−3.22), 218sh (−2.90), 204sh (+7.03); ^1^H and ^13^C NMR data, see Table 1; HRESIMS *m*/*z* 249.0757 [M + H]^+^ (calcd for C_13_H_13_O_5_, 249.0757).

Bulgarixine A (7): yellow solid; [*α*]^25^_D_ 0 (*c* 0.11, MeOH); UV (MeOH, PDA): *λ*_max_ 243, 206 nm; ^1^H and ^13^C NMR data, see Table 3; HRESIMS *m*/*z* 248.0919 [M + H]^+^ (calcd for C_13_H_14_NO_4_, 248.0917).

Bulgarixine B (8): yellow solid; [*α*]^25^_D_ 0 (*c* 0.20, MeOH); UV (MeOH, PDA): *λ*_max_ 244, 206 nm; ^1^H and ^13^C NMR data, see Table 3; HRESIMS *m*/*z* 234.0760 [M + H]^+^ (calcd for C_12_H_12_NO_4_, 234.0761).

Bulgariapyrone A (9): brown solid; [*α*]^25^_D_ −48 (*c* 0.20, MeOH); UV (MeOH, PDA): *λ*_max_ 300, 206 nm; ^1^H and ^13^C NMR data, see Table 4; HRESIMS *m*/*z* 313.2013 [M + H]^+^ (calcd for C_17_H_29_O_5_, 313.2010).

Bulgariapyrone B (10): brown solid; [*α*]^25^_D_ −72 (*c* 0.40, MeOH); UV (MeOH, PDA): *λ*_max_ 299, 211 nm; ^1^H and ^13^C NMR data, see Table 4; HRESIMS *m*/*z* 357.1919 [M + H]^+^ (calcd for C_18_H_29_O_7_, 357.1908).

Bulgariapyrone C (11): brown solid; [*α*]^25^_D_ −70 (*c* 0.20, MeOH); UV (MeOH, PDA): *λ*_max_ 300, 206 nm; ^1^H and ^13^C NMR data, see Table 4; HRESIMS *m*/*z* 371.2070 [M + H]^+^ (calcd for C_19_H_31_O_7_, 371.2064).

Bulgariapyrone D (12): brown solid; [*α*]^25^_D_ −90 (*c* 0.20, MeOH); UV (MeOH, PDA): *λ*_max_ 298, 212 nm; ^1^H and ^13^C NMR data, see Table 4; HRESIMS *m*/*z* 329.1960 [M + H]^+^ (calcd for C_17_H_29_O_6_, 329.1959).

Bulgariapyrone E (13): brown solid; [*α*]^25^_D_ −32 (*c* 0.20, MeOH); UV (MeOH, PDA): *λ*_max_ 299, 208 nm; ^1^H and ^13^C NMR data, see Table 4; HRESIMS *m*/*z* 625.3587 [M + H]^+^ (calcd for C_33_H_53_O_11_, 625.3582).

Bulgarialactone B (14): dark red solid; [*α*]^20^_D_ +344 (*c* 0.10, CHCl_3_); UV (MeOH, PDA): *λ*_max_ 441, 322 nm; ECD (MeCN, *λ* [nm] (Δ*ε*), *c* 0.147 mM): 470sh (+14.51), 437 (+22.20), 417sh (+18.22), 352sh (−2.59), 323sh (−14.77), 315 (−15.84), 269 (+5.71), 232 (−12.87); HRESIMS *m*/*z* 453.1910 [M + H]^+^ (calcd for C_26_H_29_O_7_, 453.1908).

(−)-(*S*)-Flavipesin B (16): yellow solid; [*α*]^25^_D_ −72 (*c* 0.20, MeOH); UV (MeOH, PDA): *λ*_max_ 290, 204 nm; ^1^H and ^13^C NMR data, see ESI;† HRESIMS *m*/*z* 325.1073 [M + H]^+^ (calcd for C_19_H_17_O_5_, 325.1071).

### Mosher ester analysis of 12 and 13

Both (*S*)- and (*R*)-MTPA esters of 12 were prepared in NMR tubes by the addition of either (*R*)-MTPA-Cl (10 μL, 53.44 μmol) or (*S*)-MTPA-Cl (10 μL, 53.44 μmol) to a solution of 12 (1.0 mg, 3.05 μmol) and pyridine-*d*_5_ (10 μL, 130.75 μmol) in 100 μL CDCl_3_, according to a protocol described earlier.^71^ Each reaction mixture was maintained for 3 h at room temperature, and 500 μL CDCl_3_ was added afterwards. In a similar manner, (*S*)- and (*R*)-MTPA esters of 13 were prepared. Ester products were confirmed by LC-ESIMS at *m*/*z* 761 [M + H]^+^ for 12 and by MALDI-MS at *m*/*z* 1295 [M + Na]^+^ for 13.

(*S*)-MTPA ester of 12 (12a): ^1^H NMR (CDCl_3_, 600 MHz) *δ*_H_ 6.19 (1H, s, H-5), 5.63 (1H, dd, *J* = 7.5, 5.3 Hz, H-1′), 4.30 (1H, m, Hb-9′), 4.29 (2H, s, H_2_-1′′), 4.26 (1H, m, Ha-9′), 3.80 (3H, s, 4-OMe), 3.52 (3H, s, OMe), 3.48 (3H, s, OMe), 3.36 (3H, s, 1′′-OMe), 1.88 (2H, m, H_2_-2′), 1.64 (2H, p, *J* = 6.8 Hz, H_2_-8′), 1.19 (2H, m, H_2_-3′), 1.25 (2H, m, H_2_-7′), 1.13–1.25 (6H, m, H_2_-4′–H_2_-6′).

(*R*)-MTPA ester of 12 (12b): ^1^H NMR (CDCl_3_, 600 MHz) *δ*_H_ 5.99 (1H, s, H-5), 5.69 (1H, dd, *J* = 7.3, 5.0 Hz, H-1′), 4.31 (1H, m, Hb-9′), 4.27 (2H, s, H_2_-1′′), 4.27 (1H, m, Ha-9′), 3.68 (3H, s, 4-OMe), 3.54 (3H, s, OMe), 3.52 (3H, s, OMe), 3.35 (3H, s, 1′′-OMe), 1.91 (2H, m, H_2_-2′), 1.65 (2H, p, *J* = 6.8 Hz, H_2_-8′), 1.26 (2H, m, H_2_-3′) 1.25 (2H, m, H_2_-7′) 1.17–1.30 (6H, m, H_2_-4′–H_2_-6′).

(*S*)-MTPA ester of 13 (13a): ^1^H NMR (CDCl_3_, 600 MHz) *δ*_H_ 6.20 (1H, s, H-5/H-5′′′), 6.18 (1H, s, H-5′′′/H-5), 5.63 (2H, m, H-1′, H-1′′′′), 4.31 (2H, s, H_2_-1′′′′′), 4.31 (1H, m, Hb-9′′′′) 4.30 (2H, s, H_2_-1′′), 4.27 (1H, m, Ha-9′′′′) 3.82 (3H, s, 4-OMe/4′′′-OMe), 3.80 (3H, s, 4′′′-OMe/4-OMe), 3.53 (3H, s, OMe), 3.49 (6H, s, OMe), 3.47 (2H, t, *J* = 6.8 Hz, H_2_-9′), 3.37 (3H, s, 1′′-OMe), 1.90 (4H, m, H_2_-2′, H_2_-2′′′′), 1.65 (2H, p, *J* = 6.7 Hz, H_2_-8′/H_2_-8′′′′), 1.54 (2H, p, *J* = 6.7 Hz, H_2_-8′′′′/H_2_-8′), 1.27 (4H, m, H_2_-7′, H_2_-7′′′′), 1.21 (4H, m, H_2_-3′, H_2_-3′′′′), 1.16–1.25 (12H, m, H_2_-4′–H_2_-6′, H_2_-4′′′′–H_2_-6′′′′).

(*R*)-MTPA ester of 13 (13b): ^1^H NMR (CDCl_3_, 600 MHz) *δ*_H_ 5.99 (2H, s, H-5, H-5′′′), 5.68 (2H, m, H-1′, H-1′′′′), 4.31 (1H, m, Hb-9′′′′), 4.30 (2H, s, H_2_-1′′), 4.27 (1H, m, Ha-9′′′′), 4.28 (2H, s, H_2_-1′′′′′), 3.68 (3H, s, 4-OMe/4′′′-OMe), 3.67 (3H, s, 4′′′-OMe/4-OMe), 3.54 (6H, s, OMe), 3.53 (3H, s, OMe), 3.45 (2H, t, *J* = 6.8 Hz, H_2_-9′), 3.36 (3H, s, 1′′-OMe), 1.91 (4H, m, H_2_-2′, H_2_-2′′′′), 1.66 (2H, p, *J* = 6.7 Hz, H_2_-8′/H_2_-8′′′′), 1.54 (2H, p, *J* = 6.7 Hz, H_2_-8′′′′/H_2_-8′), 1.29 (4H, m, H_2_-3′, H_2_-3′′′′), 1.27 (4H, m, H_2_-7′, H_2_-7′′′′), 1.16–1.25 (12H, m, H_2_-4′–H_2_-6′, H_2_-4′′′′–H_2_-6′′′′).

### Cytotoxicity assay

Cytotoxicity was assayed against the murine lymphoma cell line L5178Y, using the MTT method.^62^ Kahalalide F was used as a positive control and a medium containing 0.1% DMSO was included as a negative control.

## Computational section

Mixed torsional/low-mode conformational searches were carried out by using the Macromodel 10.8.011 software^72^ with the Merck Molecular Morce Field (MMFF) with an implicit solvent model for CHCl_3_ applying a 21 kJ mol^−1^ energy window. Geometry optimizations [B3LYP/6-31+G(d,p) *in vacuo* and CAM-B3LYP/TZVP^73^ with the PCM solvent model for MeCN] and TDDFT [B3LYP/TZVP, BH&HLYP/TZVP, CAM-B3LYP/TZVP and PBE0/TZVP with the same or no solvent model as in the preceding optimization step] calculations were performed with Gaussian 09.^74^ The ECD spectra were generated as the sum of the Gaussians with 3000 and 4200 cm^−1^ half-height widths using dipole-velocity-computed rotational strength values.^75^ Boltzmann distributions were estimated from the B3LYP and the CAM-B3LYP energies. The MOLEKEL software package was used for visualization of the results.^76^

## Conflicts of interest

There are no conflicts to declare.

## Supplementary Material

RA-009-C9RA03678D-s001
