# Supplementary material for: Expanding the chemical diversity of an endophytic fungus Bulgaria inquinans, an ascomycete associated with mistletoe, through an OSMAC approach
Source: RSC Adv. 2019 Aug 13;9(43):25119–32. doi: 10.1039/c9ra03678d (PMC9069884; doi:10.1039/c9ra03678d)
Supplement: RA-009-C9RA03678D-s001 [file RA-009-C9RA03678D-s001.pdf]

Electronic Supplementary Material (ESI) for RSC Advances.
This journal is © The Royal Society of Chemistry 2019

Supporting Information

Expanding the Chemical Diversity of an Endophytic Fungus *Bulgaria inquinans*, an Ascomycete Associated with Mistletoe, through OSMAC Approach

Ni P. Ariantari,[ab] Georgios Daletos,[a] Attila Mándi,[c] Tibor Kurtán,[c] Werner E. G. Müller,[d] Wenhan Lin,[e] Elena Ancheeva,*[a] Peter Proksch*[a]

[a]Institute of Pharmaceutical Biology and Biotechnology, Heinrich Heine University Düsseldorf, Universitätsstrasse 1, 40225 Düsseldorf, Germany

[b]Department of Pharmacy, Faculty of Mathematic and Natural Sciences, Udayana University, 80361 Bali, Indonesia

[c]Department of Organic Chemistry, University of Debrecen, P.O.B. 400, 4002 Debrecen, Hungary

[d]Institute of Physiological Chemistry, Universitätsmedizin der Johannes Gutenberg-Universität Mainz, Duesbergweg 6, 55128 Mainz, Germany

[e]State Key Laboratory of Natural and Biomimetic Drugs, Peking University, 100191 Beijing, China

[*]Corresponding author

Table of Contents

	Page
**Figure S1**. HPLC chromatograms of EtOAc extract of *B. inquinans* (isolate MSp3-1) cultured on solid Czapek medium (black) compared to the OSMAC culture on solid Czapek medium with addition of a mixture of $MgSO_4$, $NaNO_3$ and NaCl (blue) under UV detection at 280 nm.	5
**Figure S2**. HPLC chromatogram (A) and UV spectrum (B) of compound **1**.	6
**Figure S3**. HRESIMS spectrum of compound **1**.	6
**Figure S4**. ^{1}H NMR (600 MHz, MeOH-d_4) spectrum of compound **1**.	7
**Figure S5**. ^{13}C NMR (150 MHz, MeOH-d_4) spectrum of compound **1**.	7
**Figure S6**. ^{1}H-^{1}H COSY (600 MHz, MeOH-d_4) spectrum of compound **1**.	8
**Figure S7**. HSQC (600 and 150 MHz, MeOH-d_4) spectrum of compound **1**.	8
**Figure S8**. HMBC (600 and 150 MHz, MeOH-d_4) spectrum of compound **1**.	9
**Figure S9**. NOESY (600 MHz, MeOH-d_4) spectrum of compound **1**.	9
**Figure S10**. HPLC chromatogram (A) and UV spectrum (B) of compound **2**.	10
**Figure S11**. HRESIMS spectrum of compound **2**.	10
**Figure S12**. ^{1}H NMR (600 MHz, MeOH-d_4) spectrum of compound **2**.	11
**Figure S13**. ^{13}C NMR (150 MHz, MeOH-d_4) spectrum of compound **2**.	11
**Figure S14**. ^{1}H-^{1}H COSY (600 MHz, MeOH-d_4) spectrum of compound **2**.	12
**Figure S15**. HSQC (600 and 150 MHz, MeOH-d_4) spectrum of compound **2**.	12
**Figure S16**. HMBC (600 and 150 MHz, MeOH-d_4) spectrum of compound **2**.	13
**Figure S17**. HPLC chromatogram (A) and UV spectrum (B) of compound **3**.	13
**Figure S18**. HRESIMS spectrum of compound **3**.	14
**Figure S19**. ^{1}H NMR (600 MHz, MeOH-d_4) spectrum of compound **3**.	14
**Figure S20**. ^{13}C NMR (150 MHz, MeOH-d_4) spectrum of compound **3**.	15
**Figure S21**. ^{1}H-^{1}H COSY (600 MHz, MeOH-d_4) spectrum of compound **3**.	15
**Figure S22**. HSQC (600 and 150 MHz, MeOH-d_4) spectrum of compound **3**.	16
**Figure S23**. HMBC (600 and 150 MHz, MeOH-d_4) spectrum of compound **3**.	16
**Figure S24**. NOESY (600 MHz, MeOH-d_4) spectrum of compound **3**.	17
**Figure S25**. HPLC chromatogram (A) and UV spectrum (B) of compound **4**.	17
**Figure S26**. HRESIMS spectrum of compound **4**.	18
**Figure S27**. ^{1}H NMR (600 MHz, MeOH-d_4) spectrum of compound **4**.	18
**Figure S28**. ^{13}C NMR (150 MHz, MeOH-d_4) spectrum of compound **4**.	19
**Figure S29**. ^{1}H-^{1}H COSY (600 MHz, MeOH-d_4) spectrum of compound **4**.	19
**Figure S30**. HSQC (300 and 75 MHz, MeOH-d_4) spectrum of compound **4**.	20
**Figure S31**. HMBC (300 and 75 MHz, MeOH-d_4) spectrum of compound **4**.	20
**Figure S32**. HPLC chromatogram (A) and UV spectrum (B) of compound **5**.	21
**Figure S33**. HRESIMS spectrum of compound **5**.	21
**Figure S34**. ^{1}H NMR (600 MHz, MeOH-d_4) spectrum of compound **5**.	22
**Figure S35**. ^{13}C NMR (150 MHz, MeOH-d_4) spectrum of compound **5**.	22
**Figure S36**. ^{1}H-^{1}H COSY (300 MHz, MeOH-d_4) spectrum of compound **5**.	23
**Figure S37**. HSQC (300 and 75 MHz, MeOH-d_4) spectrum of compound **5**.	23
**Figure S38**. HMBC (600 and 150 MHz, MeOH-d_4) spectrum of compound **5**.	24
**Figure S39**. NOESY (600 MHz, MeOH-d_4) spectrum of compound **5**.	24
**Figure S40**. HPLC chromatogram (A) and UV spectrum (B) of compound **6**.	25
**Figure S41**. HRESIMS spectrum of compound **6**.	25
**Figure S42**. ^{1}H NMR (600 MHz, MeOH-d_4) spectrum of compound **6**.	26
**Figure S43**. ^{13}C NMR (150 MHz, MeOH-d_4) spectrum of compound **6**.	26
**Figure S44**. ^{1}H-^{1}H COSY (600 MHz, MeOH-d_4) spectrum of compound **6**.	27

Figure S45. HSQC (600 and 150 MHz, MeOH-d_4) spectrum of compound **6**. 27
Figure S46. HMBC (600 and 150 MHz, MeOH-d_4) spectrum of compound **6**. 28
Figure S47. HPLC chromatogram (A) and UV spectrum (B) of compound **7**. 28
Figure S48. HRESIMS spectrum of compound **7**. 29
Figure S49. ^{1}H NMR (600 MHz, MeOH-d_4) spectrum of compound **7**. 29
Figure S50. ^{13}C NMR (150 MHz, MeOH-d_4) spectrum of compound **7**. 30
Figure S51. ^{1}H-^{1}H COSY (600 MHz, MeOH-d_4) spectrum of compound **7**. 30
Figure S52. HSQC (600 and 150 MHz, MeOH-d_4) spectrum of compound **7**. 31
Figure S53. HMBC (600 and 150 MHz, MeOH-d_4) spectrum of compound **7**. 31
Figure S54. HPLC chromatogram (A) and UV spectrum (B) of compound **8**. 32
Figure S55. HRESIMS spectrum of compound **8**. 32
Figure S56. ^{1}H NMR (600 MHz, MeOH-d_4) spectrum of compound **8**. 33
Figure S57. ^{1}H-^{1}H COSY (600 MHz, MeOH-d_4) spectrum of compound **8**. 33
Figure S58. HSQC (600 and 150 MHz, MeOH-d_4) spectrum of compound **8**. 34
Figure S59. HMBC (600 and 150 MHz, MeOH-d_4) spectrum of compound **8**. 34
Figure S60. HPLC chromatogram (A) and UV spectrum (B) of compound **9**. 35
Figure S61. HRESIMS spectrum of compound **9**. 35
Figure S62. ^{1}H NMR (600 MHz, MeOH-d_4) spectrum of compound **9**. 36
Figure S63. ^{1}H-^{1}H COSY (600 MHz, MeOH-d_4) spectrum of compound **9**. 36
Figure S64. HSQC (600 and 150 MHz, MeOH-d_4) spectrum of compound **9**. 37
Figure S65. HMBC (600 and 150 MHz, MeOH-d_4) spectrum of compound **9**. 37
Figure S66. HPLC chromatogram (A) and UV spectrum (B) of compound **10**. 38
Figure S67. HRESIMS spectrum of compound **10**. 38
Figure S68. ^{1}H NMR (600 MHz, MeOH-d_4) spectrum of compound **10**. 39
Figure S69. ^{13}C NMR (150 MHz, MeOH-d_4) spectrum of compound **10**. 39
Figure S70. ^{1}H-^{1}H COSY (600 MHz, MeOH-d_4) spectrum of compound **10**. 40
Figure S71. HSQC (600 and 150 MHz, MeOH-d_4) spectrum of compound **10**. 40
Figure S72. HMBC (600 and 150 MHz, MeOH-d_4) spectrum of compound **10**. 41
Figure S73. HPLC chromatogram (A) and UV spectrum (B) of compound **11**. 41
Figure S74. HRESIMS spectrum of compound **11**. 42
Figure S75. ^{1}H NMR (600 MHz, MeOH-d_4) spectrum of compound **11**. 42
Figure S76. ^{13}C NMR (150 MHz, MeOH-d_4) spectrum of compound **11**. 43
Figure S77. ^{1}H-^{1}H COSY (600 MHz, MeOH-d_4) spectrum of compound **11**. 43
Figure S78. HSQC (600 and 150 MHz, MeOH-d_4) spectrum of compound **11**. 44
Figure S79. HMBC (600 and 150 MHz, MeOH-d_4) spectrum of compound **11**. 44
Figure S80. HPLC chromatogram (A) and UV spectrum (B) of compound **12**. 45
Figure S81. HRESIMS spectrum of compound **12**. 45
Figure S82. ^{1}H NMR (600 MHz, MeOH-d_4) spectrum of compound **12**. 46
Figure S83. ^{13}C NMR (150 MHz, MeOH-d_4) spectrum of compound **12**. 46
Figure S84. ^{1}H-^{1}H COSY (600 MHz, MeOH-d_4) spectrum of compound **12**. 47
Figure S85. HSQC (600 and 150 MHz, MeOH-d_4) spectrum of compound **12**. 47
Figure S86. HMBC (600 and 150 MHz, MeOH-d_4) spectrum of compound **12**. 48
Figure S87. NOESY (300 MHz, MeOH-d_4) spectrum of compound **12**. 48
Figure S88. DEPT 135 (125 MHz, MeOH-d_4) spectrum of compound **12**. 49
Figure S89. HPLC chromatogram (A) and UV spectrum (B) of compound **13**. 49
Figure S90. HRESIMS spectrum of compound **13**. 50
Figure S91. ^{1}H NMR (600 MHz, MeOH-d_4) spectrum of compound **13**. 50
Figure S92. ^{13}C NMR (125 MHz, MeOH-d_4) spectrum of compound **13**. 51
Figure S93. ^{1}H-^{1}H COSY (600 MHz, MeOH-d_4) spectrum of compound **13**. 51
Figure S94. HSQC (600 and 150 MHz, MeOH-d_4) spectrum of compound **13**. 52
Figure S95. HMBC (600 and 150 MHz, MeOH-d_4) spectrum of compound **13**. 52

Figure S96. NOESY (600 MHz, MeOH-d_4) spectrum of compound **13**. 53
Figure S97. HPLC chromatogram (A) and UV spectrum (B) of compound **16**. 53
Figure S98. HRESIMS spectrum of compound **16**. 54
Figure S99. ^{1}H NMR (600 MHz, MeOH-d_4) spectrum of compound **16**. 54
Figure S100. ^{13}C NMR (150 MHz, MeOH-d_4) spectrum of compound **16**. 55
Figure S101. ^{1}H-^{1}H COSY (600 MHz, MeOH-d_4) spectrum of compound **16**. 55
Figure S102. HSQC (600 and 150 MHz, MeOH-d_4) spectrum of compound **16**. 56
Figure S103. HMBC (600 and 150 MHz, MeOH-d_4) spectrum of compound **16**. 56
Figure S104. Structure and population of the low-energy CAM-B3LYP/TZVP PCM/MeCN conformers (≥ 1%) of (*S*)-**1**. 57
Figure S105. Classification of the twenty-two low-energy (≥ 1%) CAM-B3LYP/TZVP PCM/MeCN conformers of (8*R*,9*S*)-**4**. Group A (44.9%) contains conformers A, B, E, F; group B (27.5%) contains conformers C, D, G, H, K, L, Q, R; group C (10.1%) contains conformers I, J, M, N; group D (3.2%) contains conformers O, P; group E (4.4%) contains conformers S, T, U and V. 57
Figure S106. Experimental ECD spectrum (black) of **6** in MeCN compared with the Boltzmann-weighted PBE0/TZVP PCM/MeCN ECD spectrum (purple) of (*R*)-**6** computed for the 6 low-energy CAM-B3LYP/TZVP PCM/MeCN conformers. The bars represent the rotational strength of the lowest-energy conformer. 58
Figure S107. Classification of the twenty-six low-energy (≥ 1%) CAM-B3LYP/TZVP PCM/MeCN conformers of (3*S*,11*S*,23*S*)-**14** into conformer groups. Group A (72.5%) contains conformers A, B, C, D, E, F, G, I, J, K, M, N, O, Q, R, S, T, U, V, W, Y, Z; group B (5.0%) contains conformers H, L, X; group C (1.6%) contains conformer P. 58

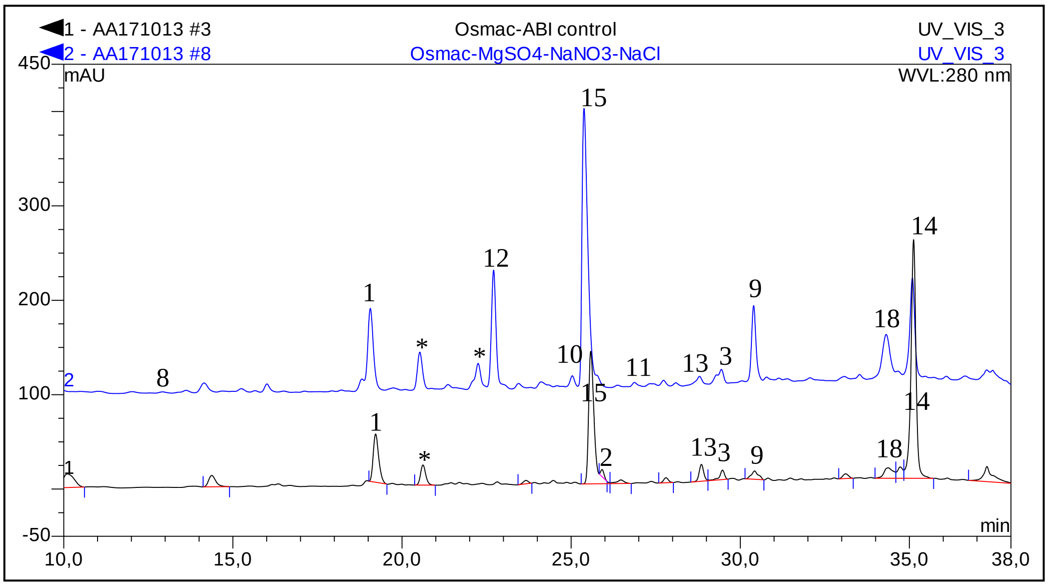

Figure S1. HPLC chromatograms of EtOAc extract of *B. inquinans* (isolate MSp3-1) cultured on solid Czapek medium (black) compared to the OSMAC culture on solid Czapek medium with addition of a mixture of $MgSO_4$, $NaNO_3$ and NaCl (blue) under UV detection at 280 nm.

*: unidentified peaks

Compounds **4**, **5**, **6**, **7**, **16** and xenofuranone B (**17**) were not detected neither in the HPLC analysis of the crude extract of *B. inquinans* (isolate MSp3-1) cultured on solid Czapek medium, nor in the HPLC analysis of the crude extract of the fungal culture with addition of a mixture of $MgSO_4$, $NaNO_3$ and NaCl, perhaps due to their low amount and/or low UV absorption. However, compounds **6**, **7** and **16** were only obtained from chromatographic workup on OSMAC extract, while compound **4** and xenofuranone B (**17**) were only afforded from extract of fungal culture without salt mixture. Compound **5** was isolated from both extracts.

(A)

(B)

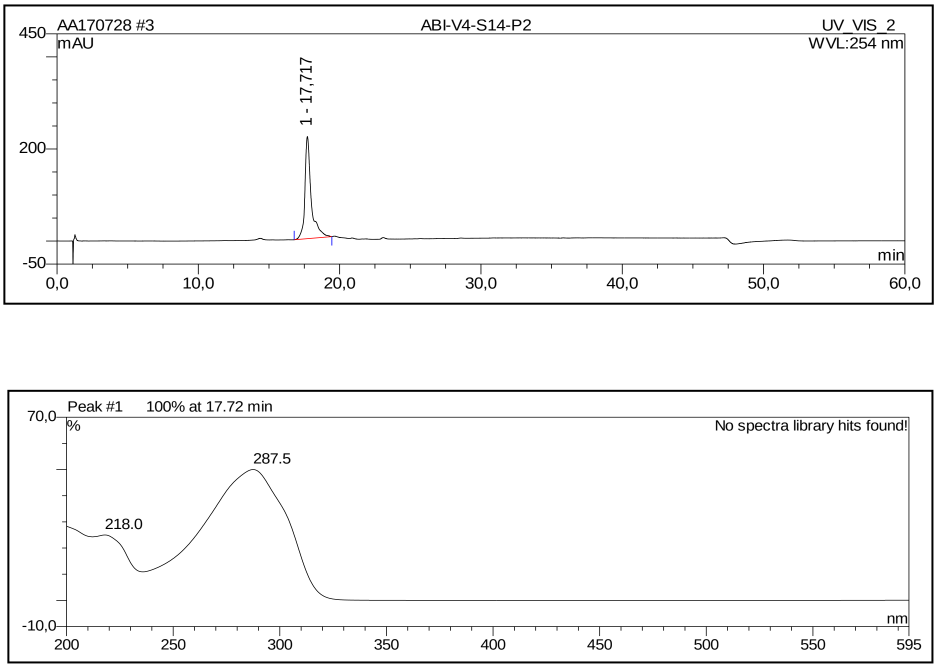

Figure S2. HPLC chromatogram (A) and UV spectrum (B) of compound **1**.

Acquisition Parameter

Source Type	ESI	Ion Polarity	Positive	Set Nebulizer	0.3 Bar
Focus	Not active	Set Capillary	4000 V	Set Dry Heater	180 °C
Scan Begin	50 m/z	Set End Plate Offset	-500 V	Set Dry Gas	4.0 l/min
Scan End	1500 m/z	Set Collision Cell RF	600.0 Vpp	Set Divert Valve	Source

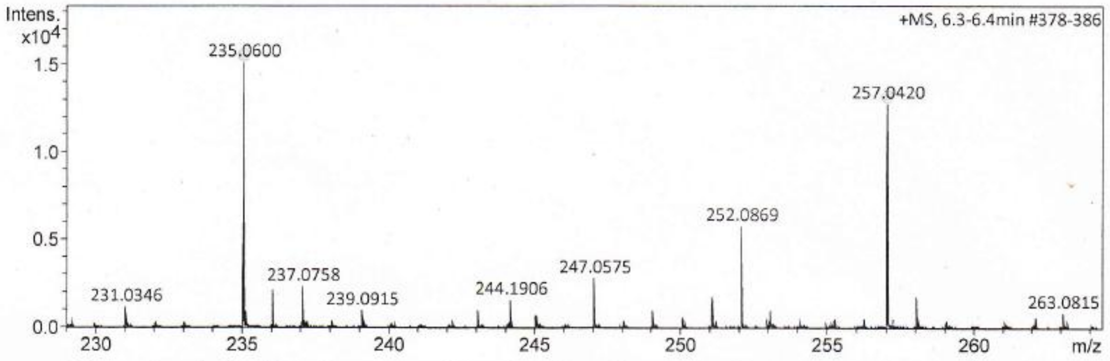

Meas. m/z	#	Ion Formula	m/z	err [ppm]	mSigma	# mSigma	Score	rdb	e⁻ Conf	N-Rule
235.0600	1	$C_{12}H_{11}O_5$	235.0601	0.3	11.8	1	100.00	7.5	even	ok
257.0420	1	$C_{12}H_{10}NaO_5$	257.0420	0.0	8.3	1	100.00	7.5	even	ok

Figure S3. HRESIMS spectrum of compound **1**.

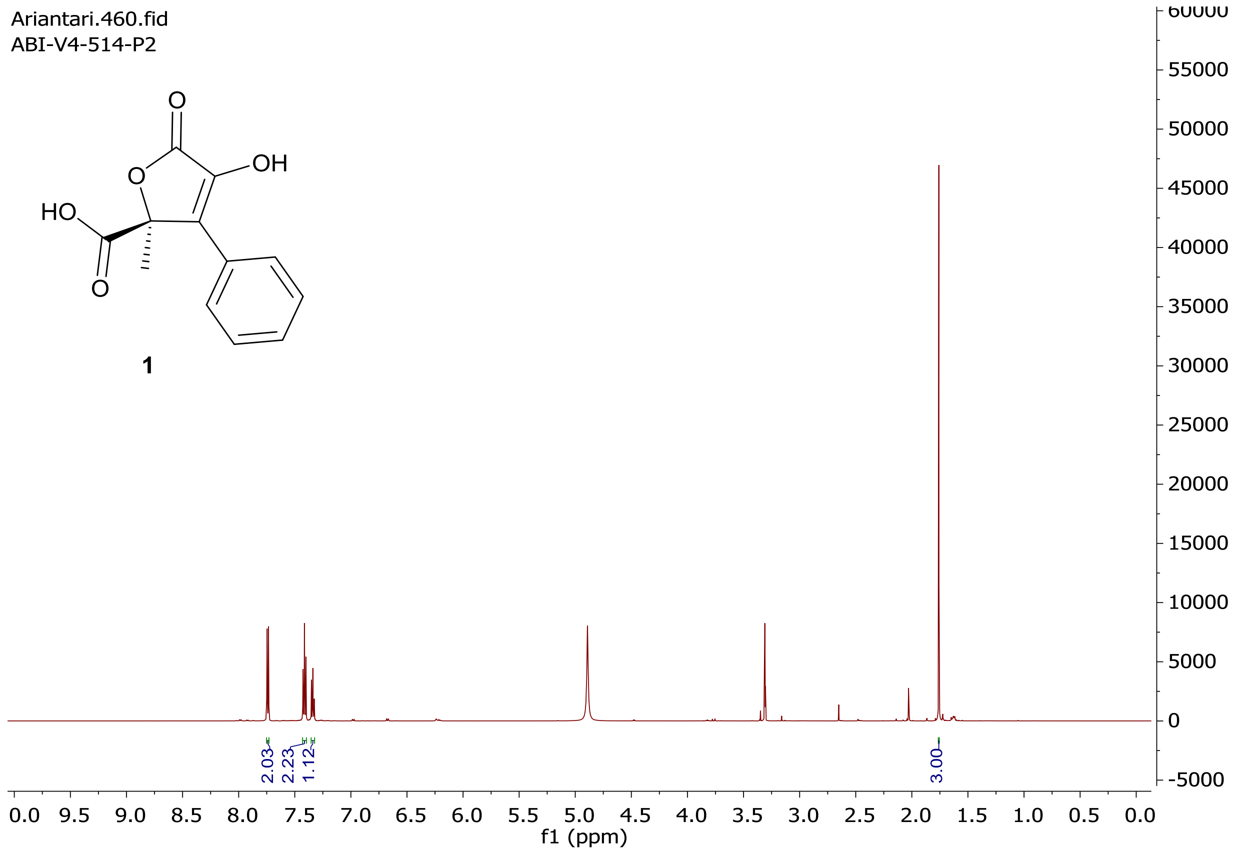

Figure S4. ^{1}H NMR (600 MHz, MeOH-d_4) spectrum of compound **1**.

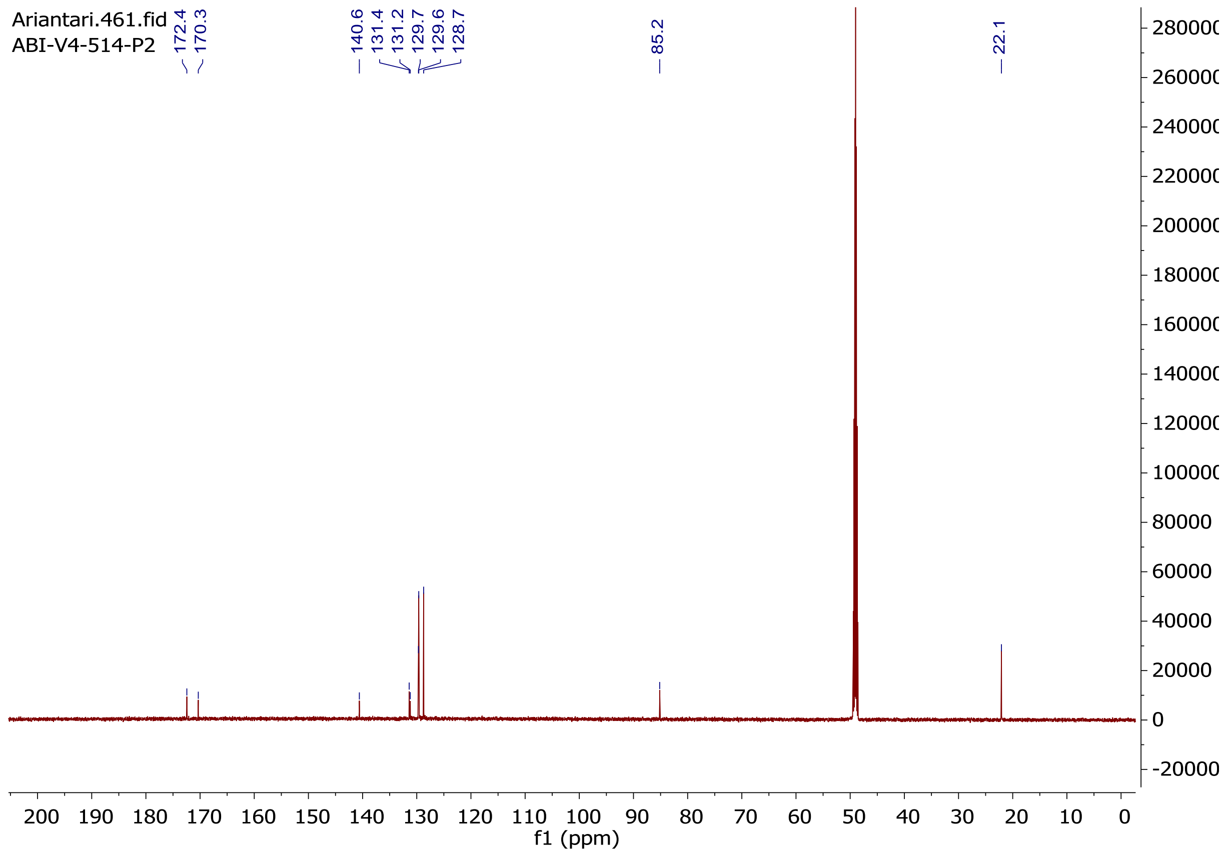

Figure S5. ^{13}C NMR (150 MHz, MeOH-d_4) spectrum of compound **1**.

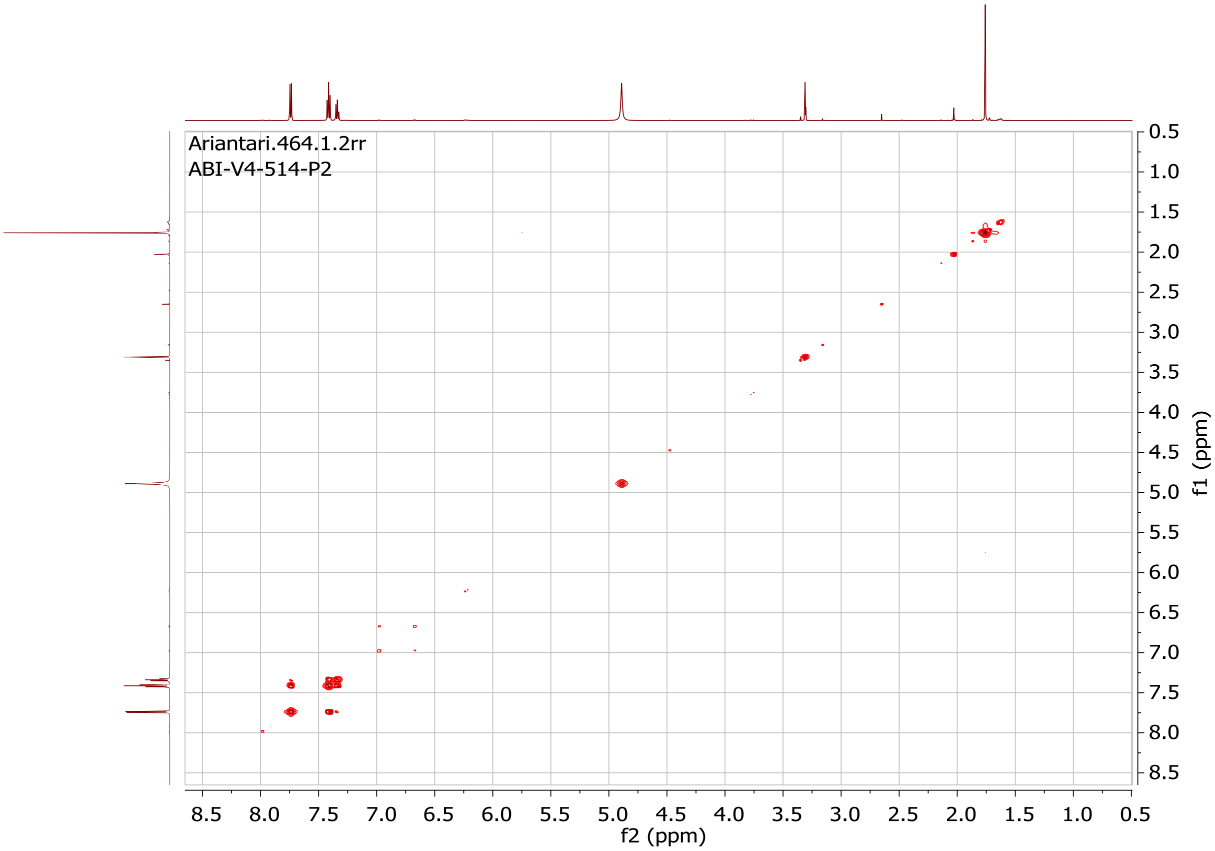

Figure S6. ^{1}H-^{1}H COSY (600 MHz, MeOH-d_4) spectrum of compound **1**.

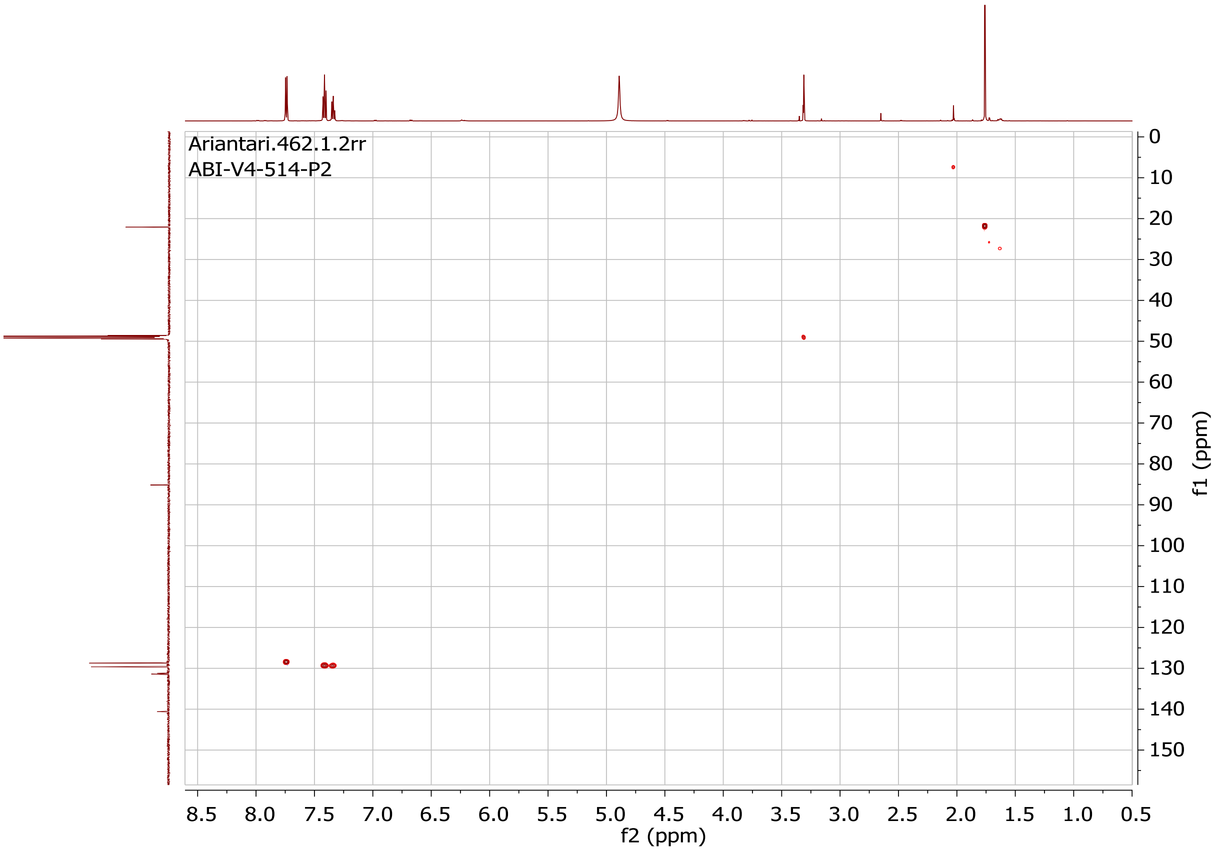

Figure S7. HSQC (600 and 150 MHz, MeOH-d_4) spectrum of compound **1**.

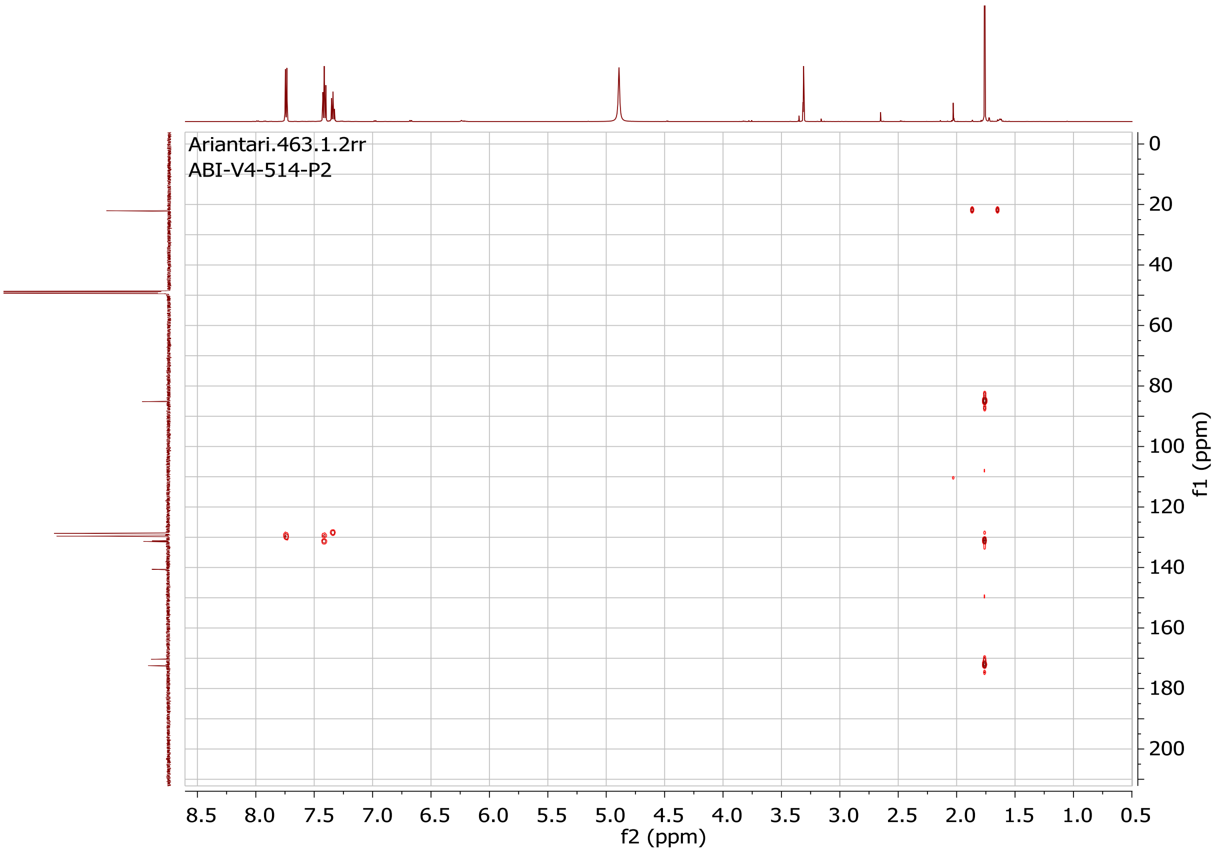

Figure S8. HMBC (600 and 150 MHz, MeOH-d_4) spectrum of compound **1**.

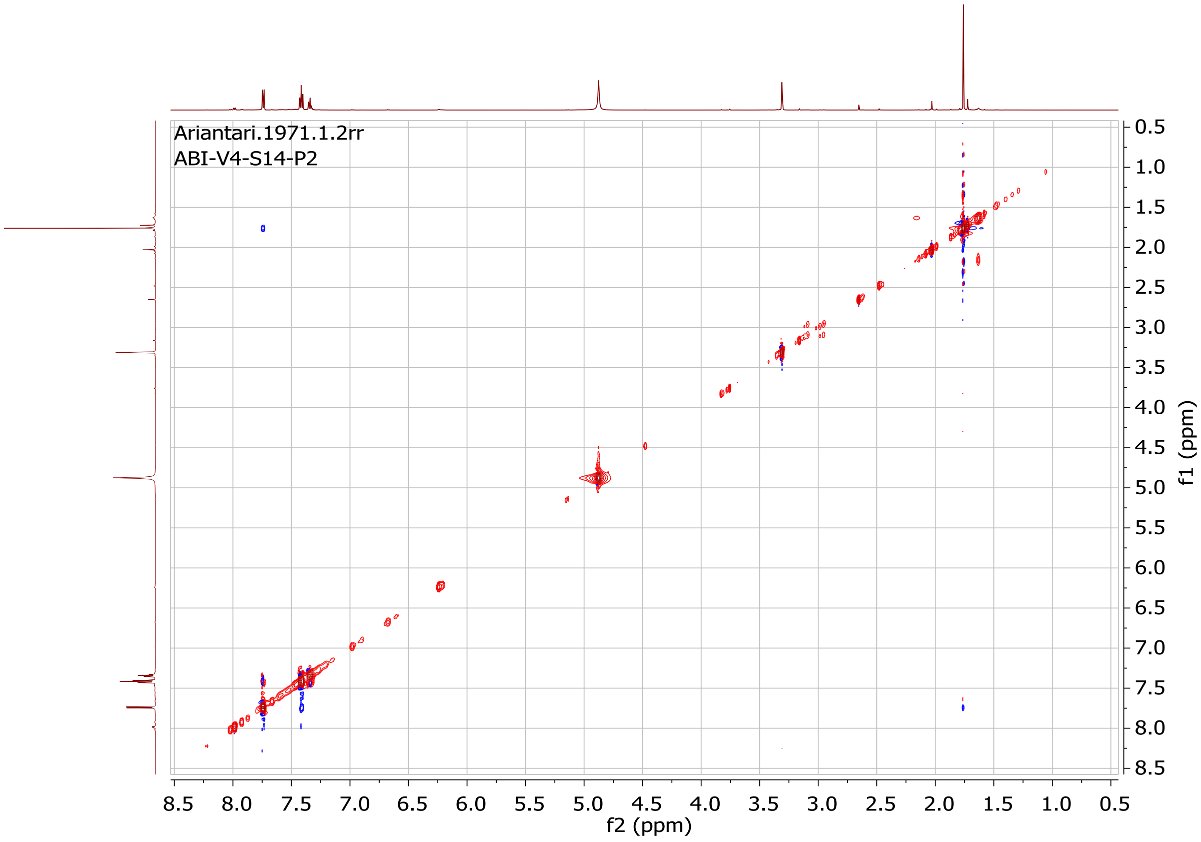

Figure S9. NOESY (600 MHz, MeOH-d_4) spectrum of compound **1**.

(A)

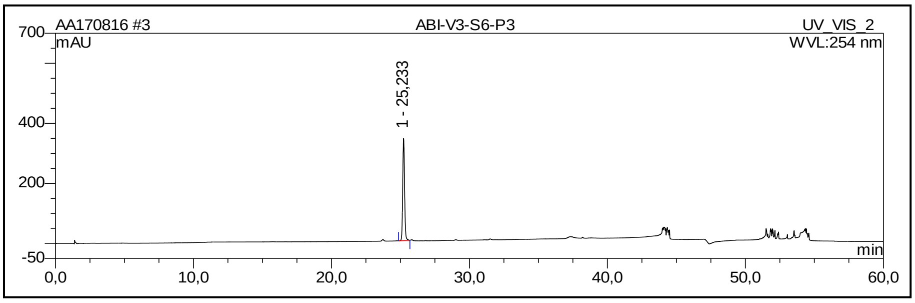

(B)

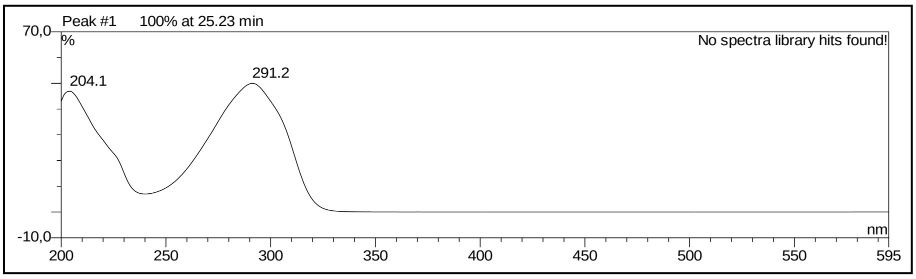

Figure S10. HPLC chromatogram (A) and UV spectrum (B) of compound **2**.

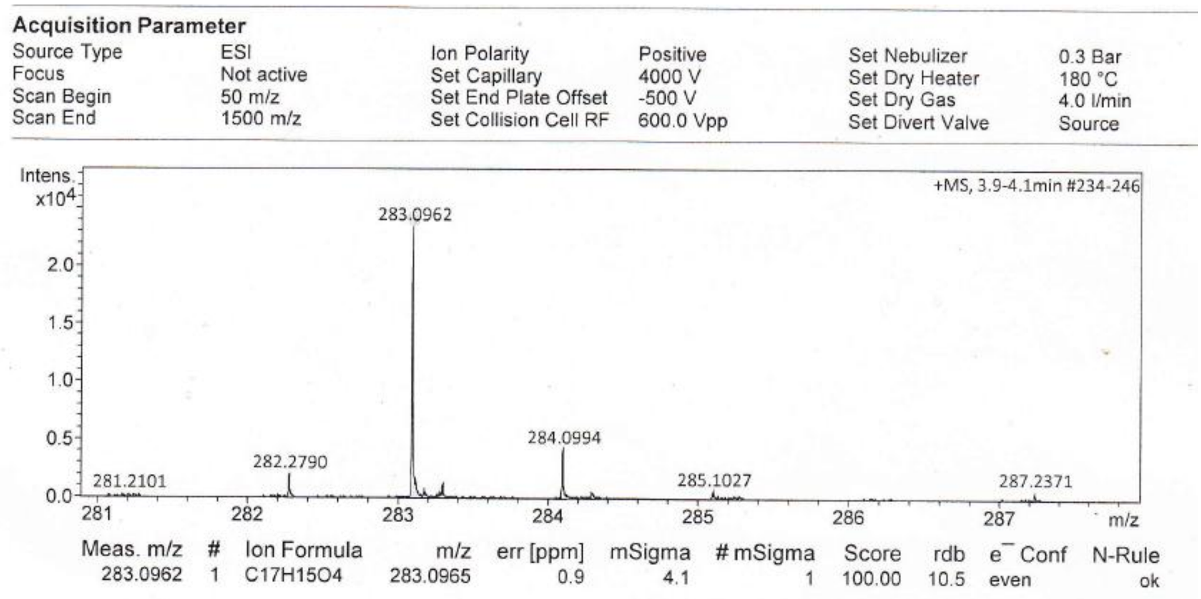

Figure S11. HRESIMS spectrum of compound **2**.

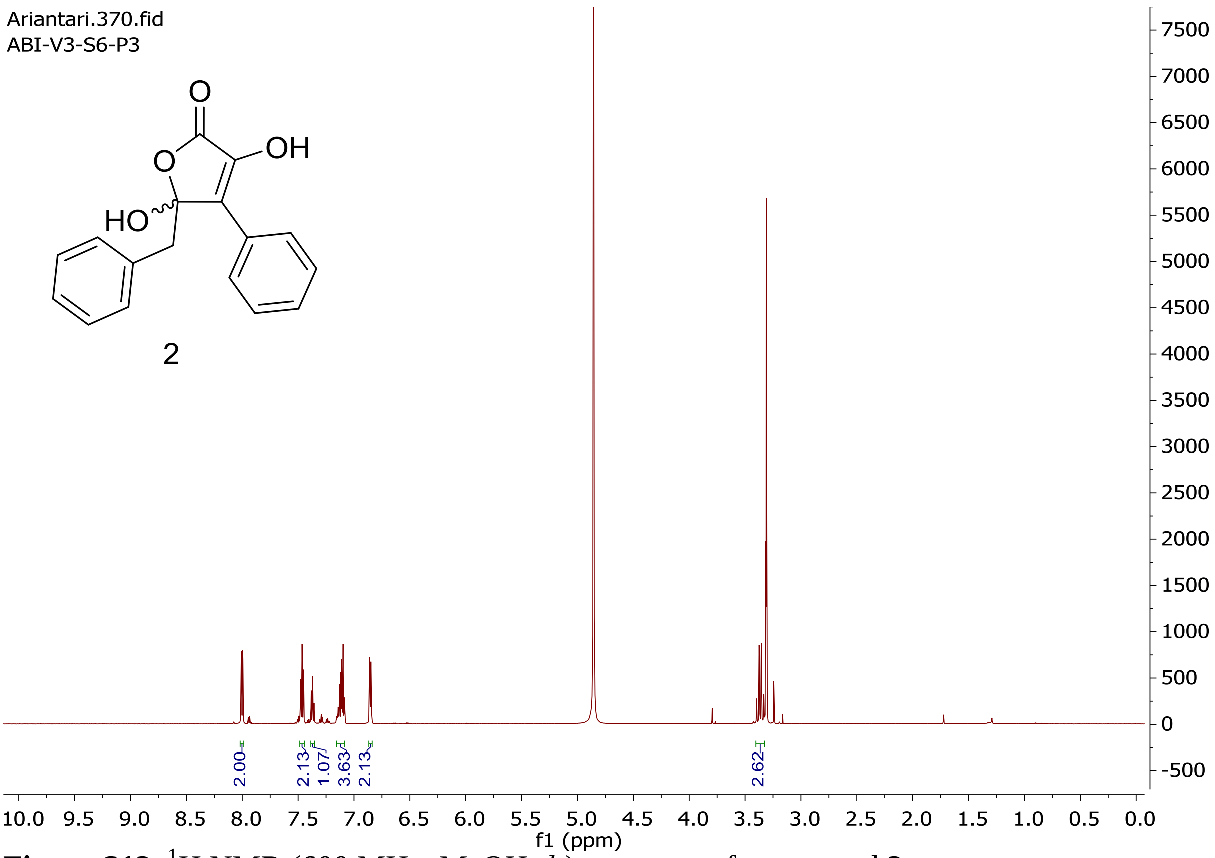

Figure S12. ^{1}H NMR (600 MHz, MeOH-d_4) spectrum of compound **2**.

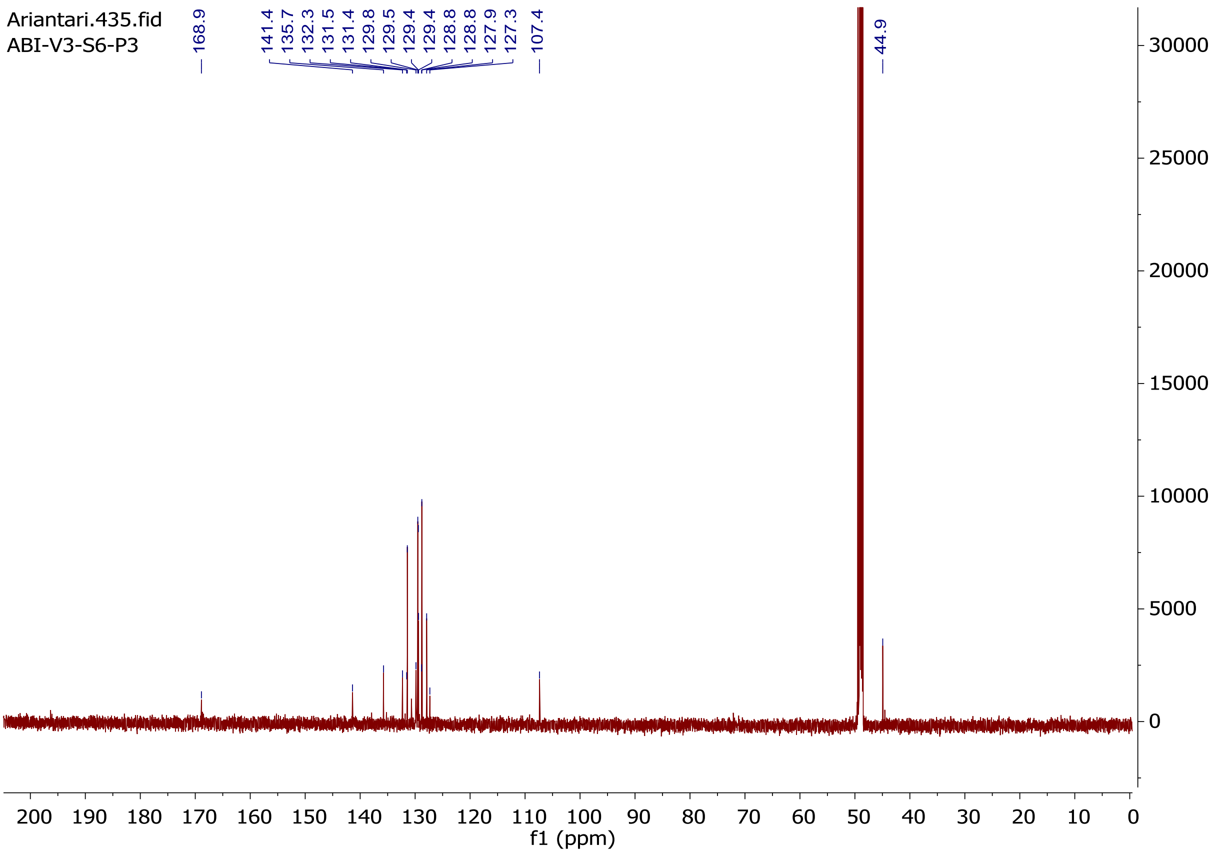

Figure S13. ^{13}C NMR (150 MHz, MeOH-d_4) spectrum of compound **2**.

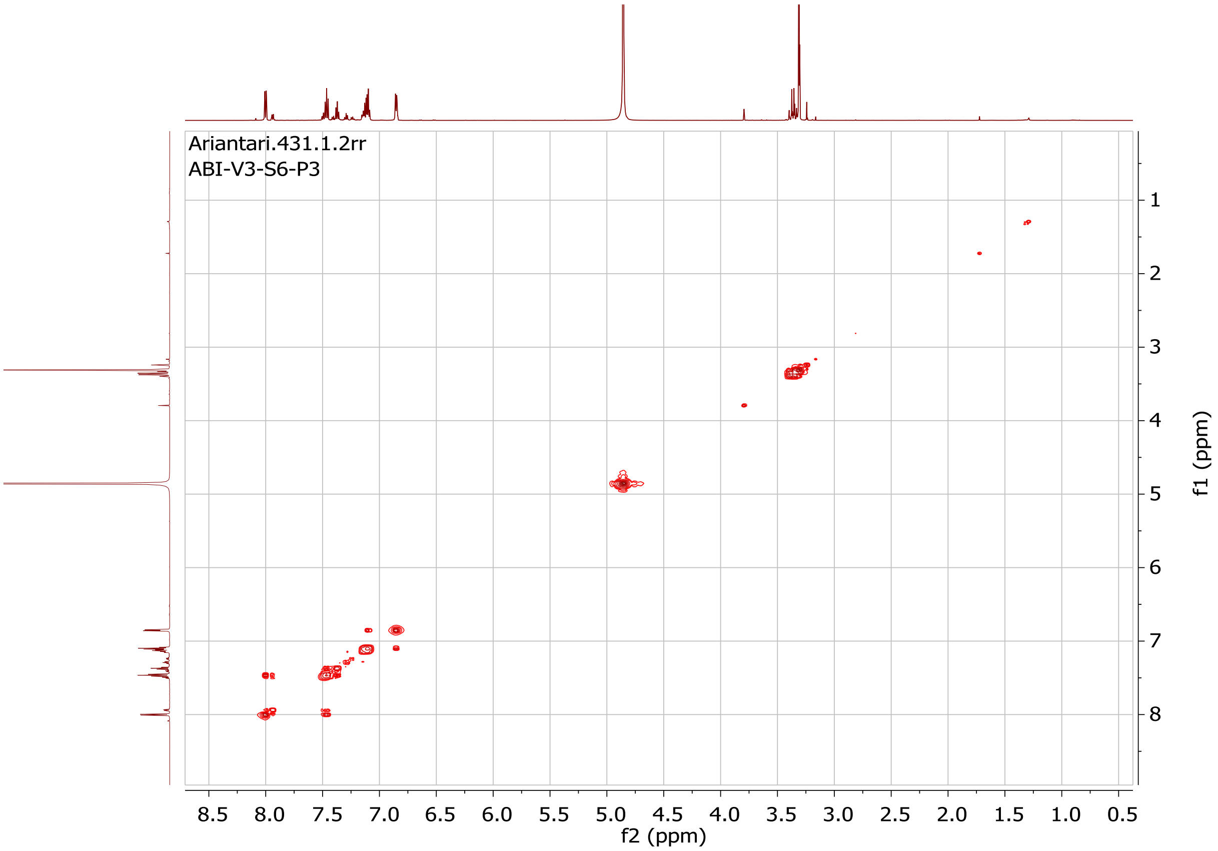

Figure S14. ^1H-^1H COSY (600 MHz, MeOH-d_4) spectrum of compound **2**.

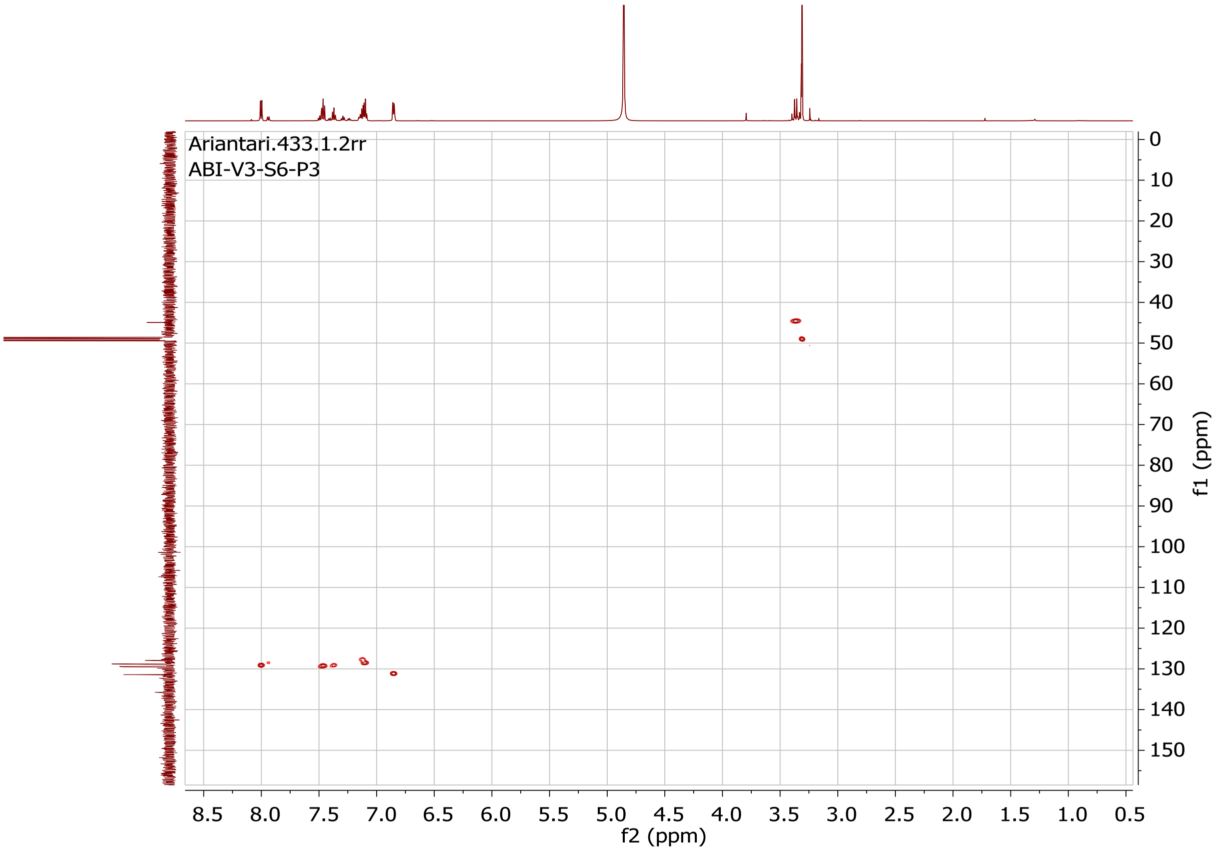

Figure S15. HSQC (600 and 150 MHz, MeOH-d_4) spectrum of compound **2**.

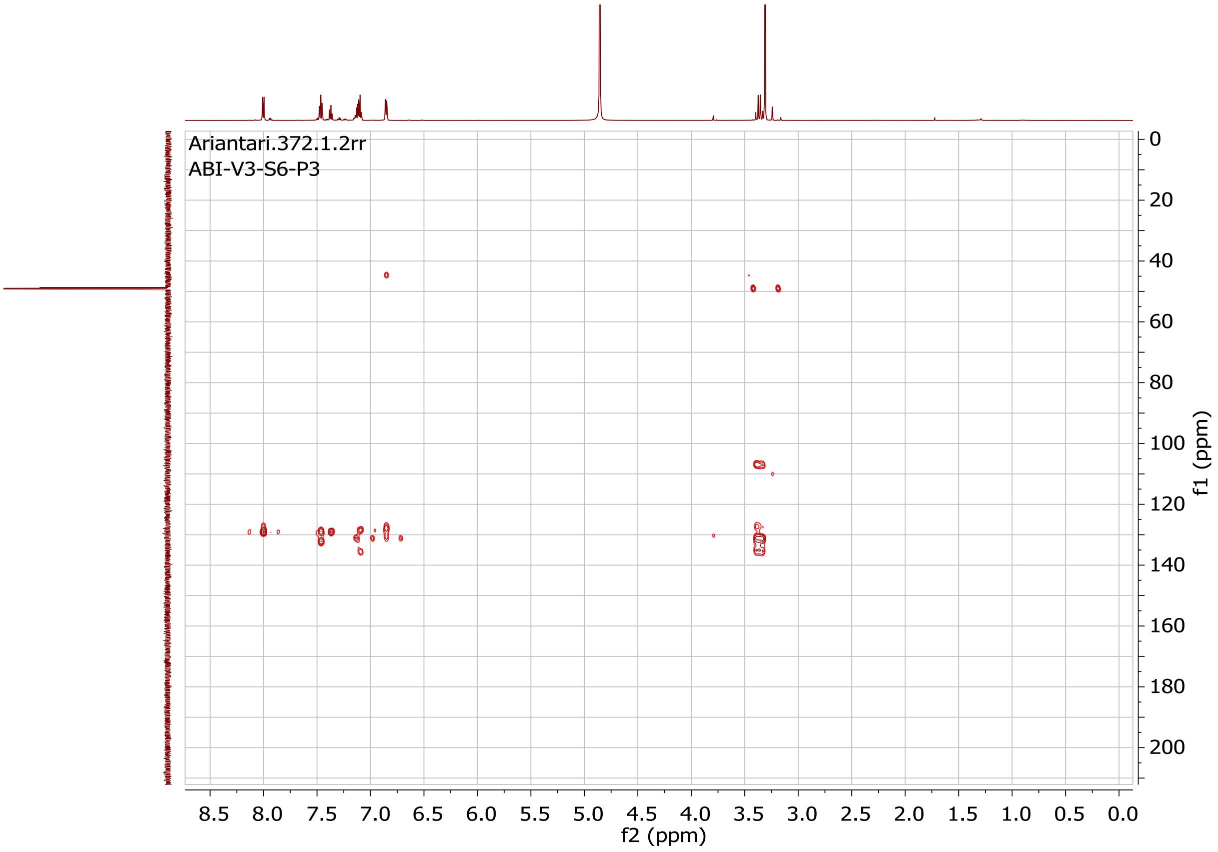

Figure S16. HMBC (600 and 150 MHz, MeOH-d_4) spectrum of compound **2**.

(A)

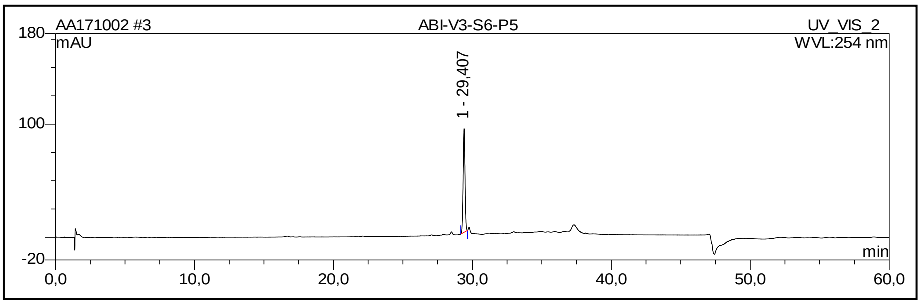

(B)

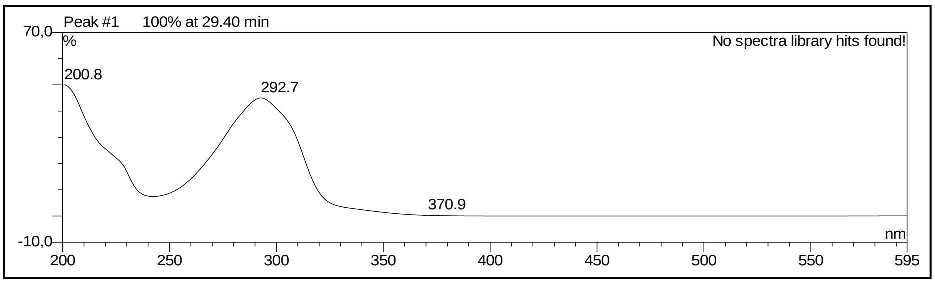

Figure S17. HPLC chromatogram (A) and UV spectrum (B) of compound **3**.

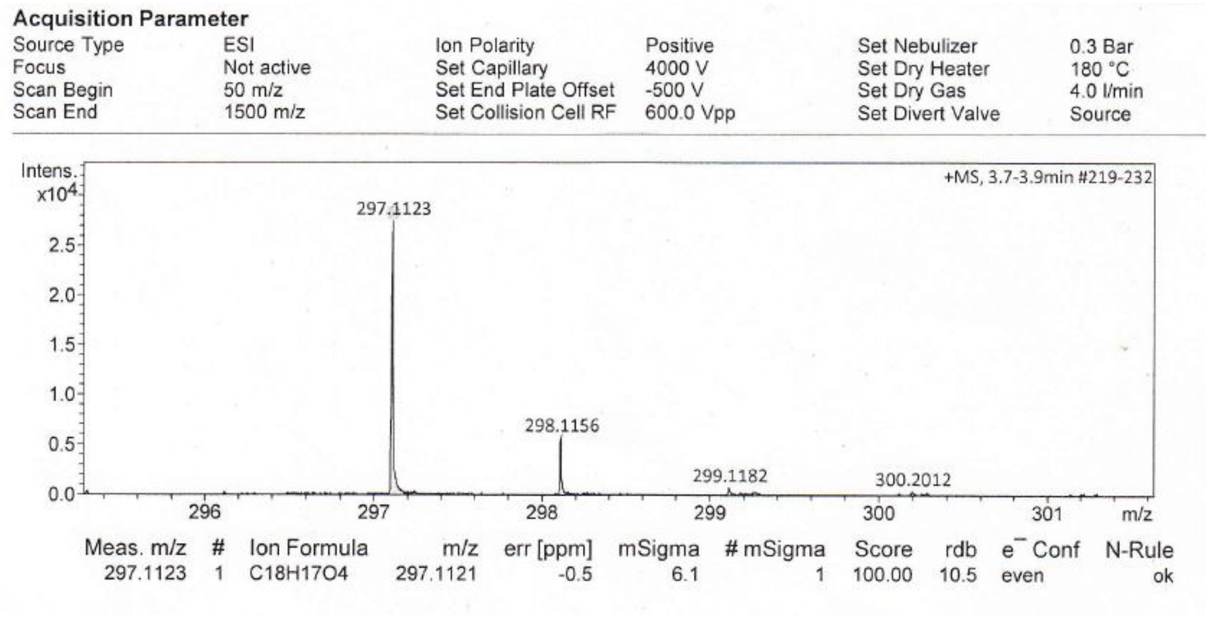

Figure S18. HRESIMS spectrum of compound **3**.

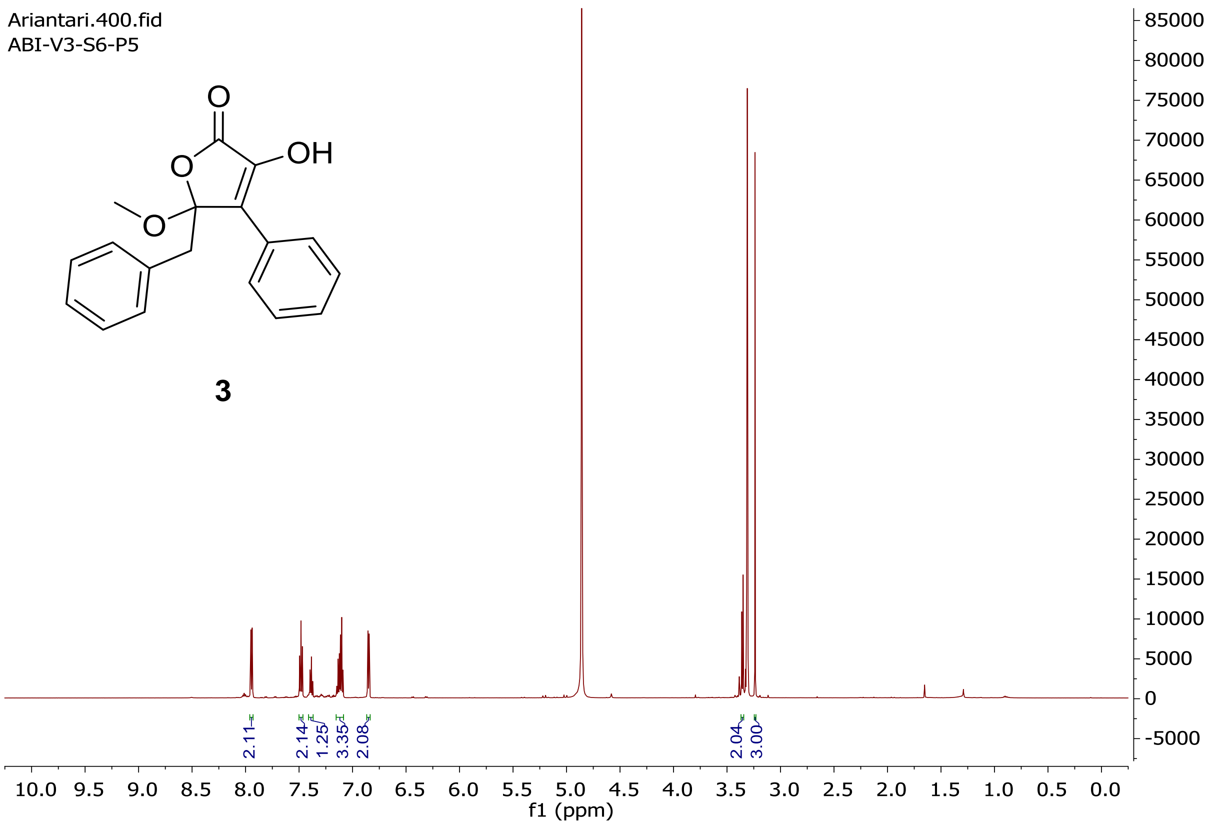

Figure S19. ^{1}H NMR (600 MHz, MeOH-d_4) spectrum of compound **3**.

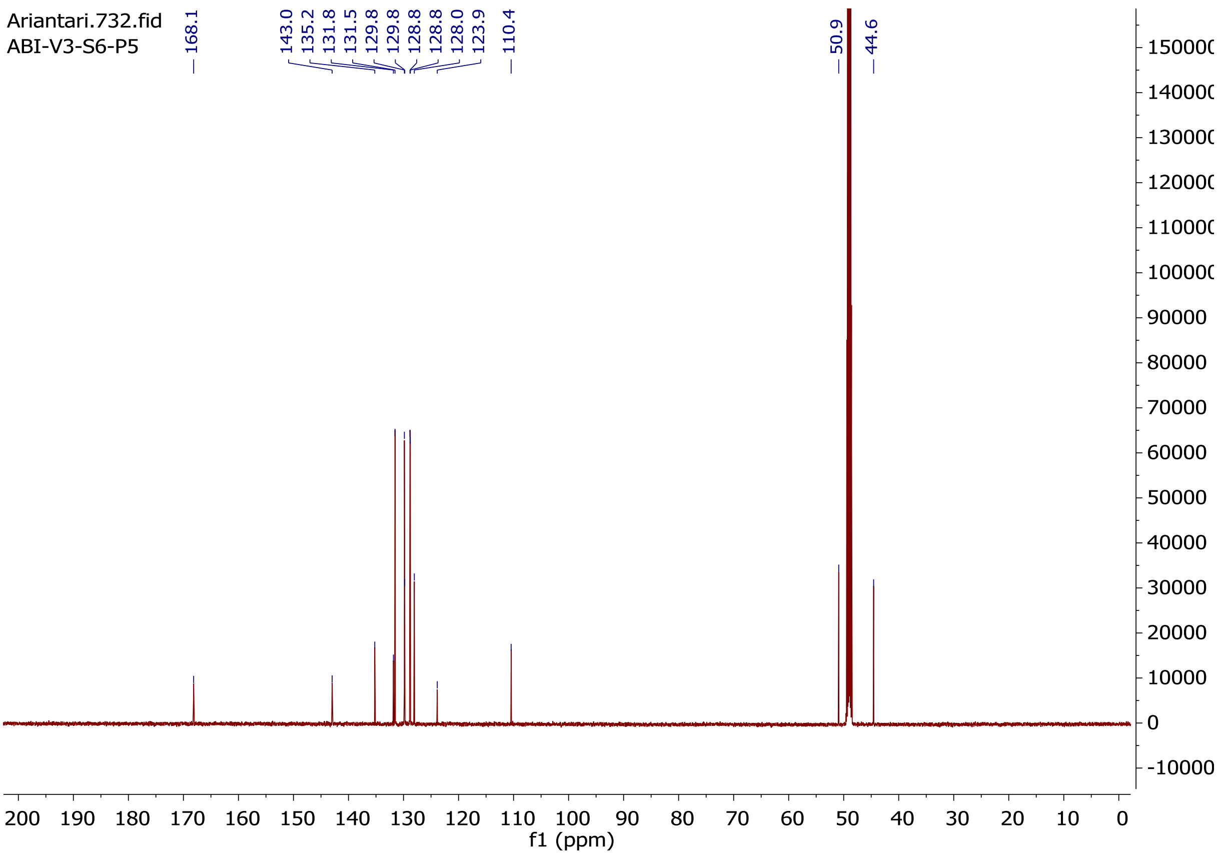

Figure S20. ^{13}C NMR (150 MHz, MeOH-d_4) spectrum of compound **3**.

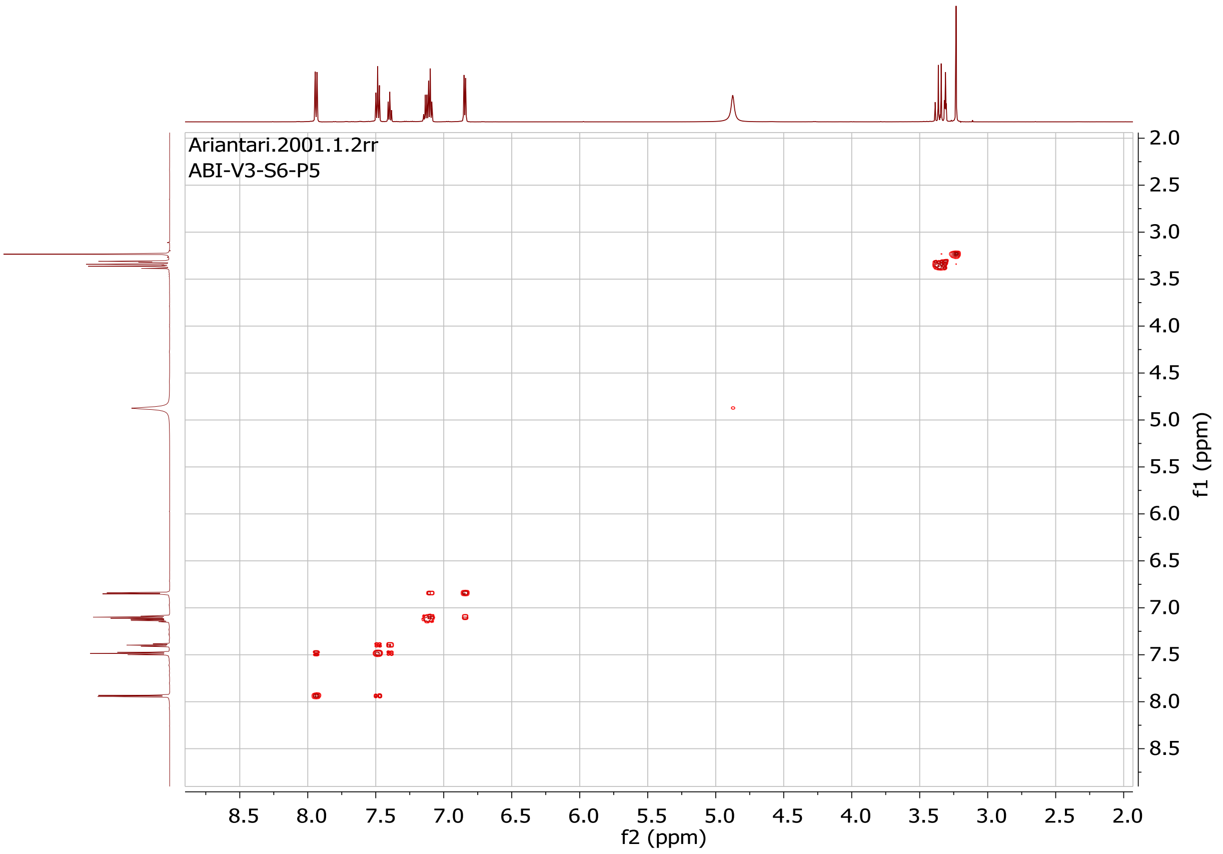

Figure S21. ^{1}H-^{1}H COSY (600 MHz, MeOH-d_4) spectrum of compound **3**.

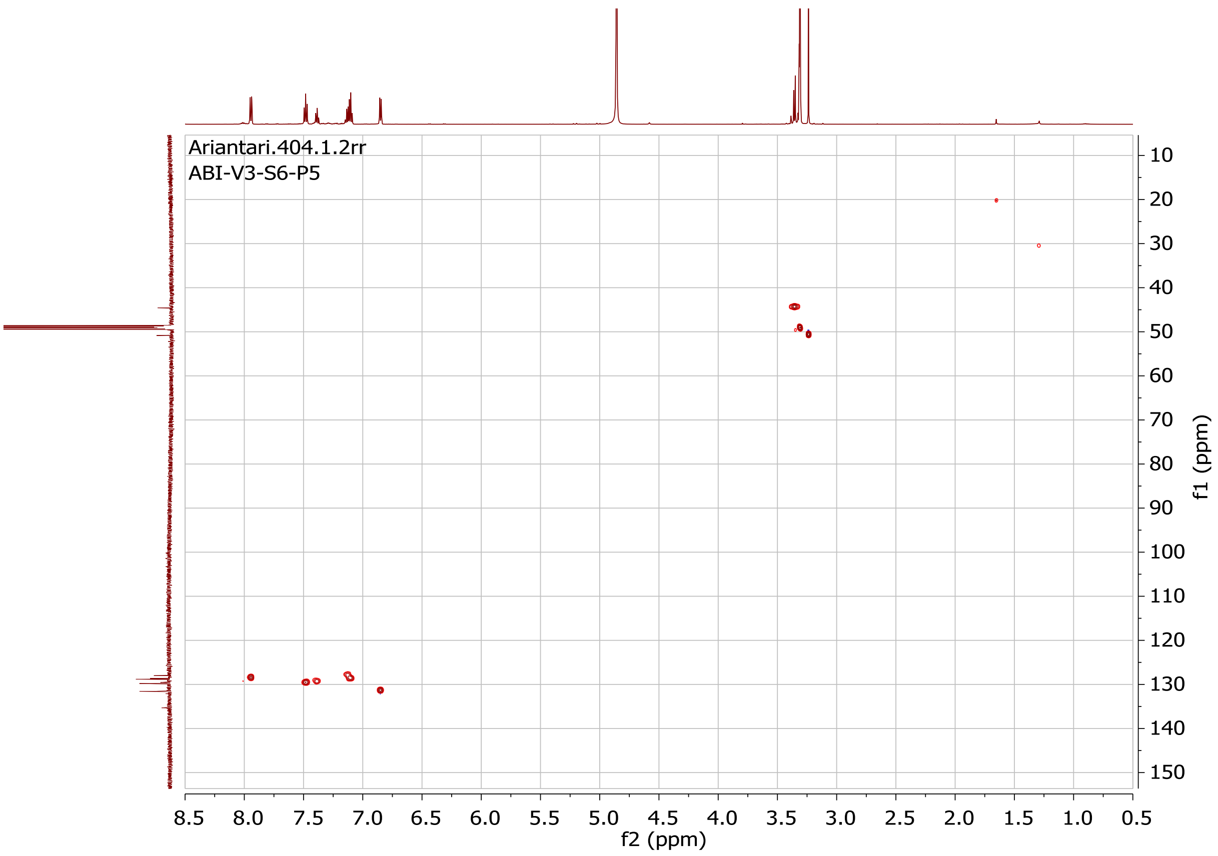

Figure S22. HSQC (600 and 150 MHz, MeOH-d_4) spectrum of compound **3**.

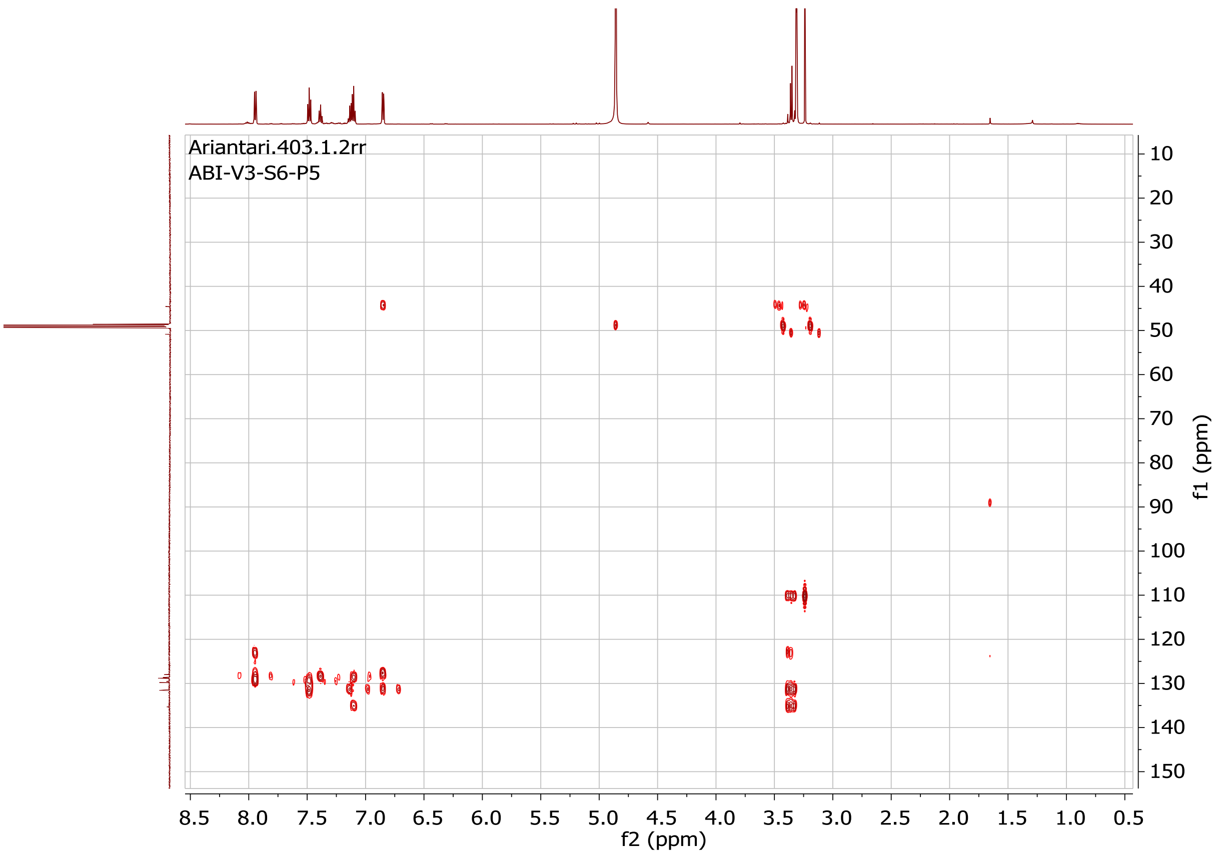

Figure S23. HMBC (600 and 150 MHz, MeOH-d_4) spectrum of compound **3**.

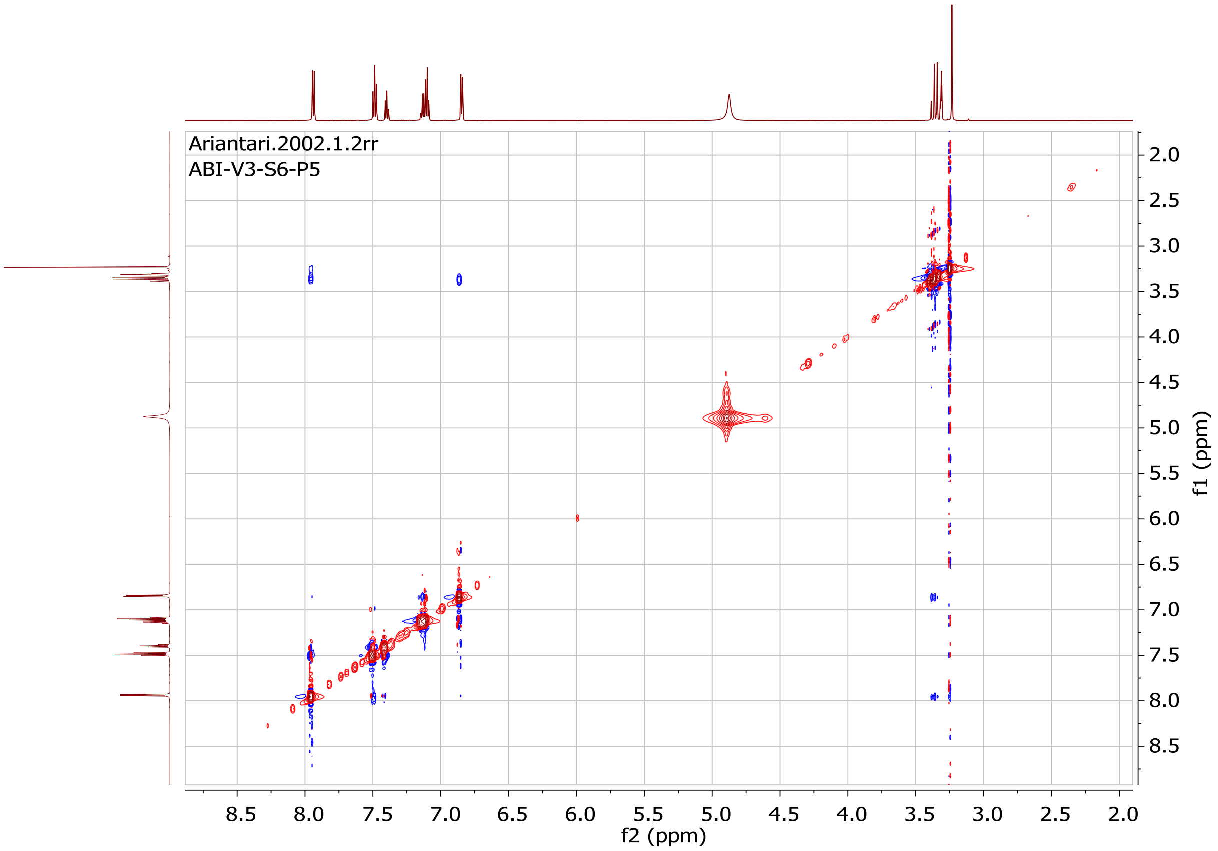

Figure S24. NOESY (600 MHz, MeOH-d_4) spectrum of compound **3**.

(A)

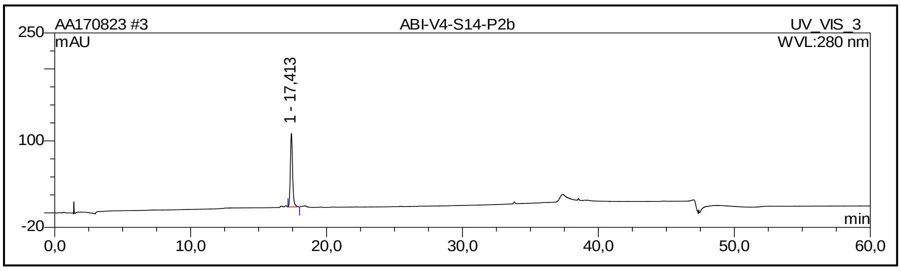

(B)

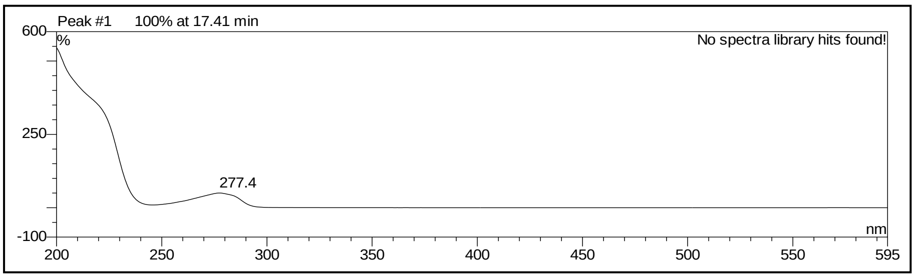

Figure S25. HPLC chromatogram (A) and UV spectrum (B) of compound **4**.

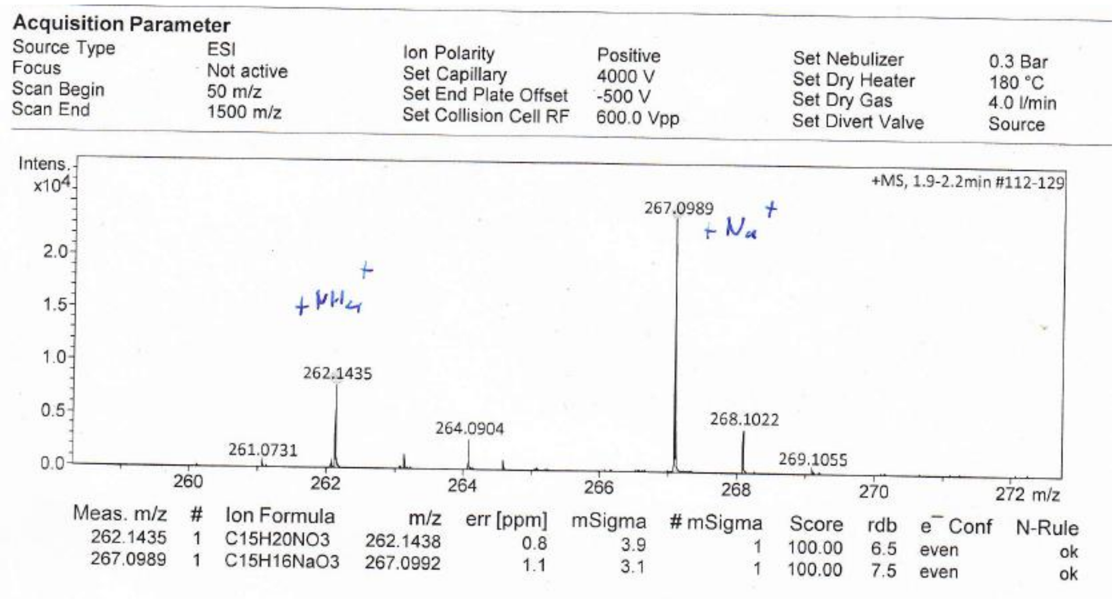

Figure S26. HRESIMS spectrum of compound **4**.

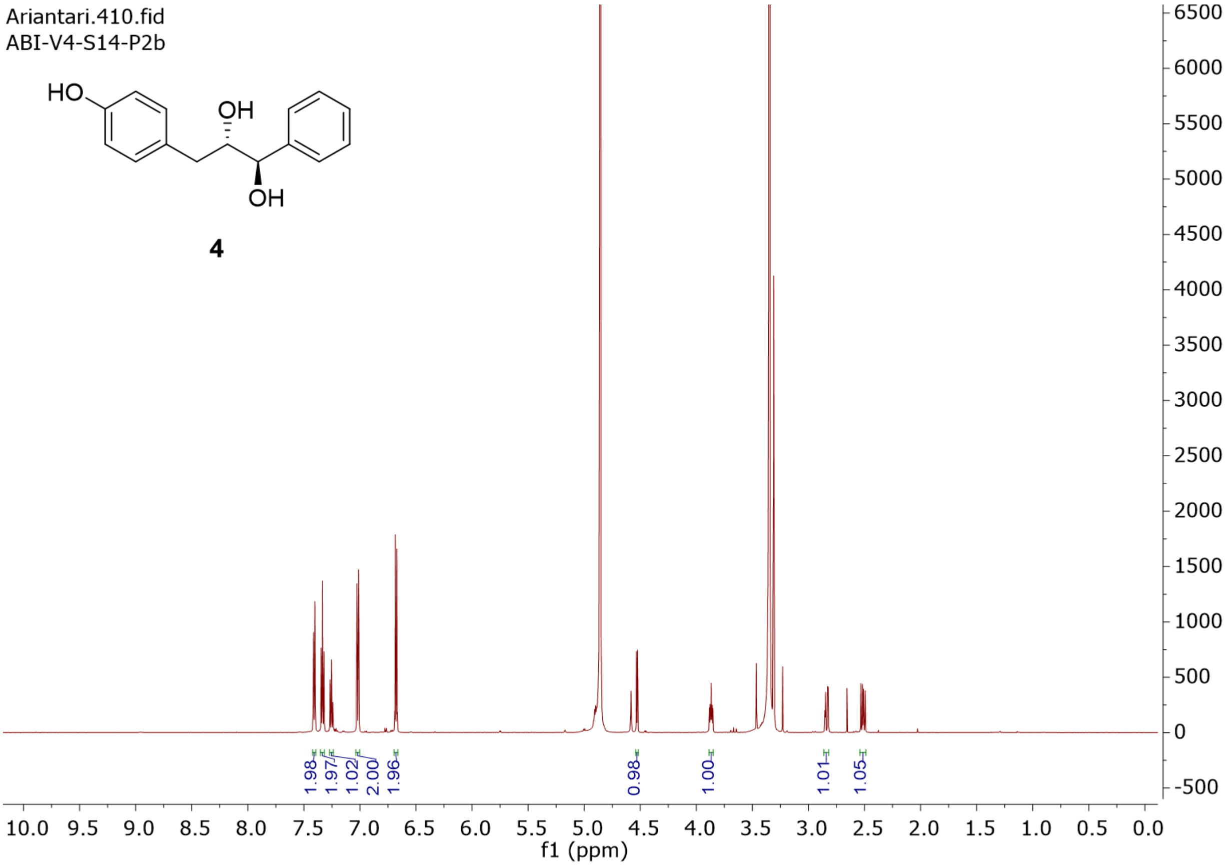

Figure S27. ^{1}H NMR (600 MHz, MeOH-d_4) spectrum of compound **4**.

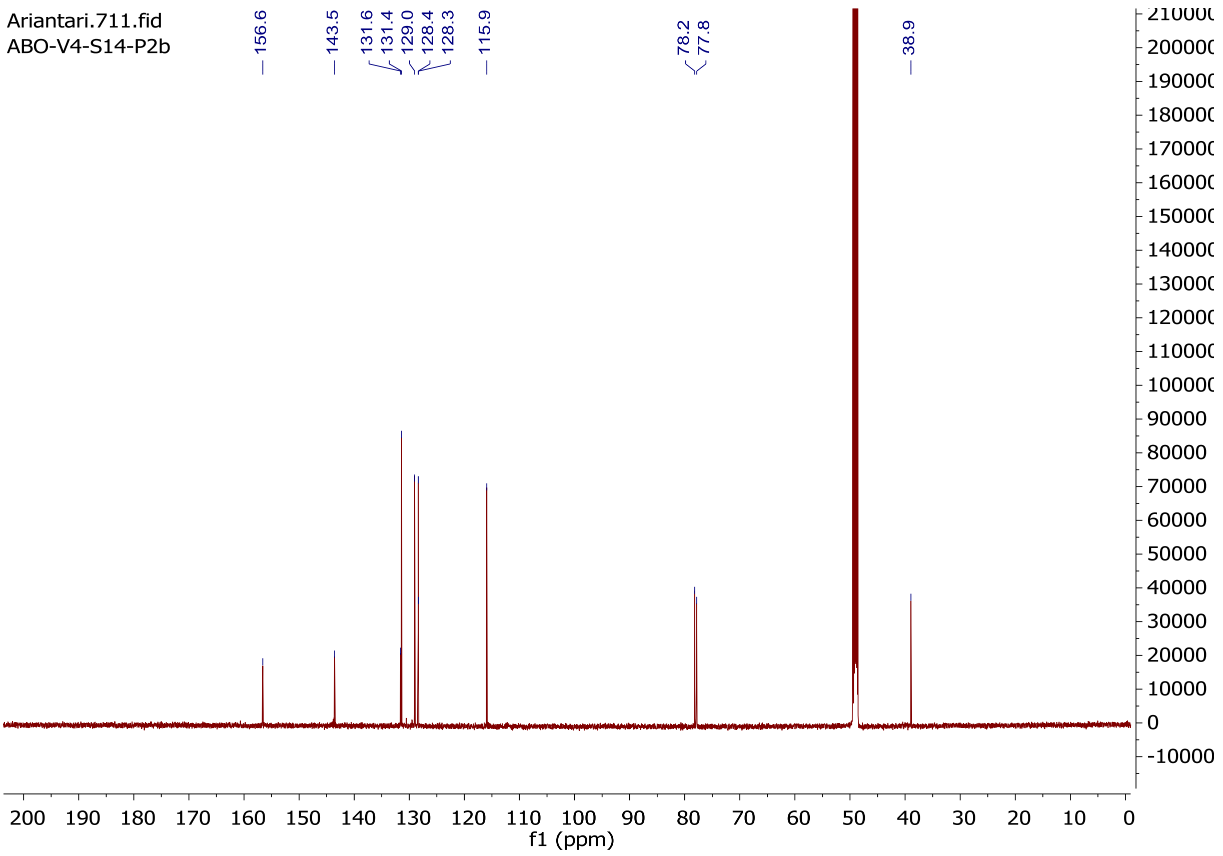

Figure S28. ^{13}C NMR (150 MHz, MeOH-d_4) spectrum of compound **4**.

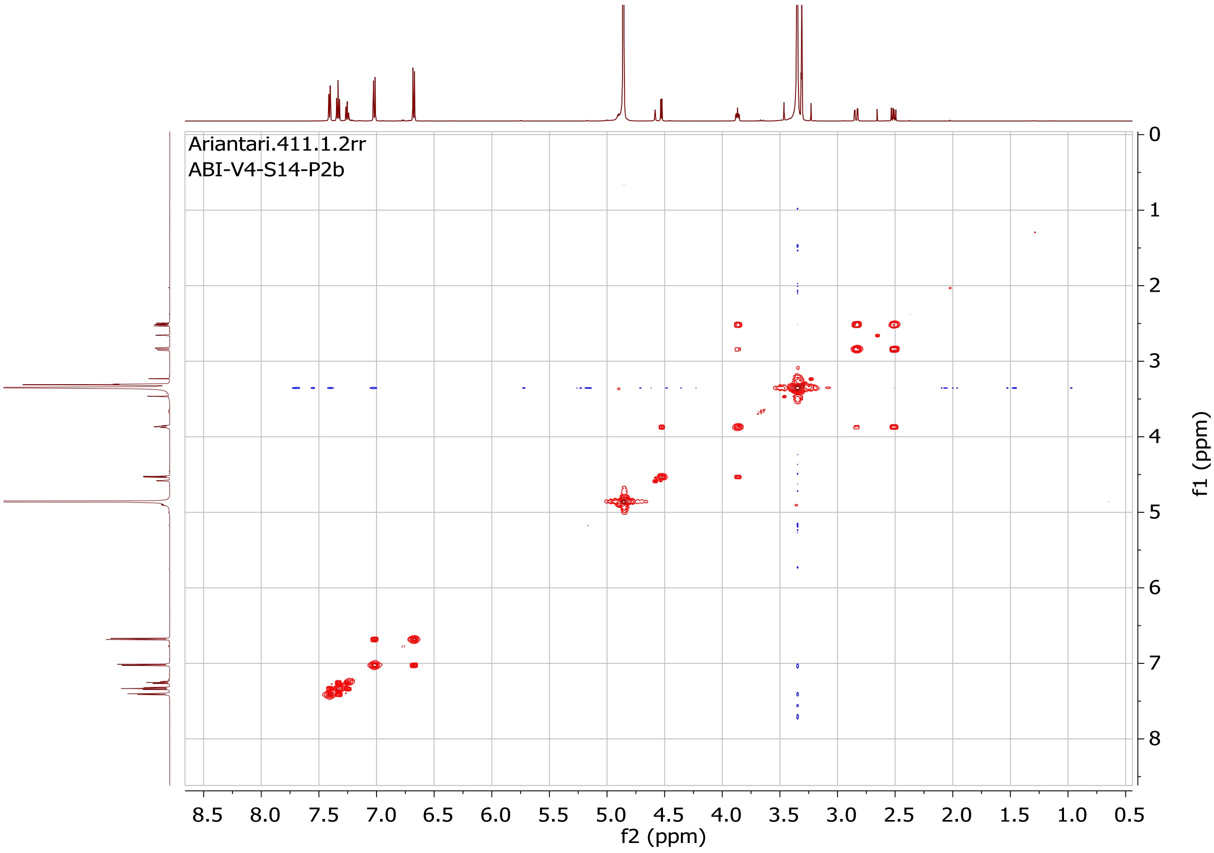

Figure S29. ^{1}H-^{1}H COSY (600 MHz, MeOH-d_4) spectrum of compound **4**.

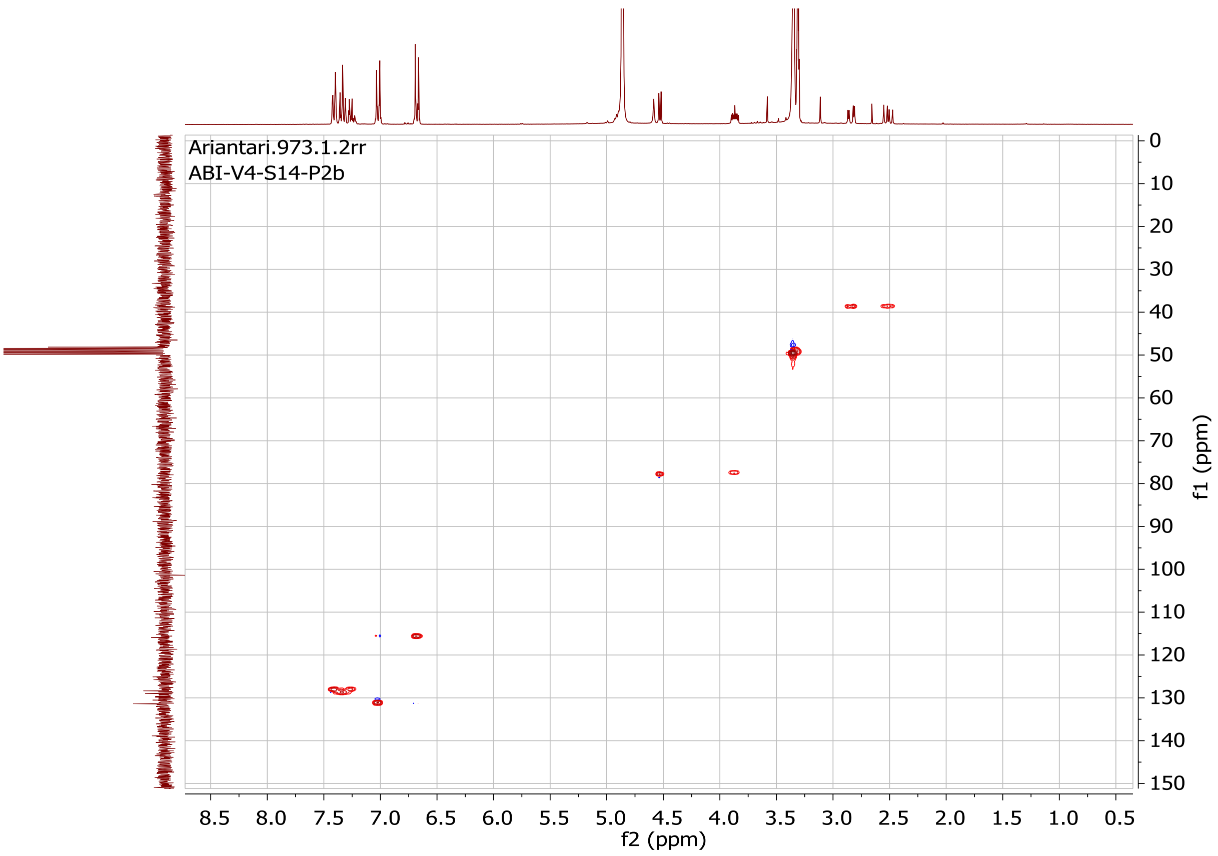

Figure S30. HSQC (300 and 75 MHz, MeOH-d_4) spectrum of compound **4**.

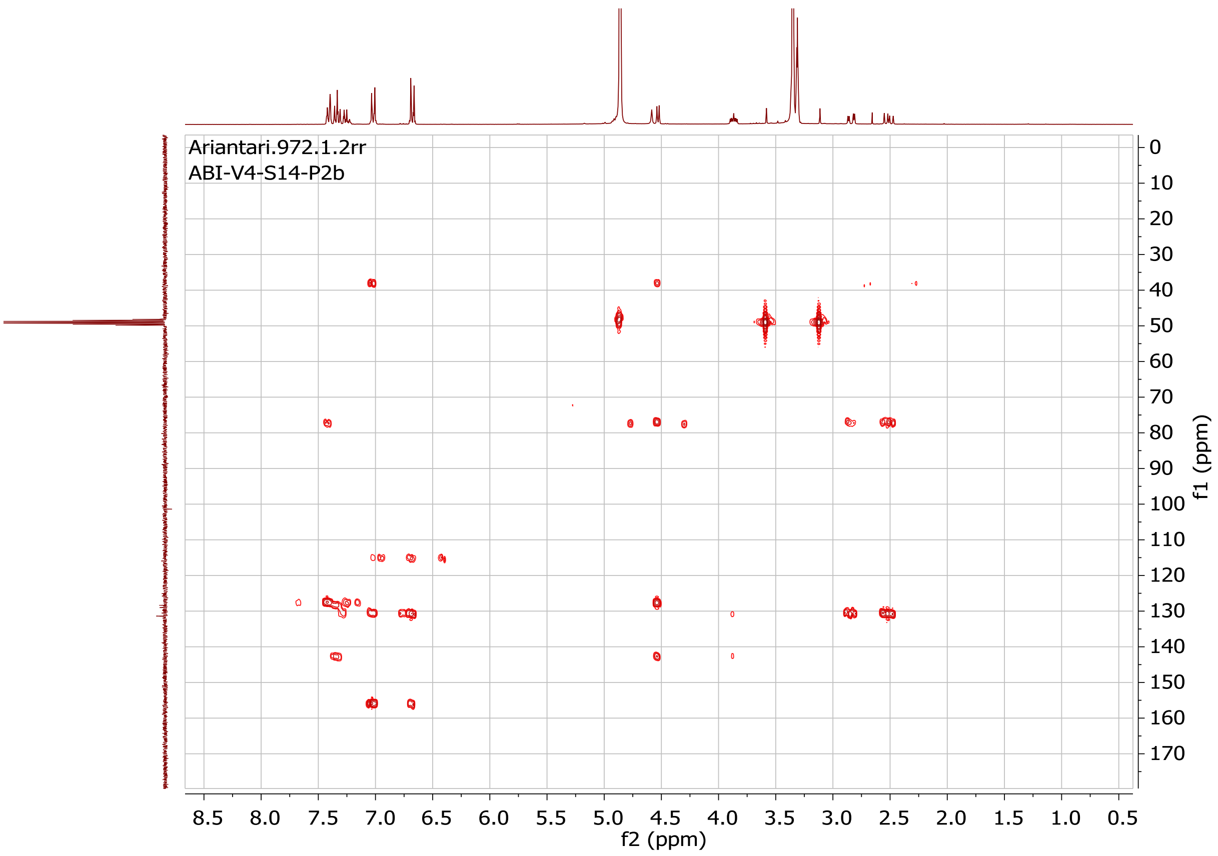

Figure S31. HMBC (300 and 75 MHz, MeOH-d_4) spectrum of compound **4**.

(A)

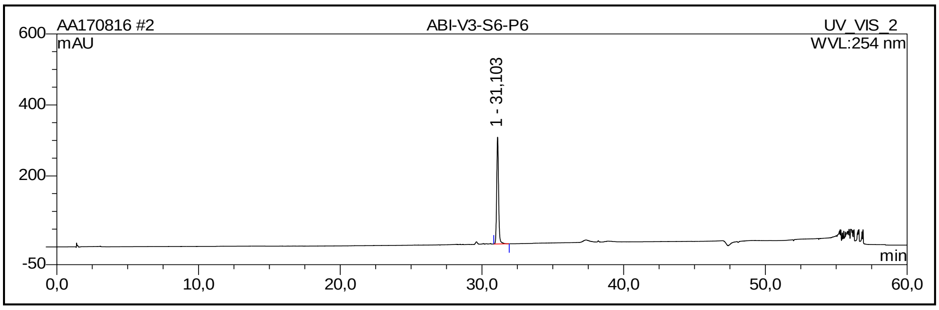

(B)

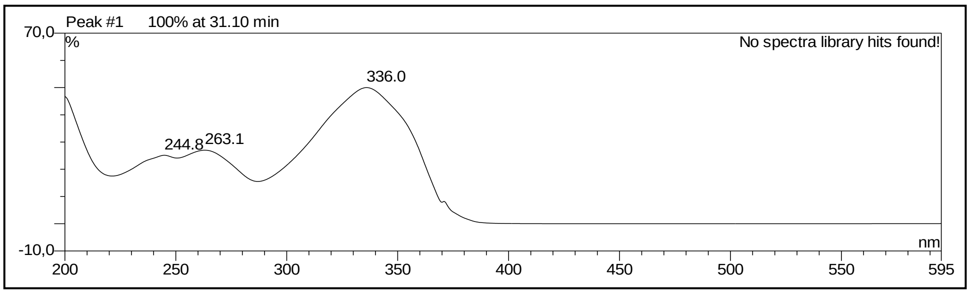

Figure S32. HPLC chromatogram (A) and UV spectrum (B) of compound **5**.

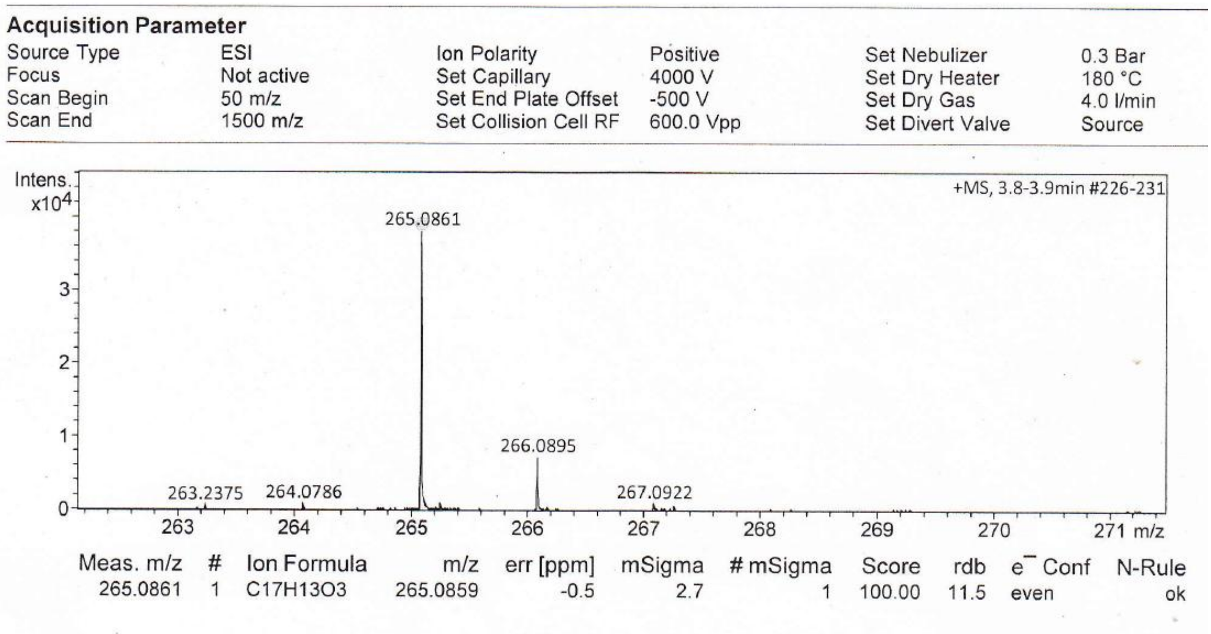

Figure S33. HRESIMS spectrum of compound **5**.

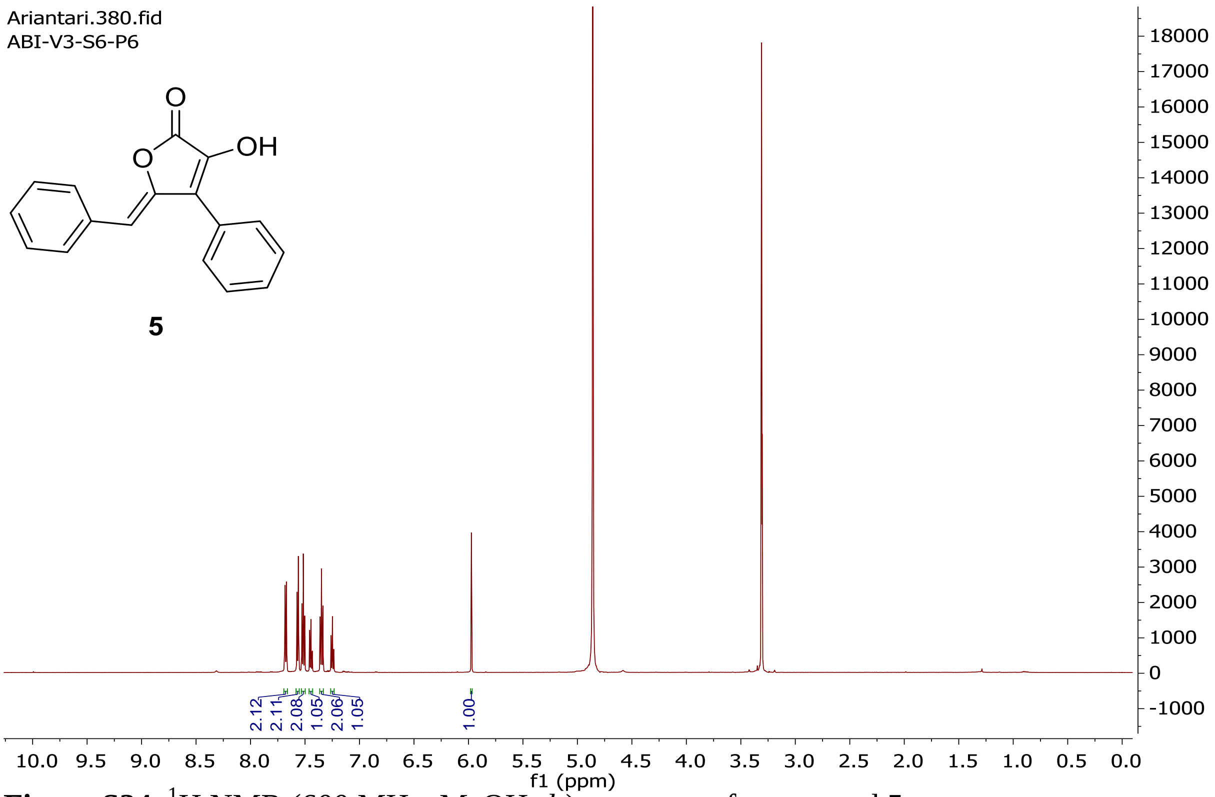

Figure S34. ^{1}H NMR (600 MHz, MeOH-d_4) spectrum of compound **5**.

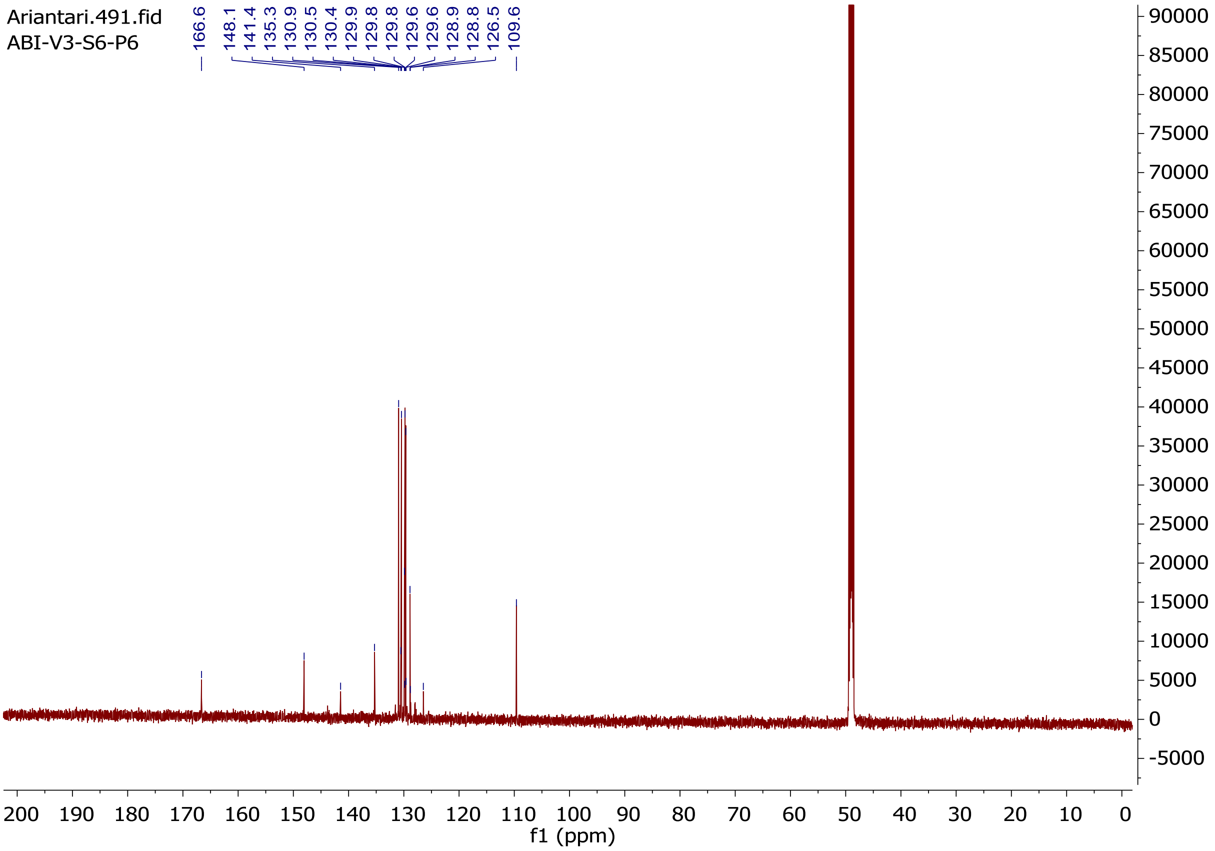

Figure S35. ^{13}C NMR (150 MHz, MeOH-d_4) spectrum of compound **5.**

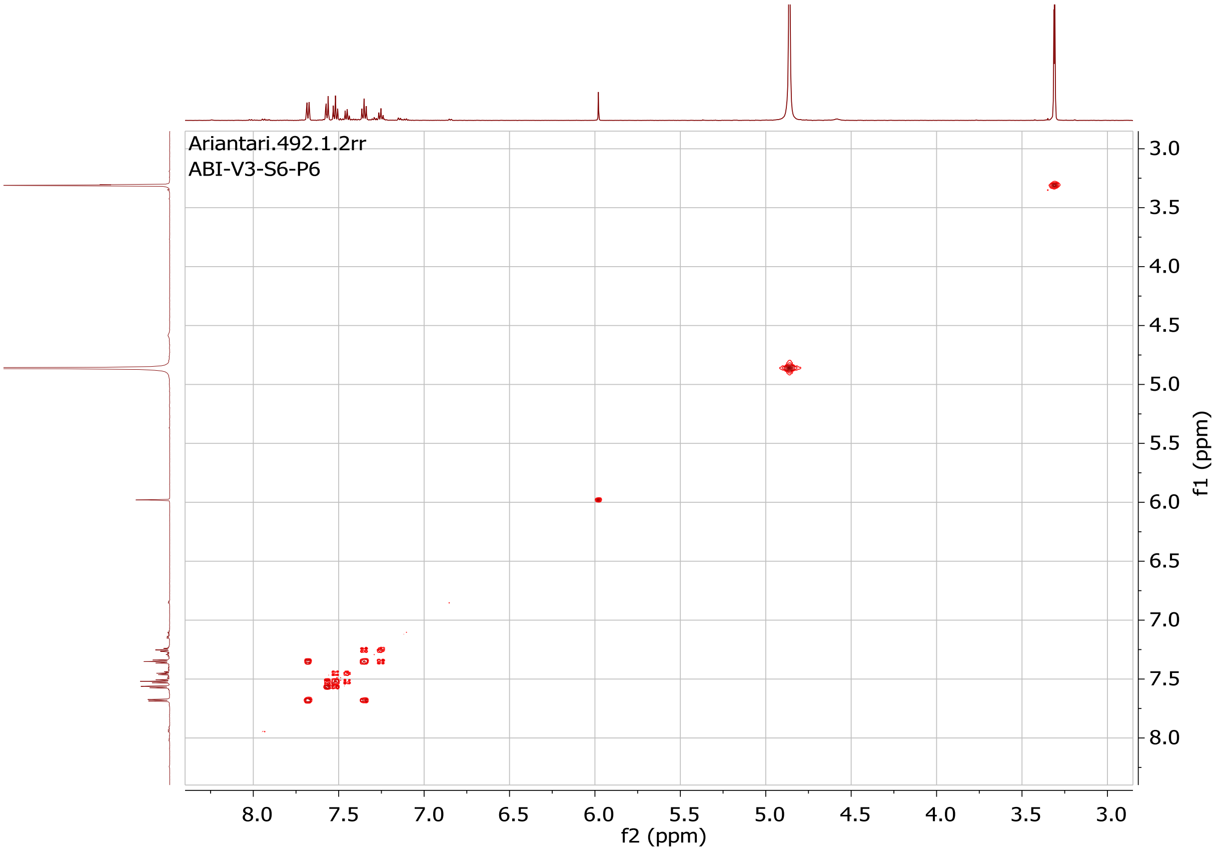

Figure S36. ^{1}H-^{1}H COSY (600 MHz, MeOH-d_4) spectrum of compound **5**.

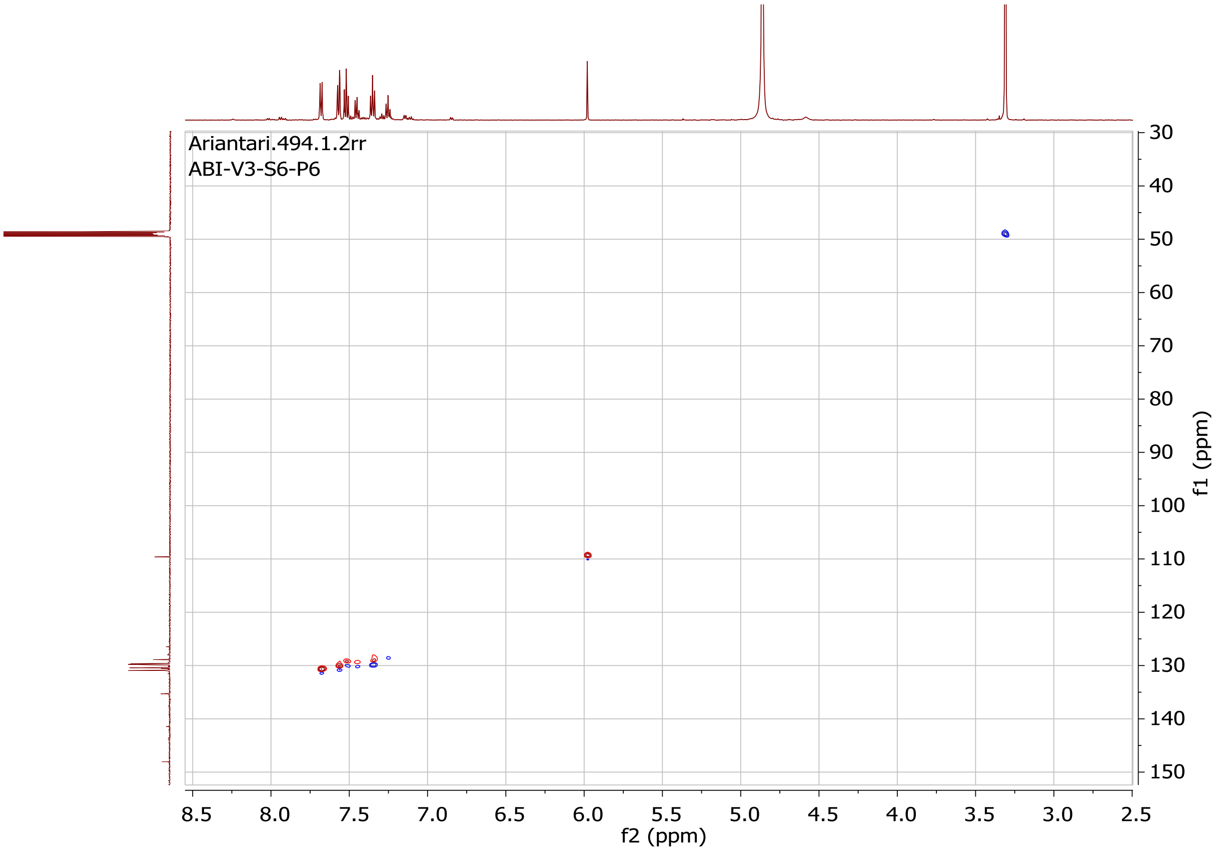

Figure S37. HSQC (600 and 150 MHz, MeOH-d_4) spectrum of compound **5**.

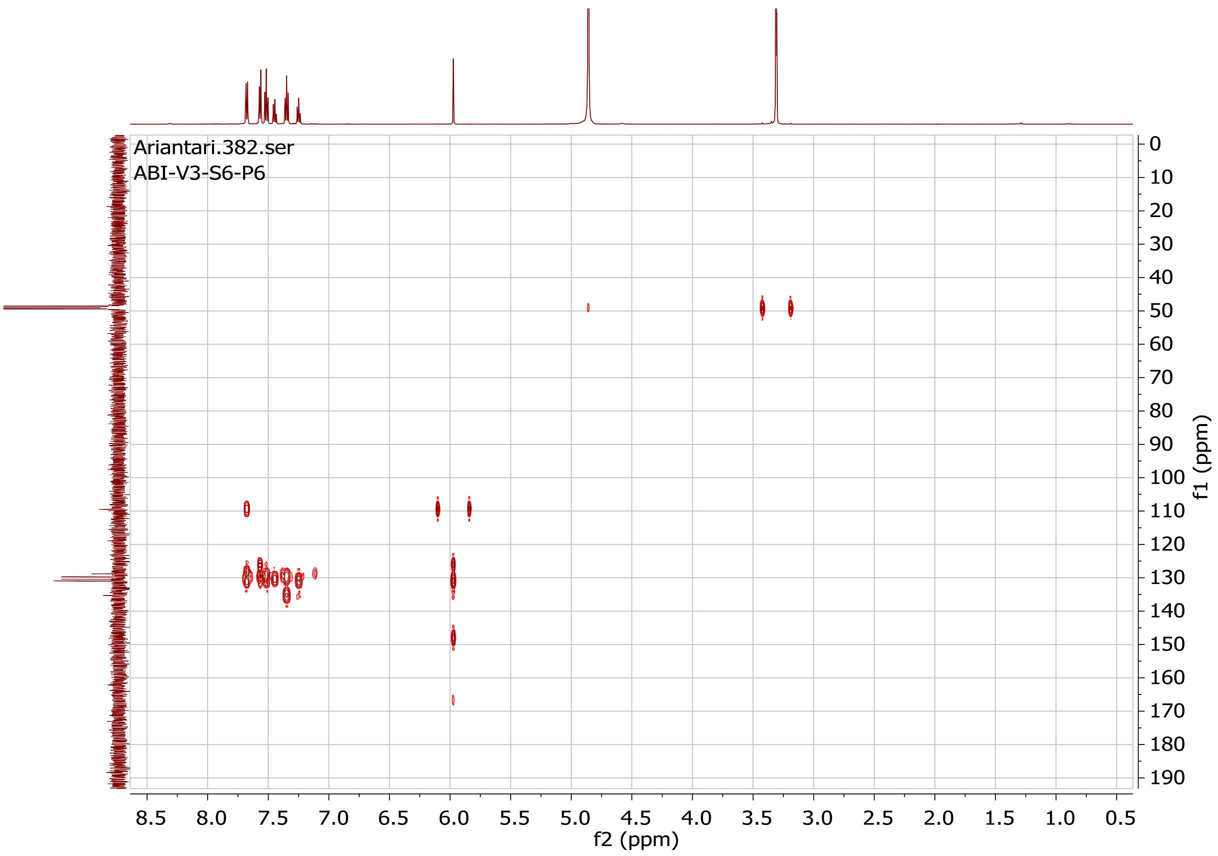

Figure S38. HMBC (600 and 150 MHz, MeOH-d_4) spectrum of compound **5**.

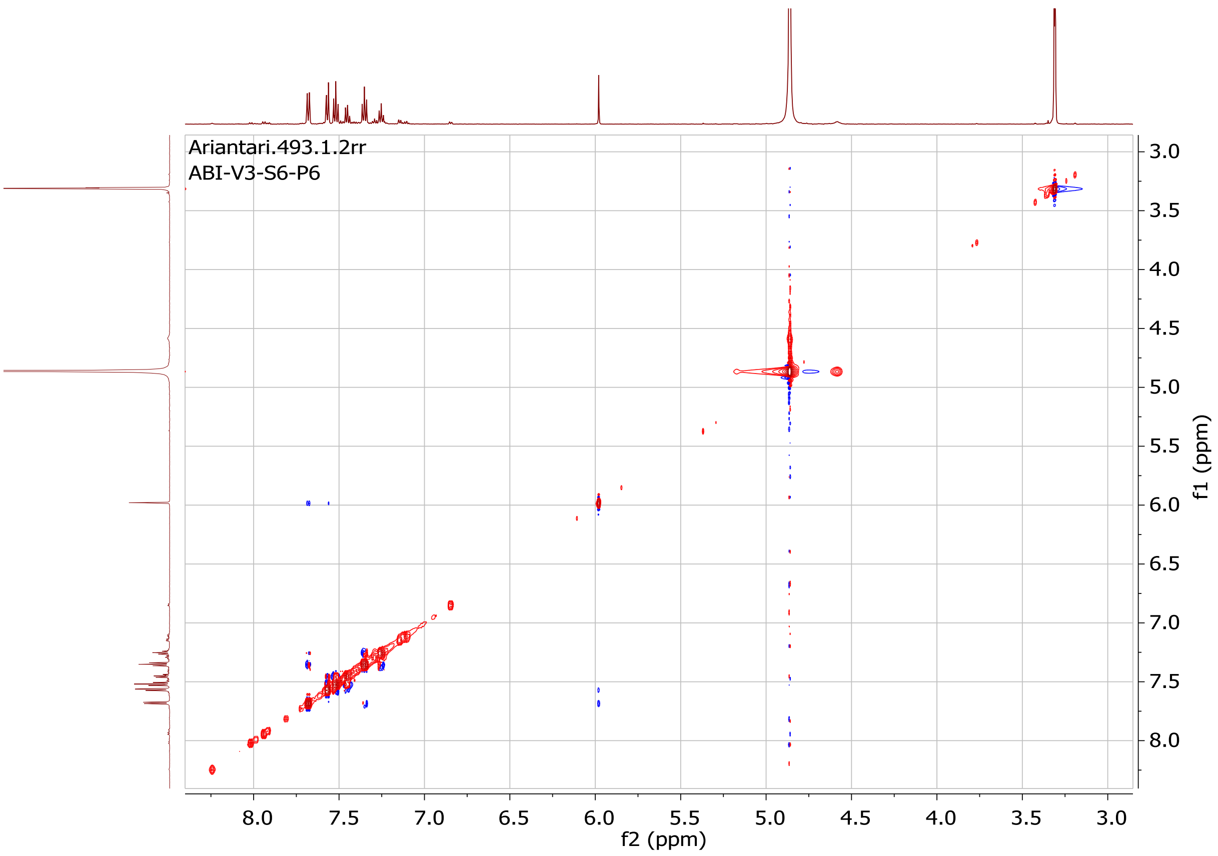

Figure S39. NOESY (600 MHz, MeOH-d_4) spectrum of compound **5**.

(A)

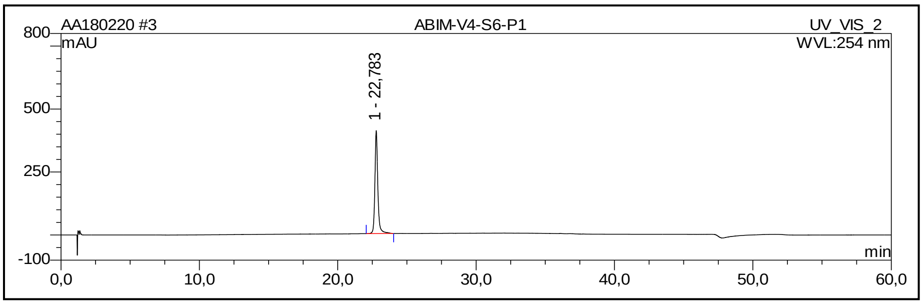

(B)

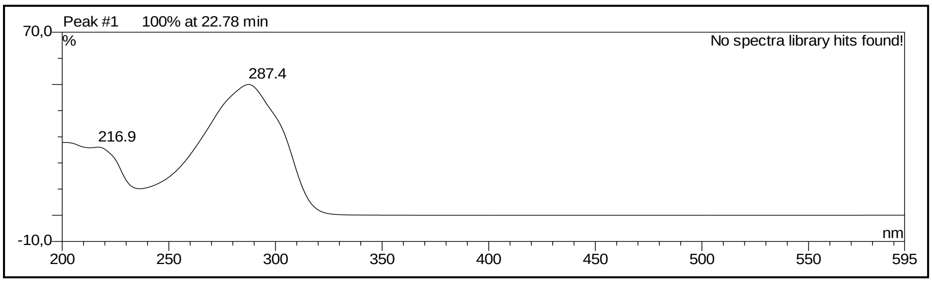

Figure S40. HPLC chromatogram (A) and UV spectrum (B) of compound **6**.

Acquisition Parameter

Source Type	ESI	Ion Polarity	Positive	Set Nebulizer	0.3 Bar
Focus	Not active	Set Capillary	4000 V	Set Dry Heater	180 °C
Scan Begin	50 m/z	Set End Plate Offset	-500 V	Set Dry Gas	4.0 l/min
Scan End	1500 m/z	Set Collision Cell RF	600.0 Vpp	Set Divert Valve	Source

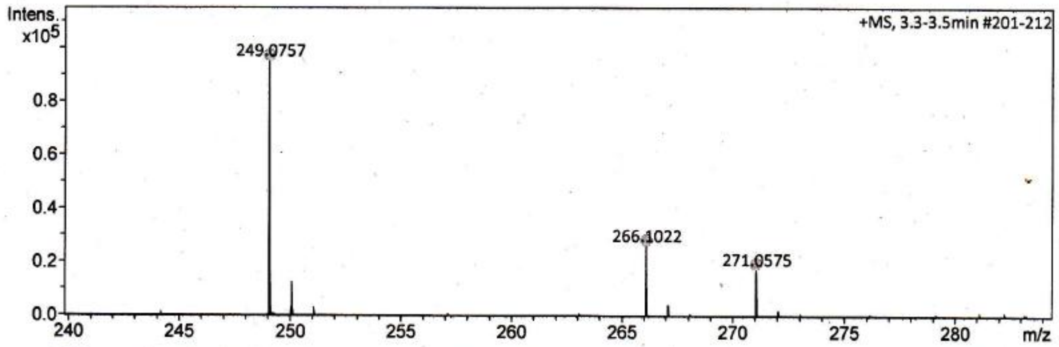

Meas. m/z	#	Ion Formula	m/z	err [ppm]	mSigma	# mSigma	Score	rdb	e⁻ Conf	N-Rule
249.0757	1	C13H13O5	249.0757	0.2	3.9	1	100.00	7.5	even	ok
266.1022	1	C13H16NO5	266.1023	0.5	6.8	1	100.00	6.5	even	ok
271.0575	1	C13H12NaO5	271.0577	0.9	7.1	1	100.00	7.5	even	ok

Figure S41. HRESIMS spectrum of compound **6**.

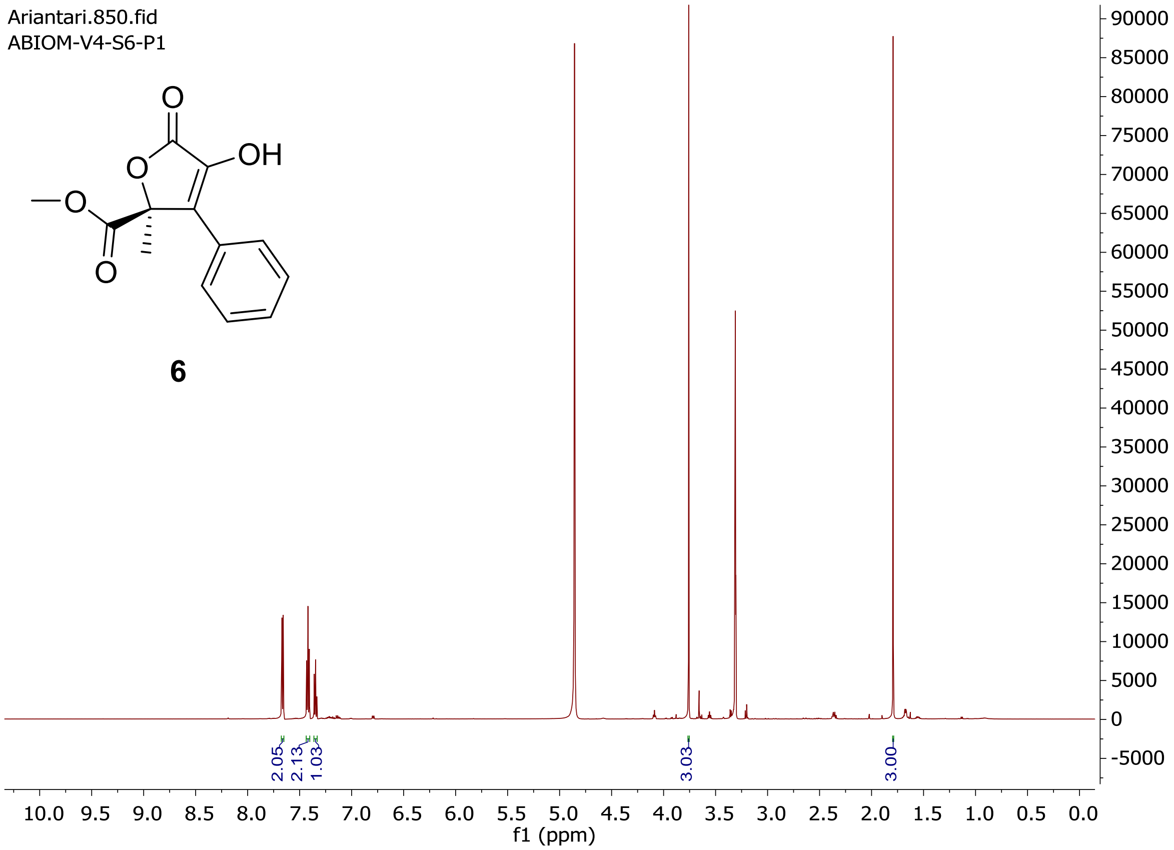

Figure S42. ^{1}H NMR (600 MHz, MeOH-d_4) spectrum of compound **6**.

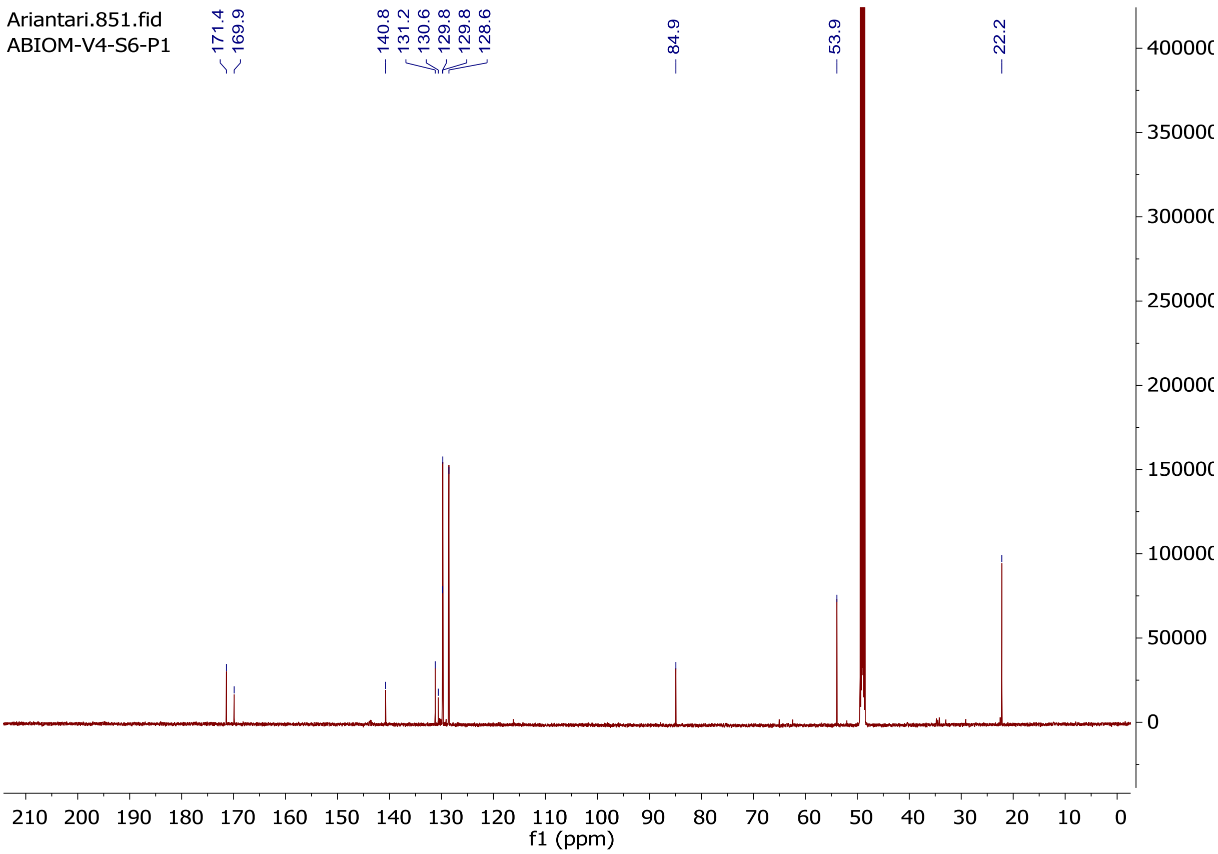

Figure S43. ^{13}C NMR (150 MHz, MeOH-d_4) spectrum of compound **6**.

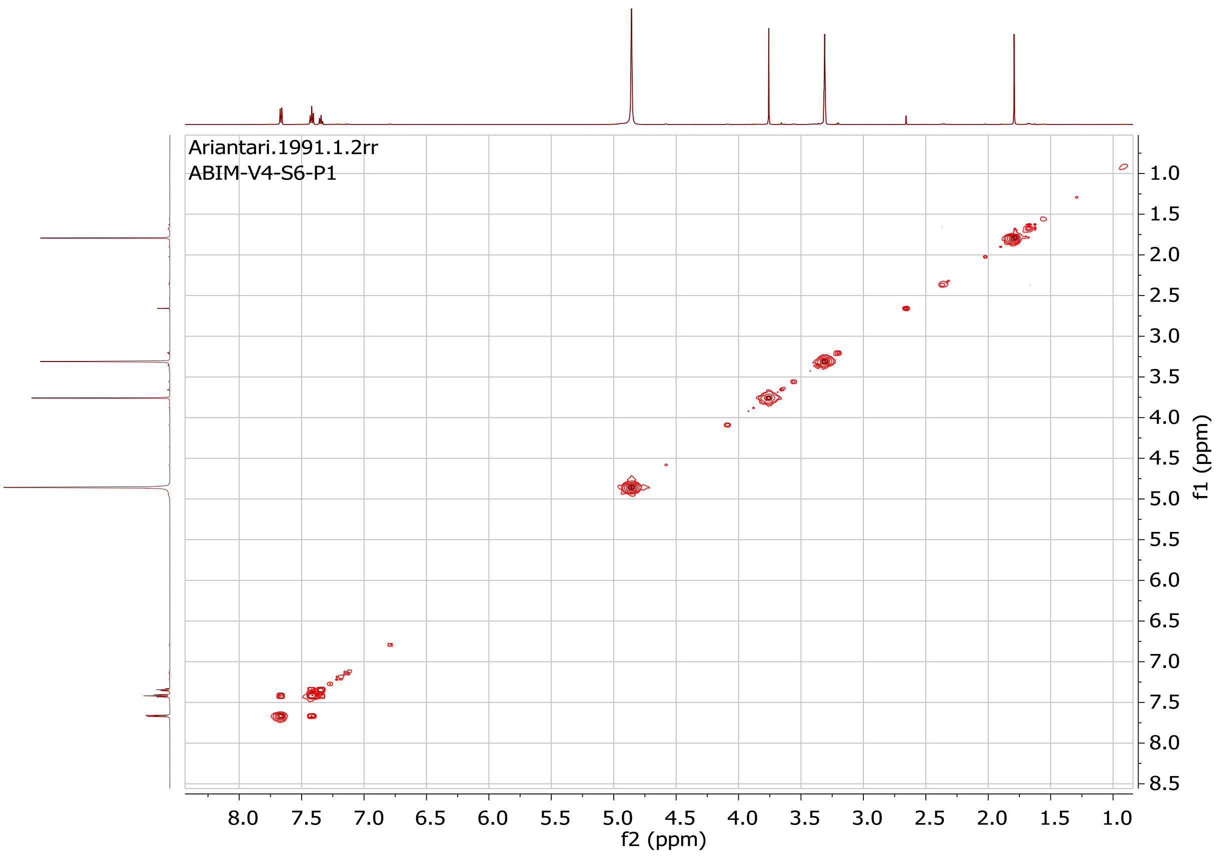

Figure S44. ^{1}H-^{1}H COSY (600 MHz, MeOH-d_4) spectrum of compound **6**.

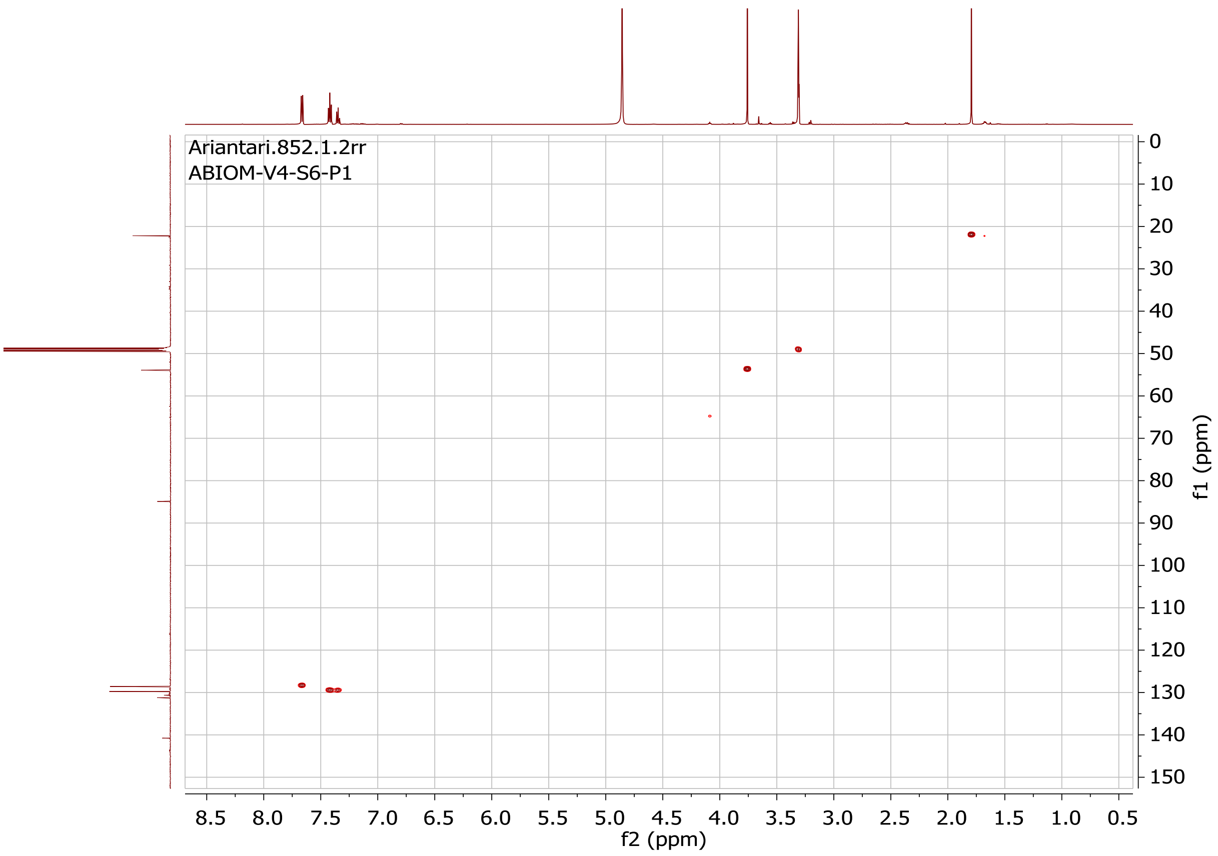

Figure S45. HSQC (600 and 150 MHz, MeOH-d_4) spectrum of compound **6**.

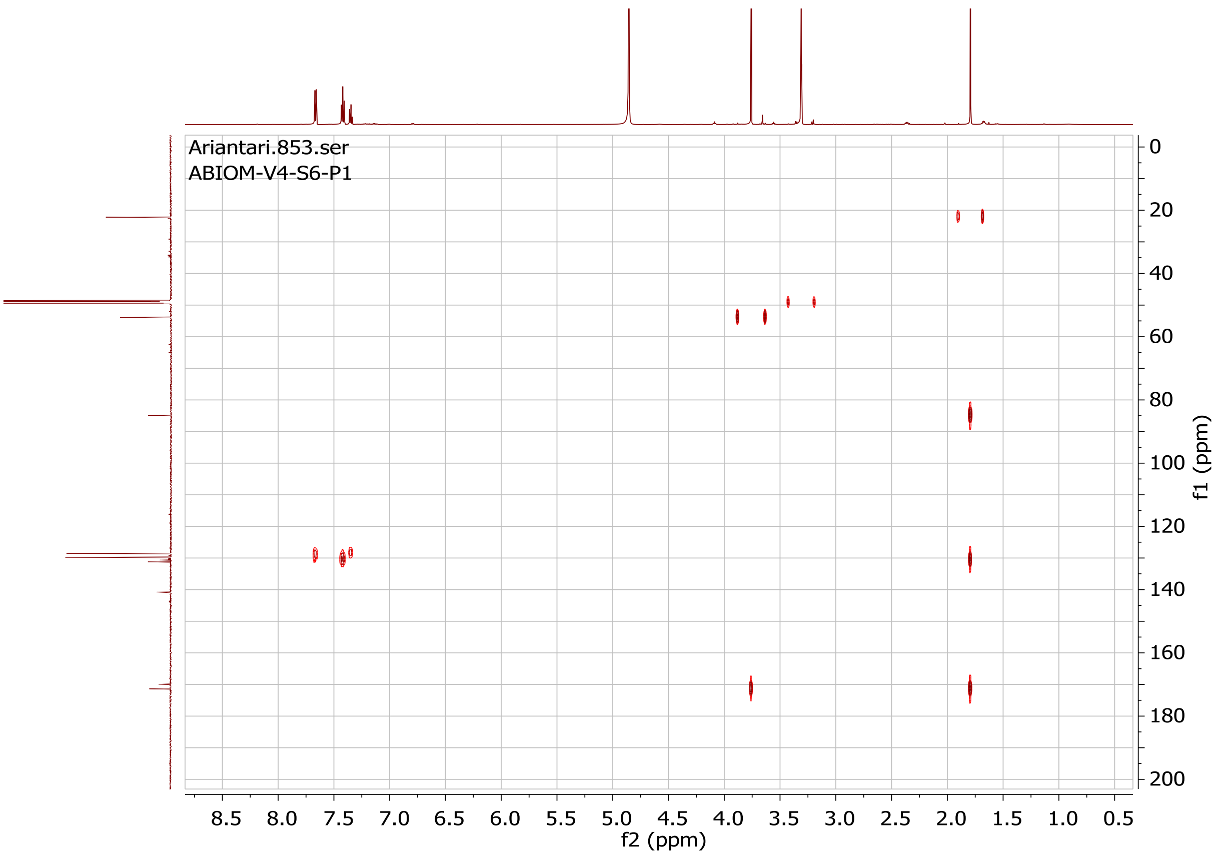

Figure S46. HMBC (600 and 150 MHz, MeOH-d_4) spectrum of compound **6**.

(A)

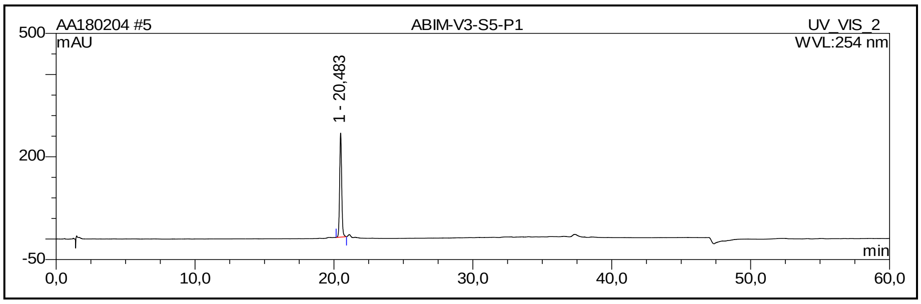

(B)

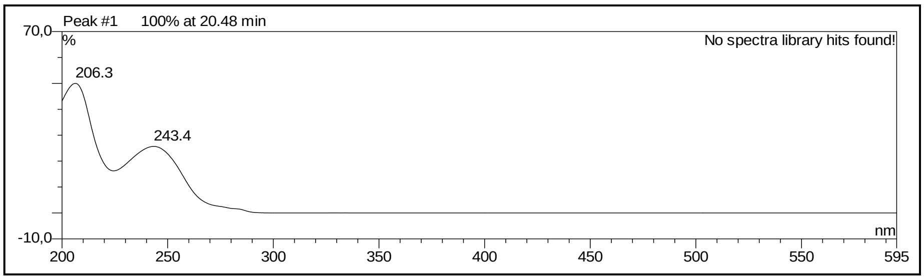

Figure S47. HPLC chromatogram (A) and UV spectrum (B) of compound **7**.

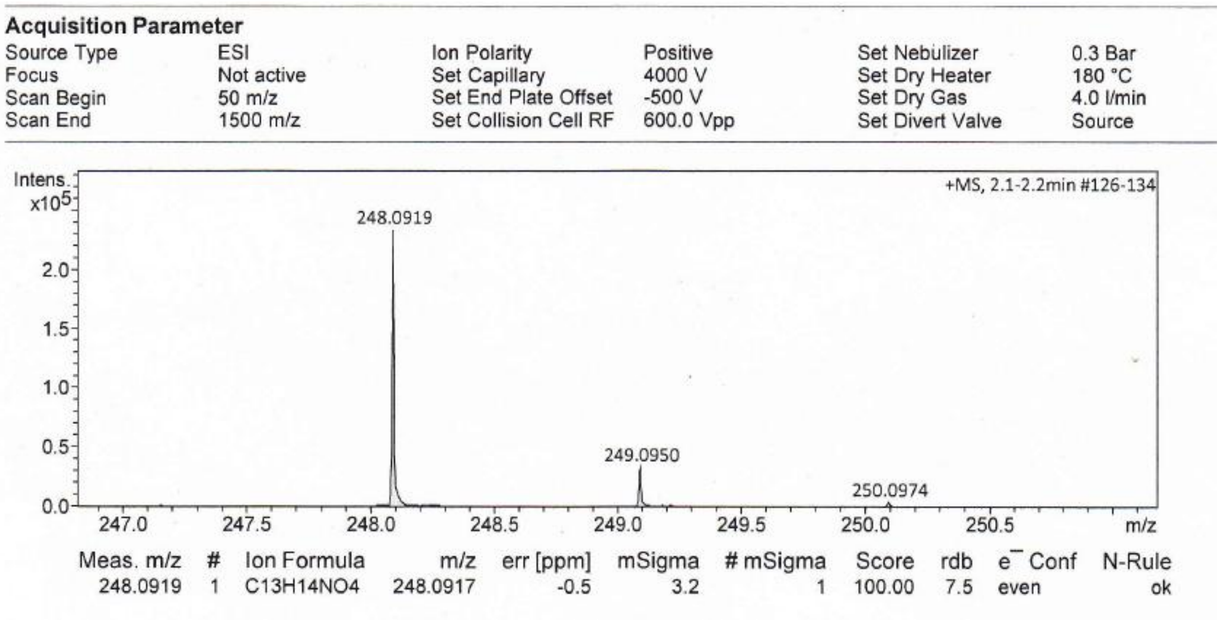

Figure S48. HRESIMS spectrum of compound **7**.

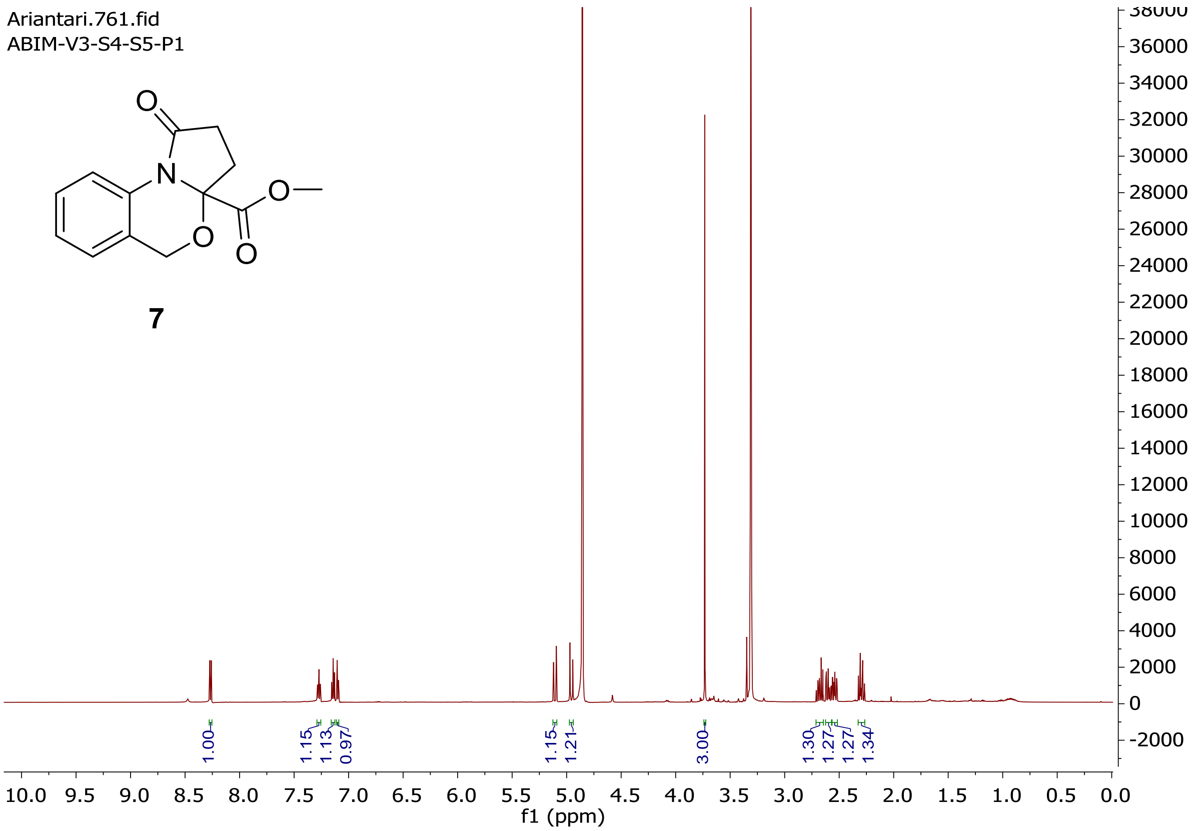

Figure S49. ^{1}H NMR (600 MHz, MeOH-d_4) spectrum of compound **7**.

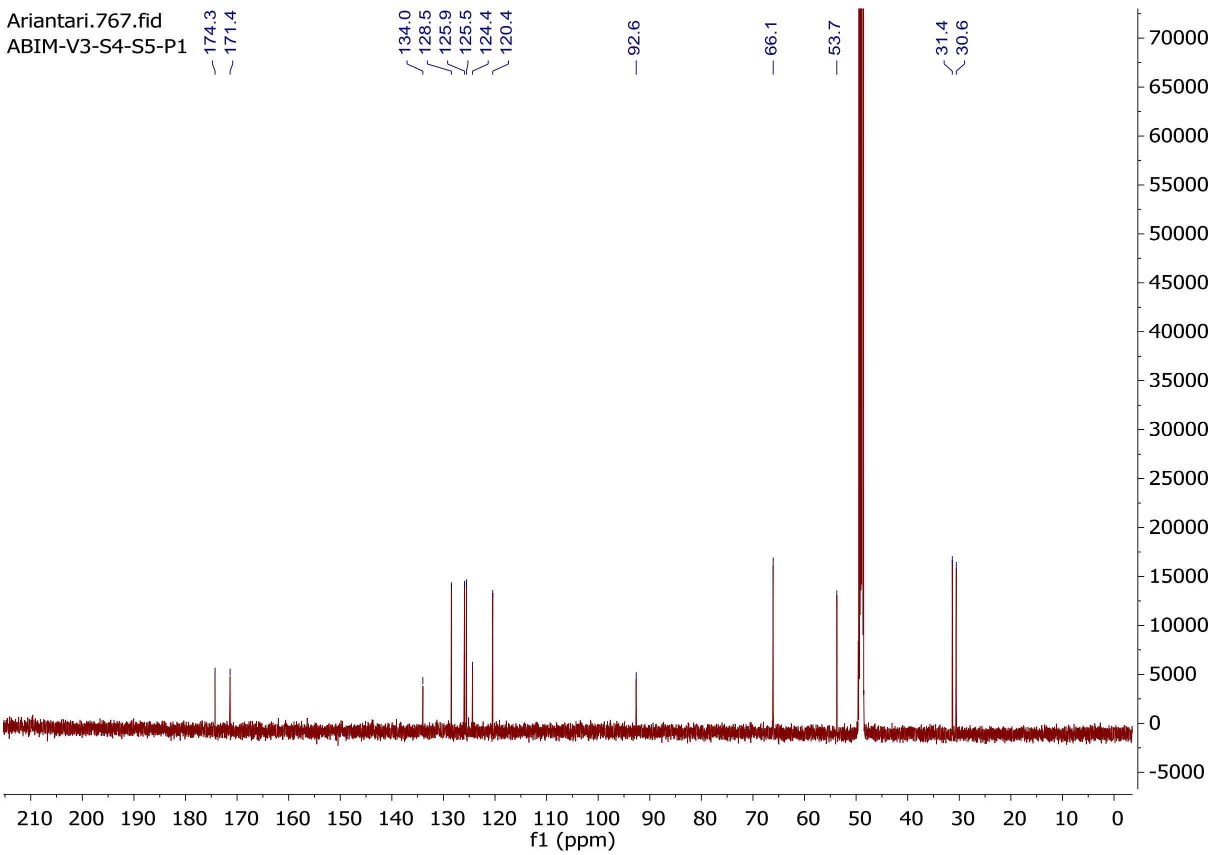

Figure S50. ^{13}C NMR (150 MHz, MeOH-d_4) spectrum of compound **7**.

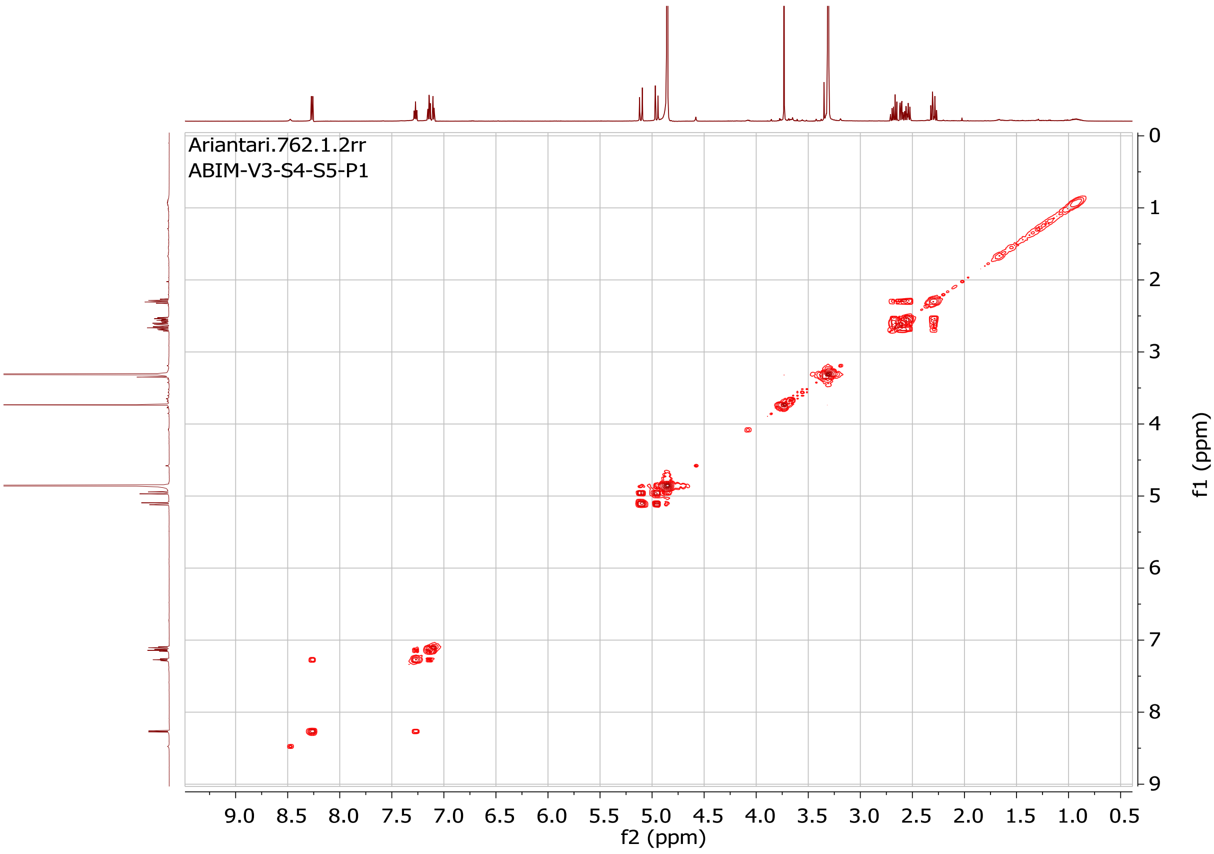

Figure S51. ^{1}H-^{1}H COSY (600 MHz, MeOH-d_4) spectrum of compound **7**.

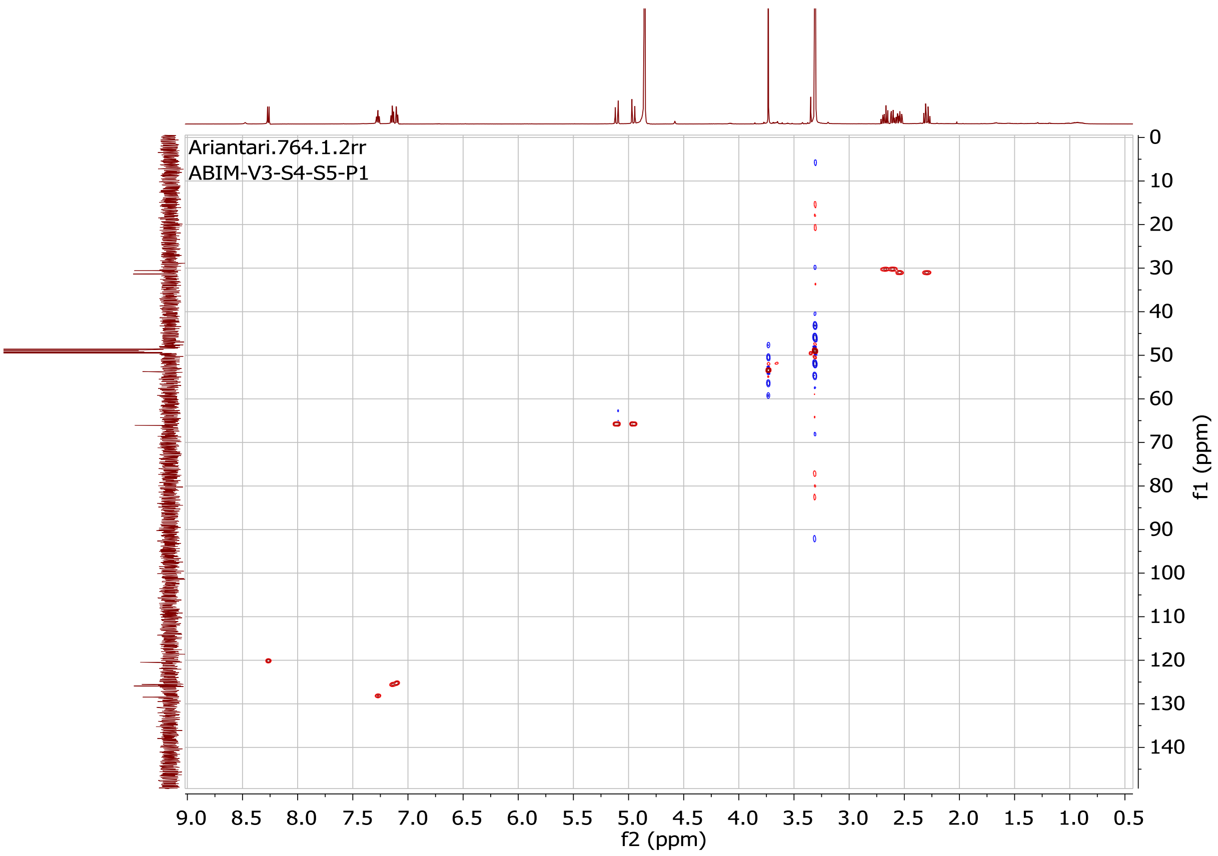

Figure S52. HSQC (600 and 150 MHz, MeOH-d_4) spectrum of compound **7**.

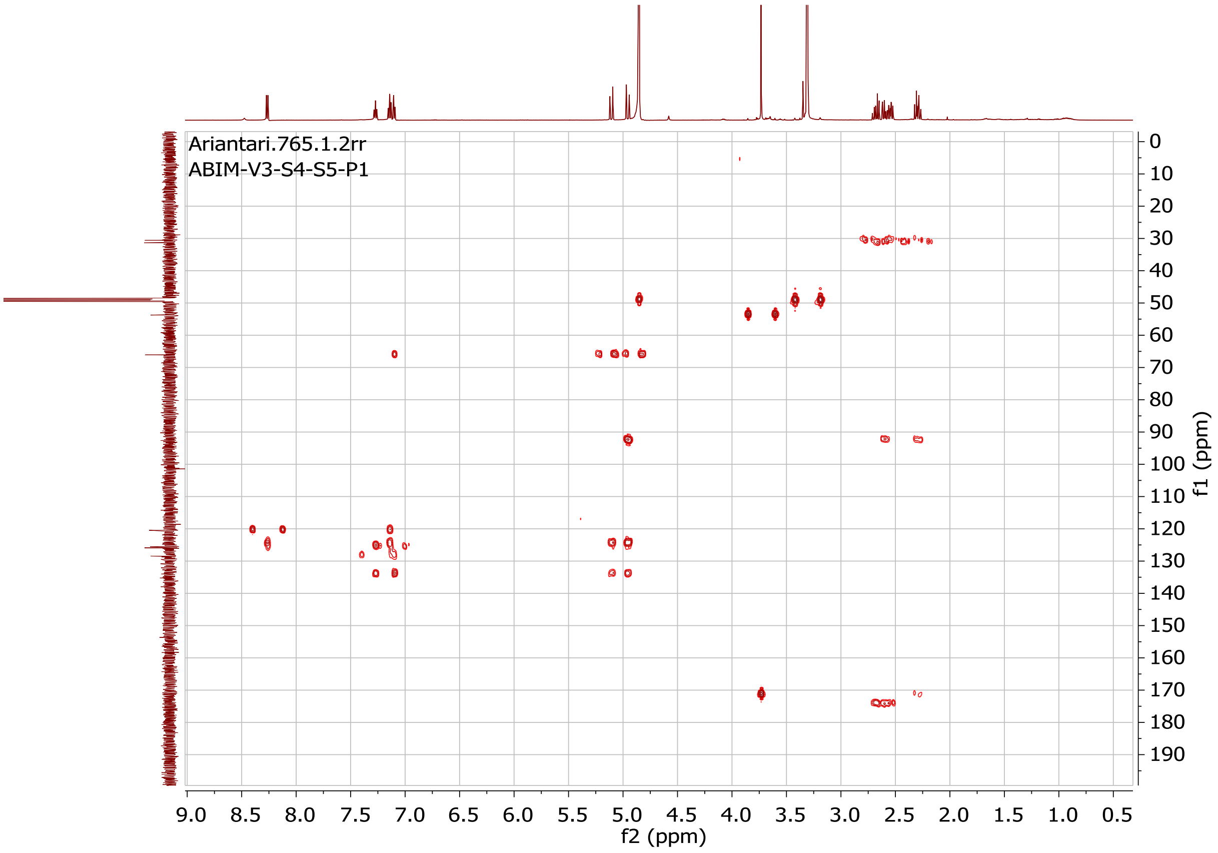

Figure S53. HMBC (600 and 150 MHz, MeOH-d_4) spectrum of compound **7**.

(A)

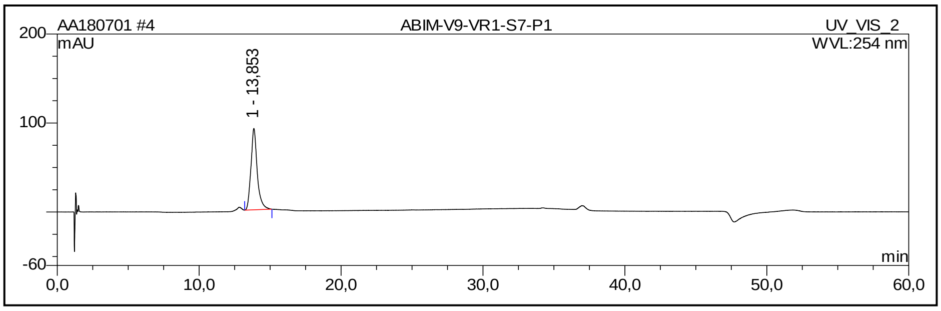

(B)

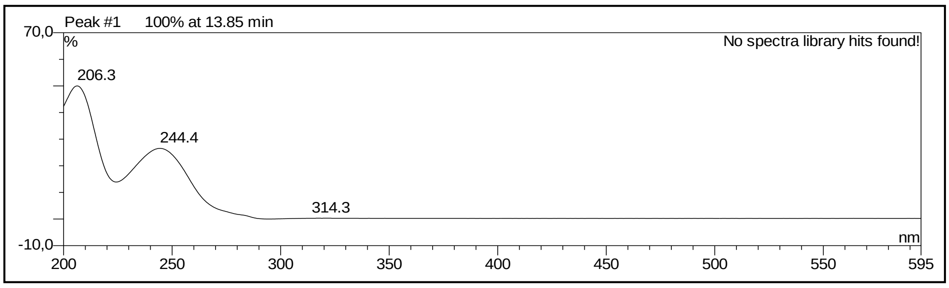

Figure S54. HPLC chromatogram (A) and UV spectrum (B) of compound **8**.

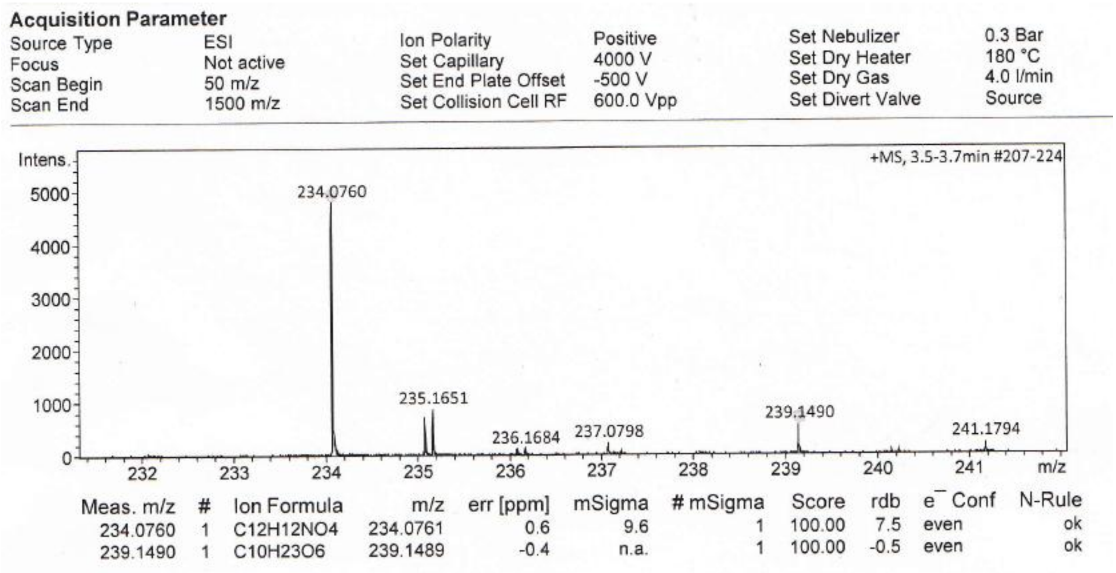

Figure S55. HRESIMS spectrum of compound **8**.

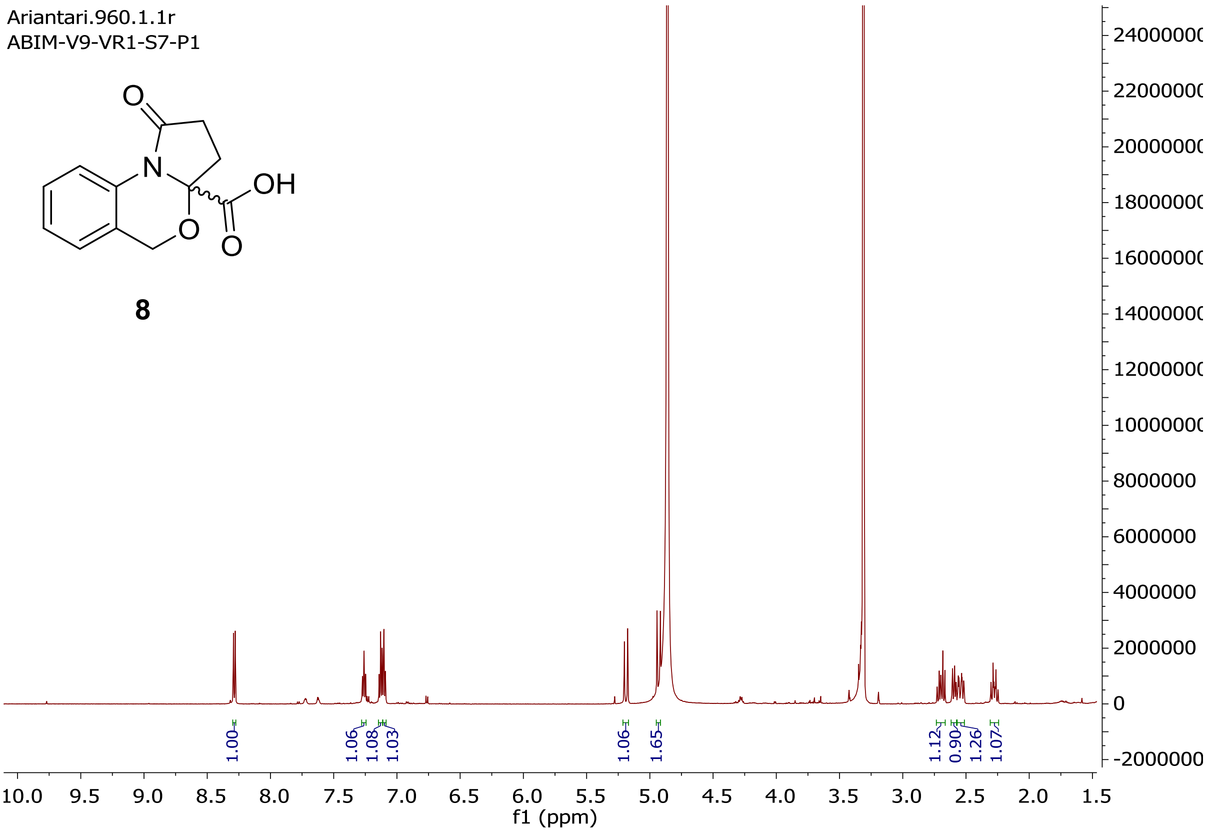

Figure S56. ^{1}H NMR (600 MHz, MeOH-d_4) spectrum of compound **8**.

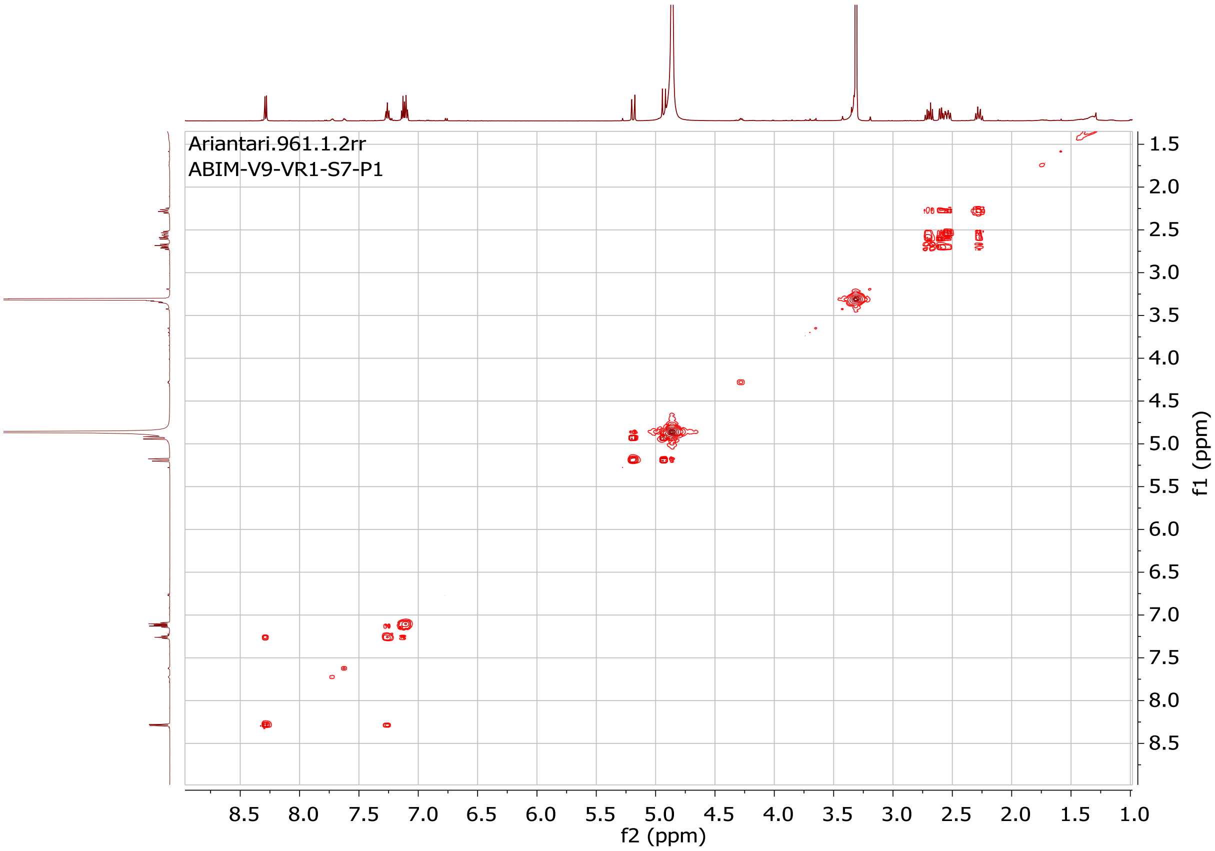

Figure S57. ^{1}H-^{1}H COSY (600 MHz, MeOH-d_4) spectrum of compound **8**.

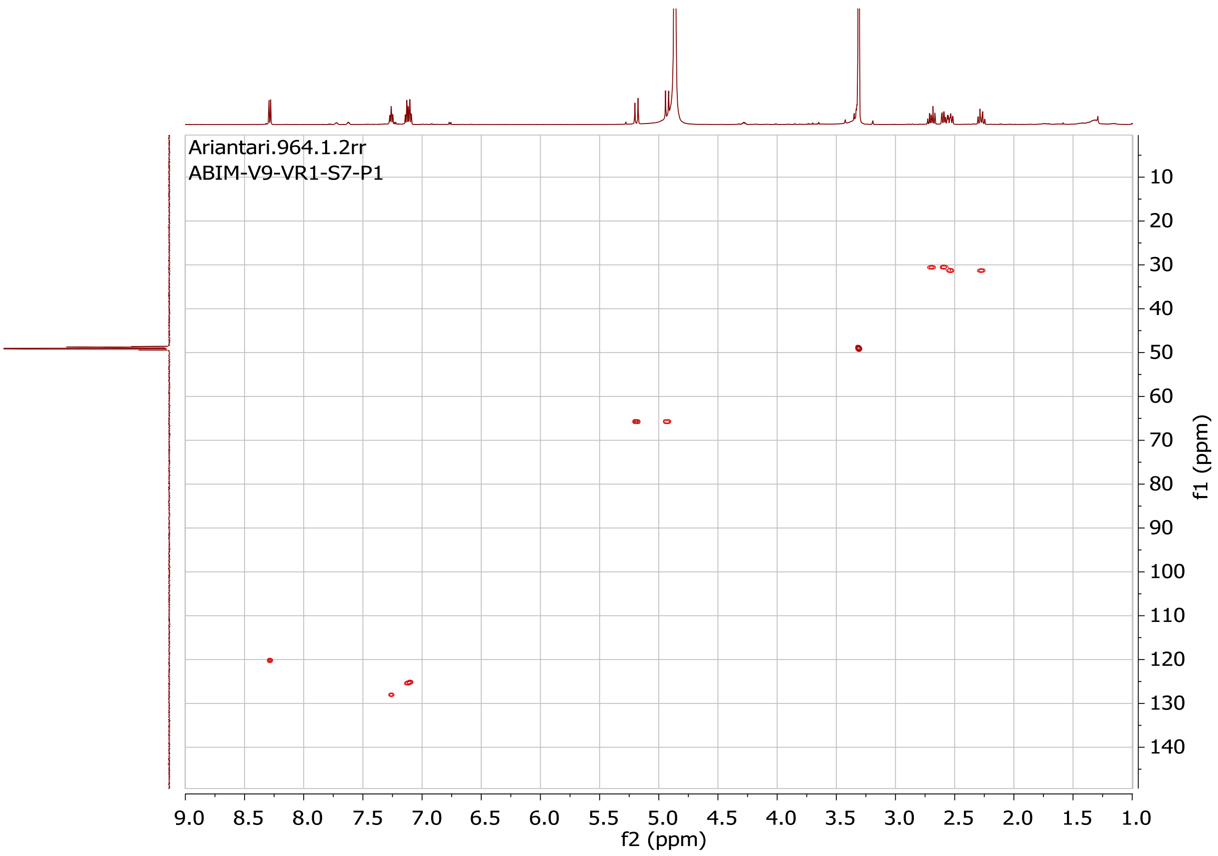

Figure S58. HSQC (600 and 150 MHz, MeOH-d_4) spectrum of compound **8**.

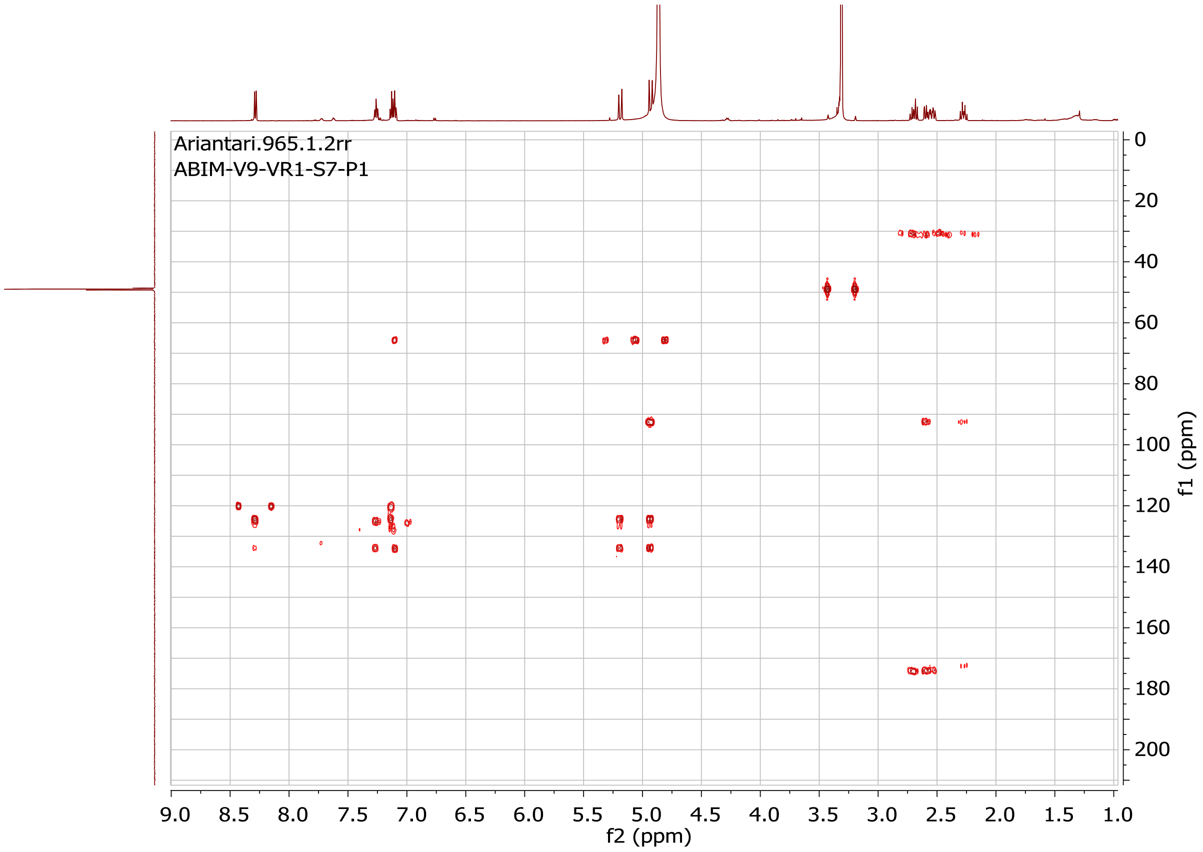

Figure S59. HMBC (600 and 150 MHz, MeOH-d_4) spectrum of compound **8**.

(A)

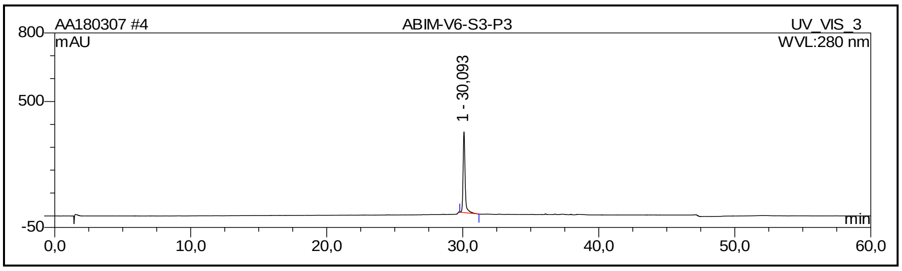

(B)

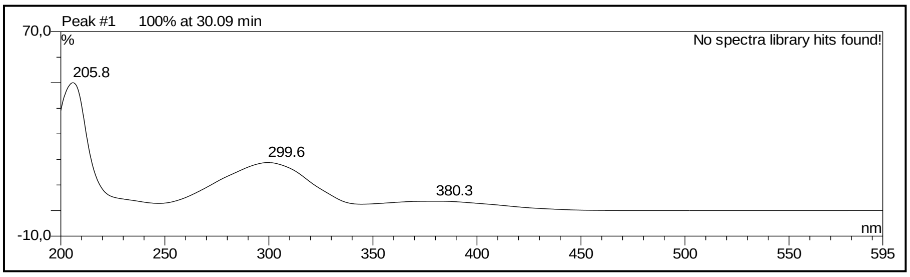

Figure S60. HPLC chromatogram (A) and UV spectrum (B) of compound **9**.

Acquisition Parameter

Source Type	ESI	Ion Polarity	Positive	Set Nebulizer	0.3 Bar
Focus	Not active	Set Capillary	4000 V	Set Dry Heater	180 °C
Scan Begin	50 m/z	Set End Plate Offset	-500 V	Set Dry Gas	4.0 l/min
Scan End	1500 m/z	Set Collision Cell RF	600.0 Vpp	Set Divert Valve	Source

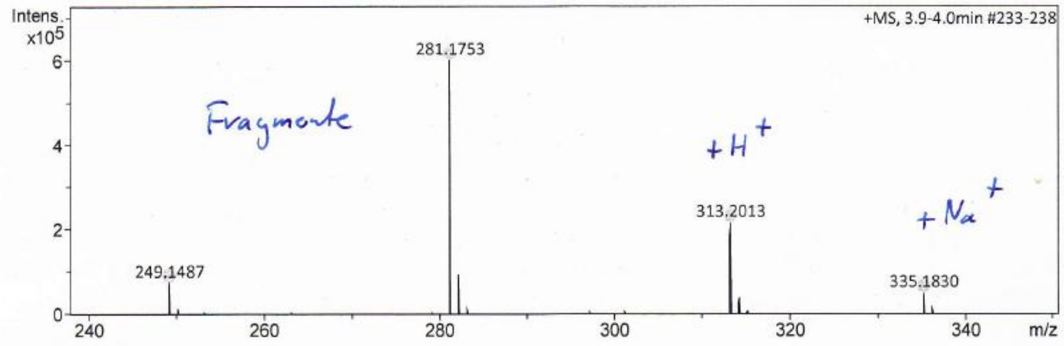

Meas. m/z	#	Ion Formula	m/z	err [ppm]	mSigma	# mSigma	Score	rdb	e⁻ Conf	N-Rule
249.1487	1	C15H21O3	249.1485	-0.8	3.1	1	100.00	5.5	even	ok
281.1753	1	C16H25O4	281.1747	-2.0	13.4	1	100.00	4.5	even	ok
	2	C17H21N4	281.1761	2.8	26.9	2	66.88	9.5	even	ok
313.2013	1	C17H29O5	313.2010	-1.3	3.9	1	100.00	3.5	even	ok
	2	C18H25N4O	313.2023	3.0	17.4	2	58.64	8.5	even	ok
335.1830	1	C17H28NaO5	335.1829	-0.3	5.8	1	100.00	3.5	even	ok
	2	C18H24N4NaO	335.1842	3.7	8.8	2	52.60	8.5	even	ok

Figure S61. HRESIMS spectrum of compound **9**.

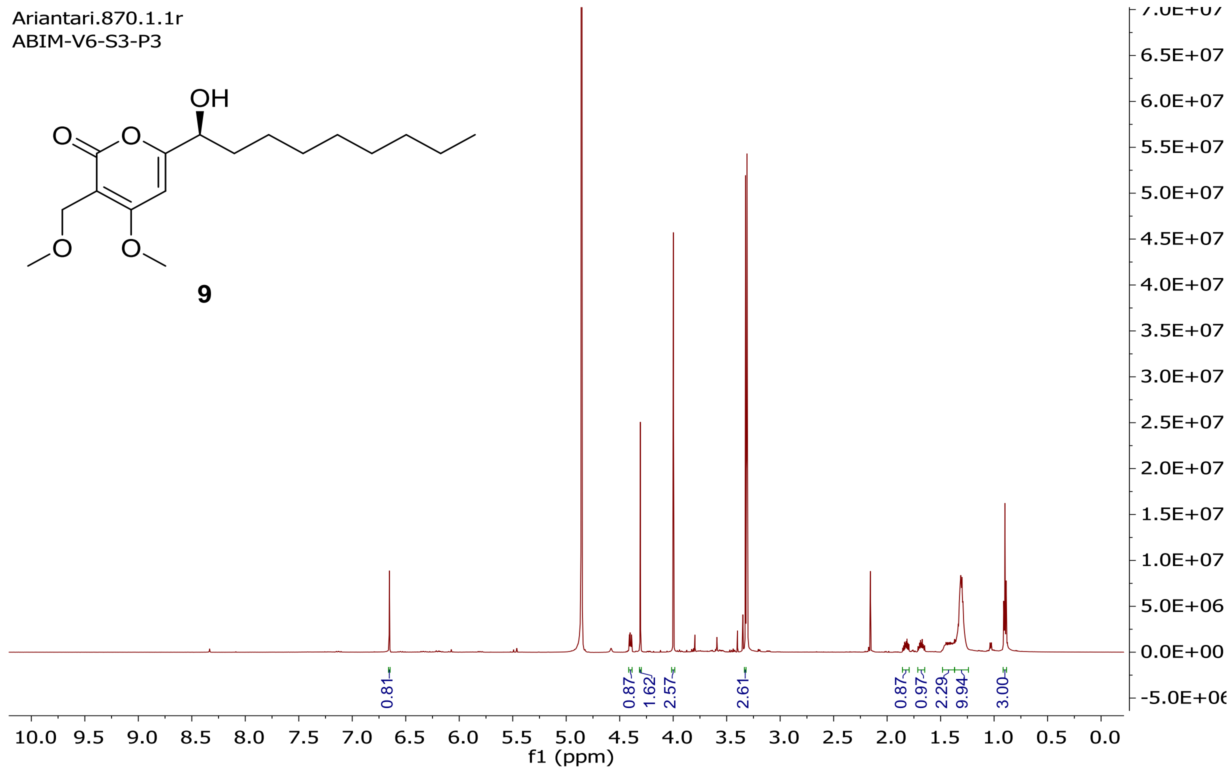

Figure S62. ^{1}H NMR (600 MHz, MeOH-d_4) spectrum of compound **9**.

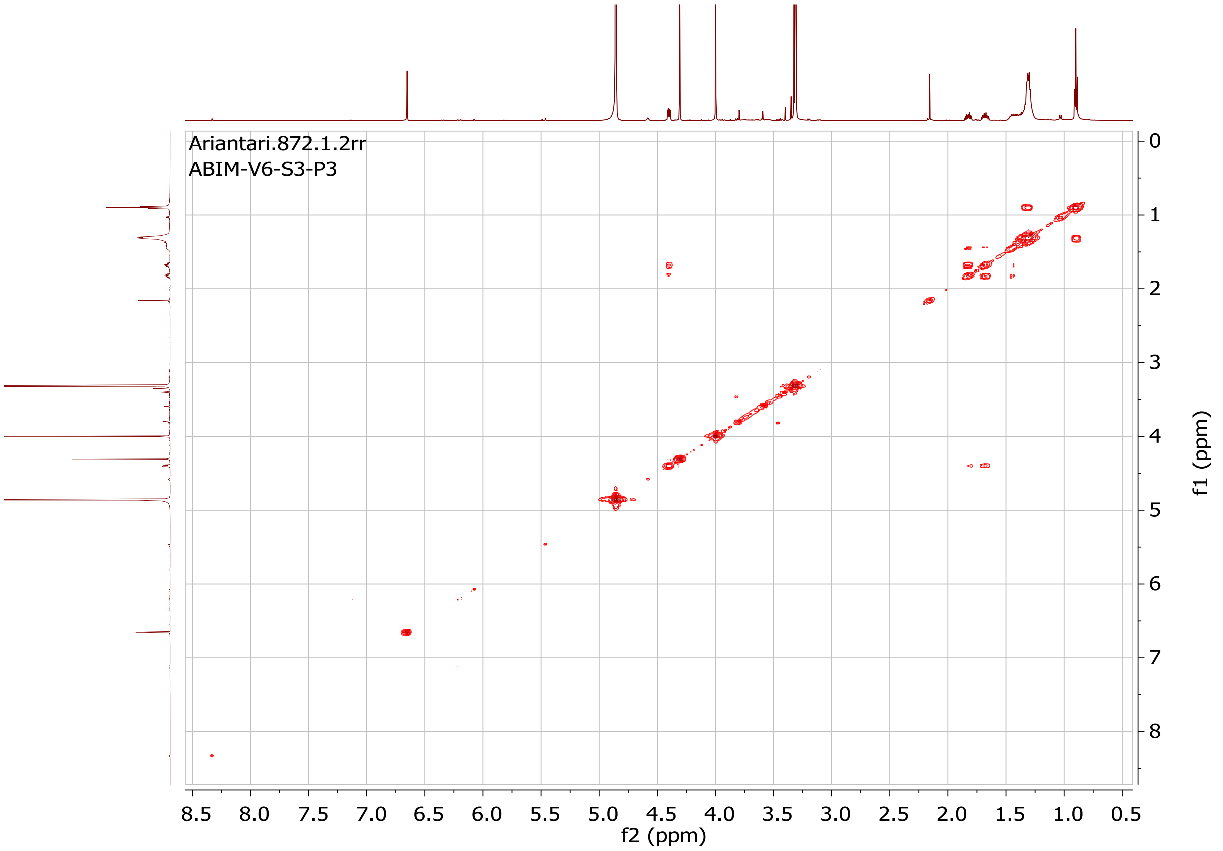

Figure S63. ^{1}H-^{1}H COSY (600 MHz, MeOH-d_4) spectrum of compound **9**.

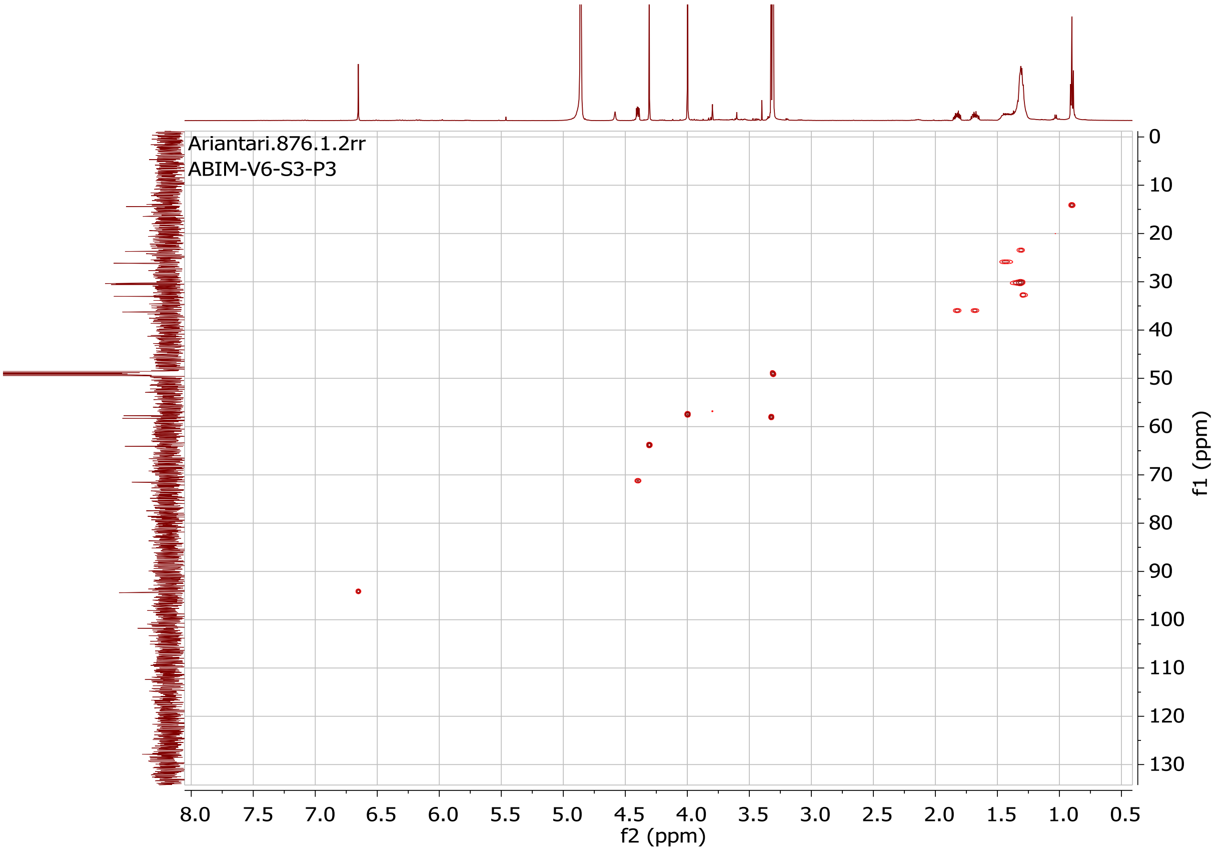

Figure S64. HSQC (600 and 150 MHz, MeOH-d_4) spectrum of compound **9**.

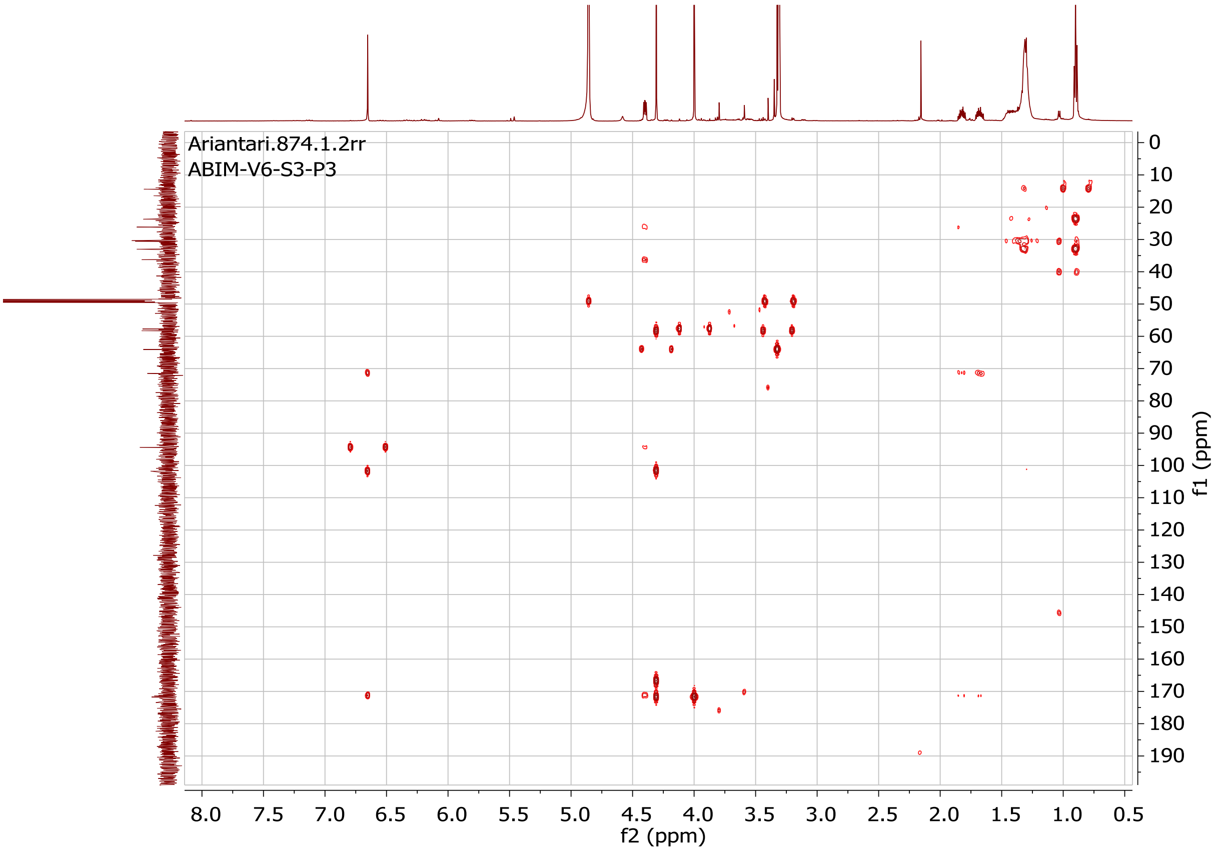

Figure S65. HMBC (600 and 150 MHz, MeOH-d_4) spectrum of compound **9**.

(A)

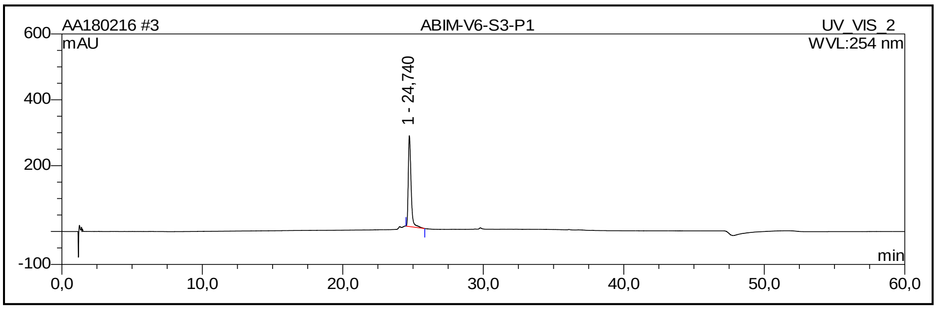

(B)

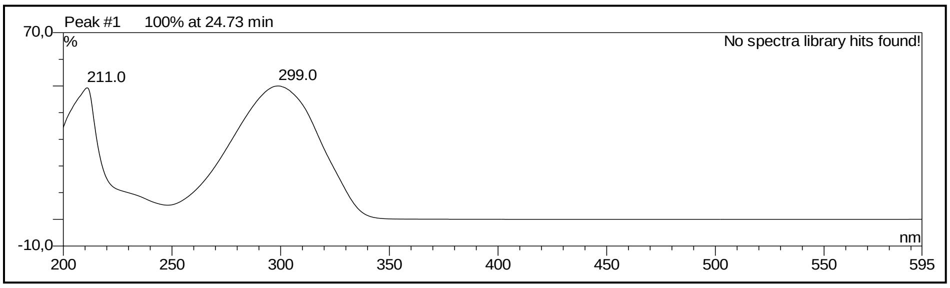

Figure S66. HPLC chromatogram (A) and UV spectrum (B) of compound **10**.

Acquisition Parameter

Source Type	ESI	Ion Polarity	Positive	Set Nebulizer	0.3 Bar		
Focus	Not active	Set Capillary	4000 V	Set Dry Heater	180 °C		
Scan Begin	50 m/z	Set End Plate Offset	-500 V	Set Dry Gas	4.0 l/min		
Scan End	1500 m/z	Set Collision Cell RF	600.0 Vpp	Set Divert Valve	Source		

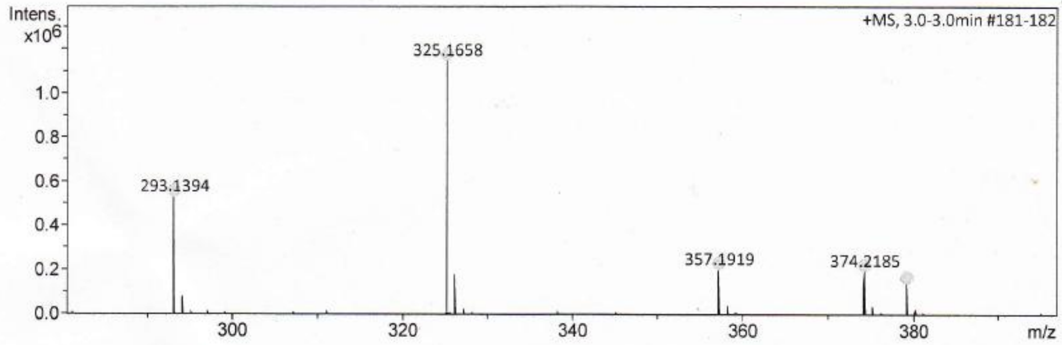

Meas. m/z	#	Ion Formula	m/z	err [ppm]	mSigma	# mSigma	Score	rdb	e⁻ Conf	N-Rule
293.1394	1	C16H21O5	293.1384	-3.6	13.4	1	89.06	6.5	even	ok
	2	C17H17N4O	293.1397	1.0	26.3	2	100.00	11.5	even	ok
325.1658	1	C17H25O6	325.1646	-3.7	18.5	1	78.77	5.5	even	ok
	2	C18H21N4O2	325.1659	0.4	31.6	2	100.00	10.5	even	ok
357.1919	1	C18H29O7	357.1908	-3.2	0.7	1	76.93	4.5	even	ok
	2	C19H25N4O3	357.1921	0.5	14.1	2	100.00	9.5	even	ok
374.2185	1	C18H32NO7	374.2173	-3.1	8.1	1	78.34	3.5	even	ok
	2	C19H28N5O3	374.2187	0.5	21.5	2	100.00	8.5	even	ok
379.1739	1	C18H28NaO7	379.1727	-3.1	2.4	1	76.65	4.5	even	ok
	2	C19H24N4NaO3	379.1741	0.4	16.1	2	100.00	9.5	even	ok

Figure S67. HRESIMS spectrum of compound **10**.

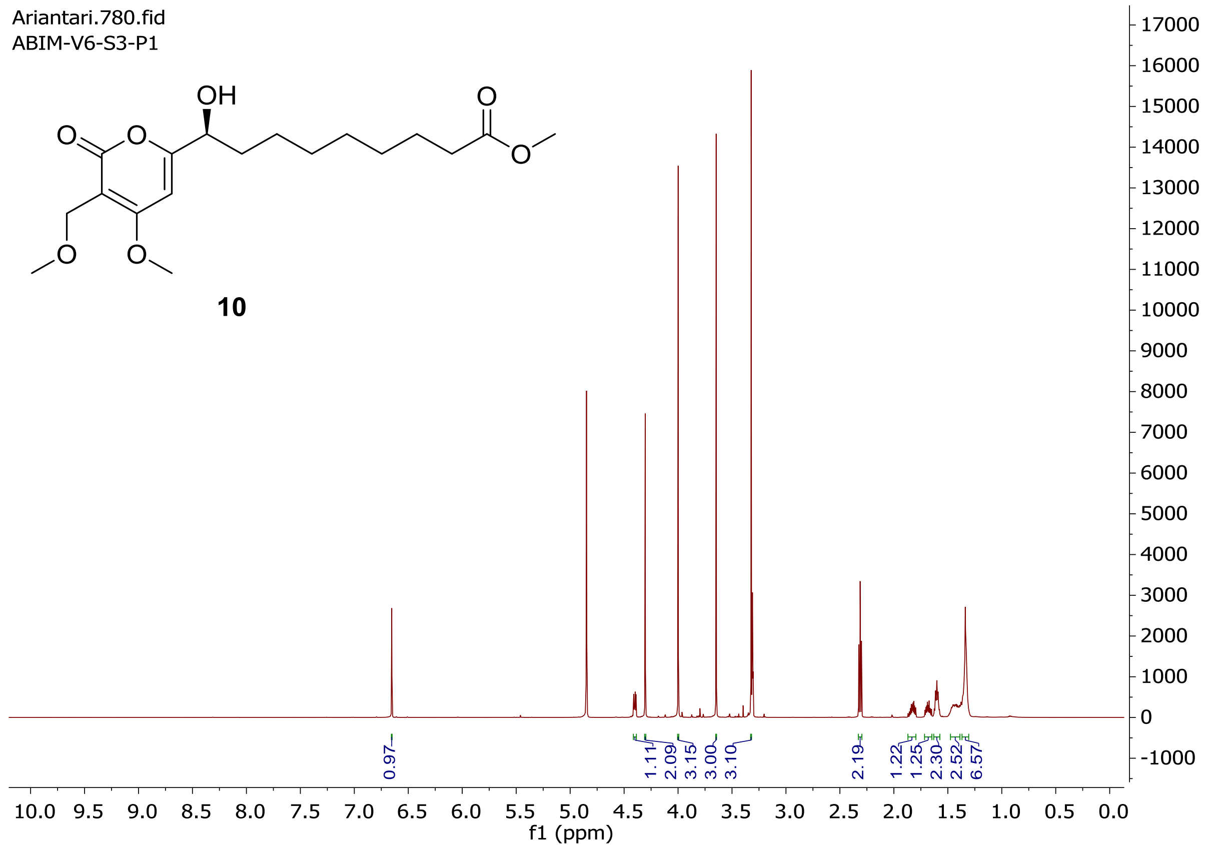

Figure S68. ^{1}H NMR (600 MHz, MeOH-d_4) spectrum of compound **10**.

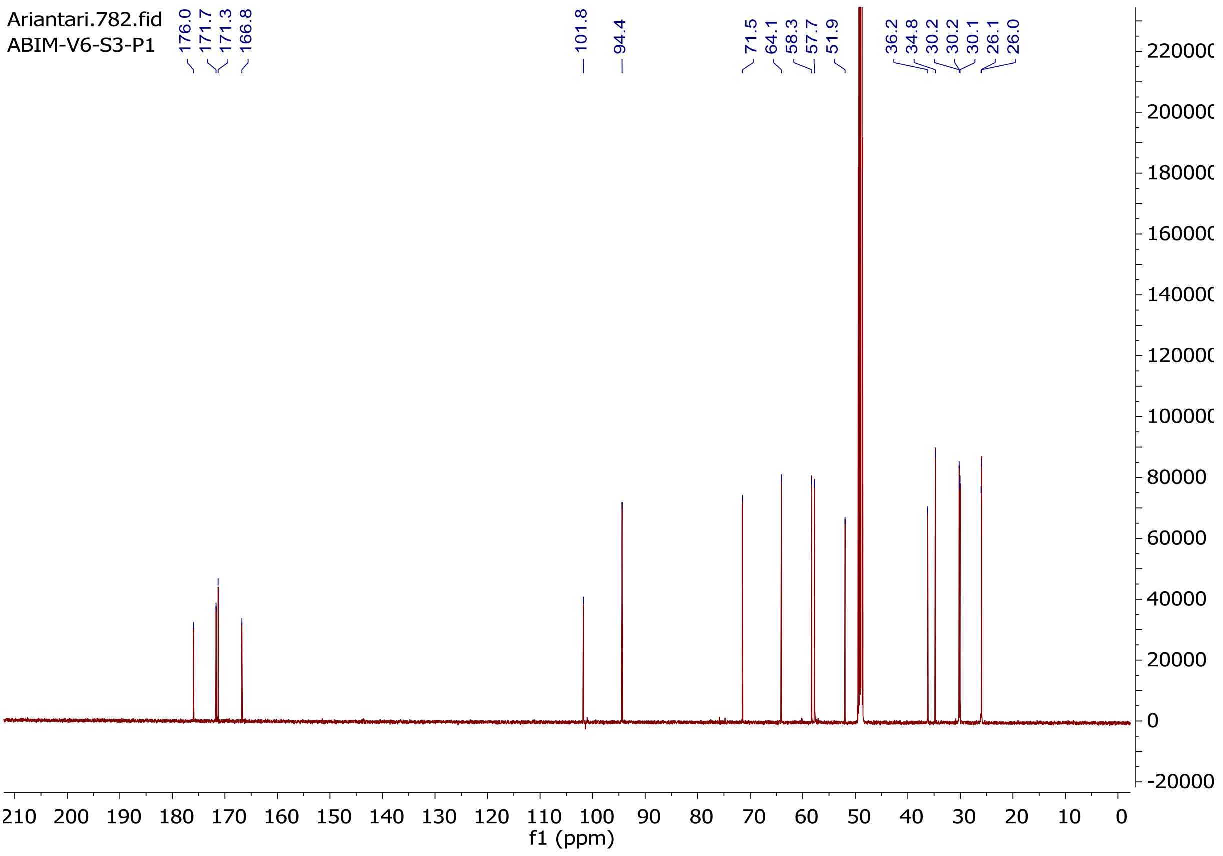

Figure S69. ^{13}C NMR (150 MHz, MeOH-d_4) spectrum of compound **10**.

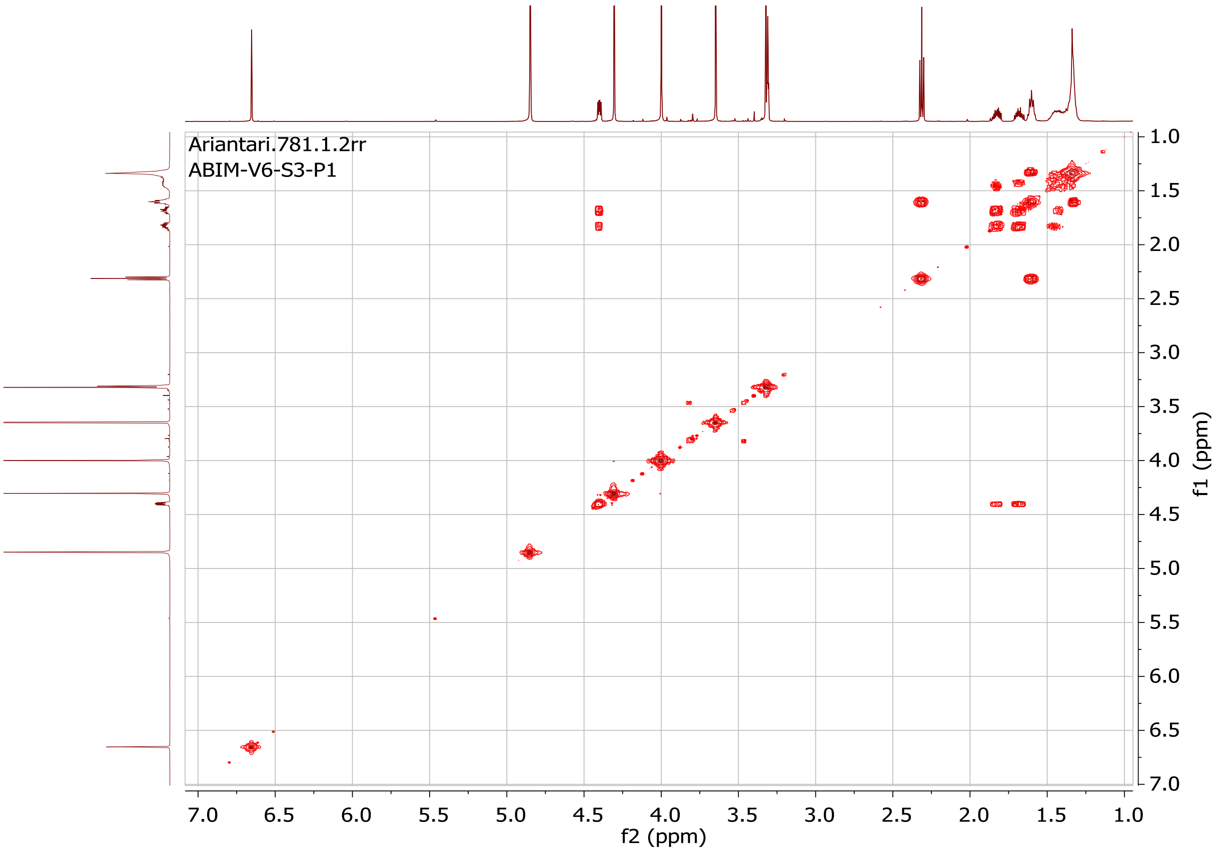

Figure S70. ^1H-^1H COSY (600 MHz, MeOH-d_4) spectrum of compound **10**.

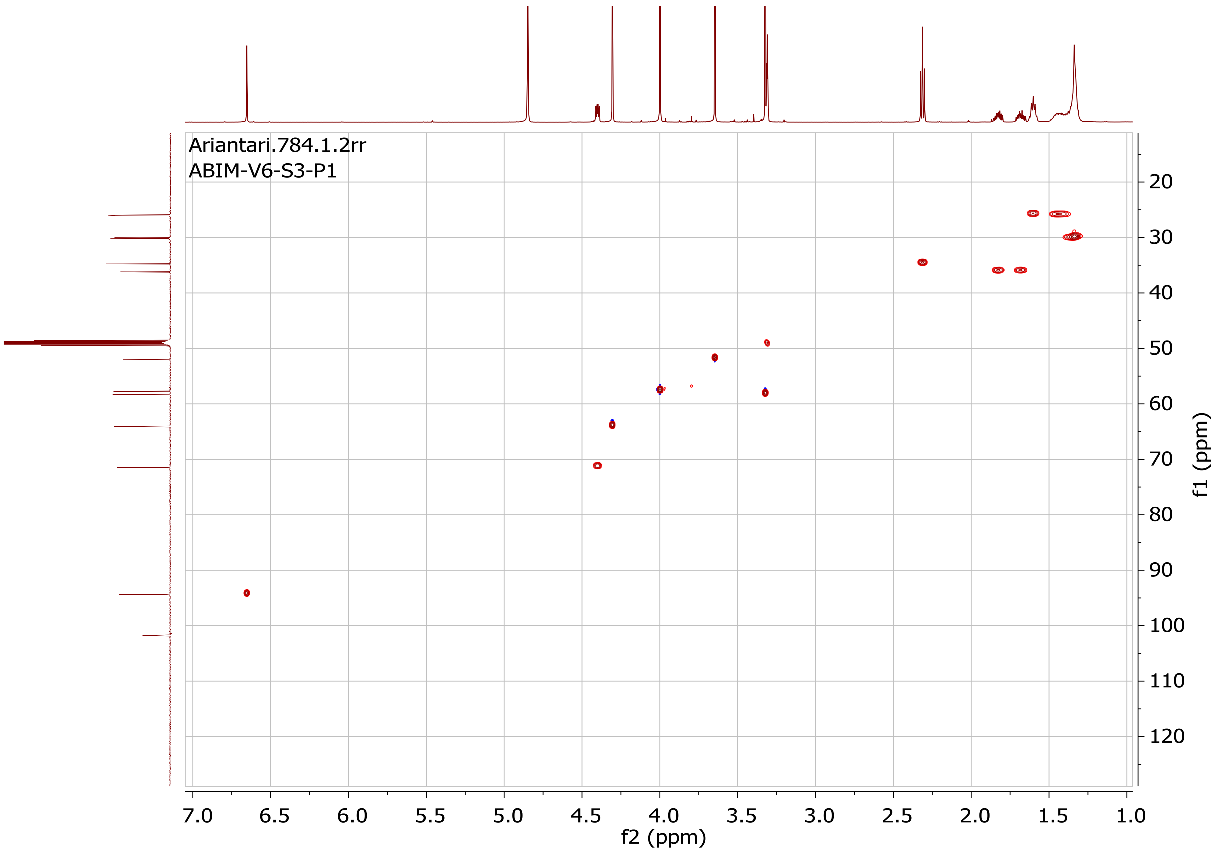

Figure S71. HSQC (600 and 150 MHz, MeOH-d_4) spectrum of compound **10**.

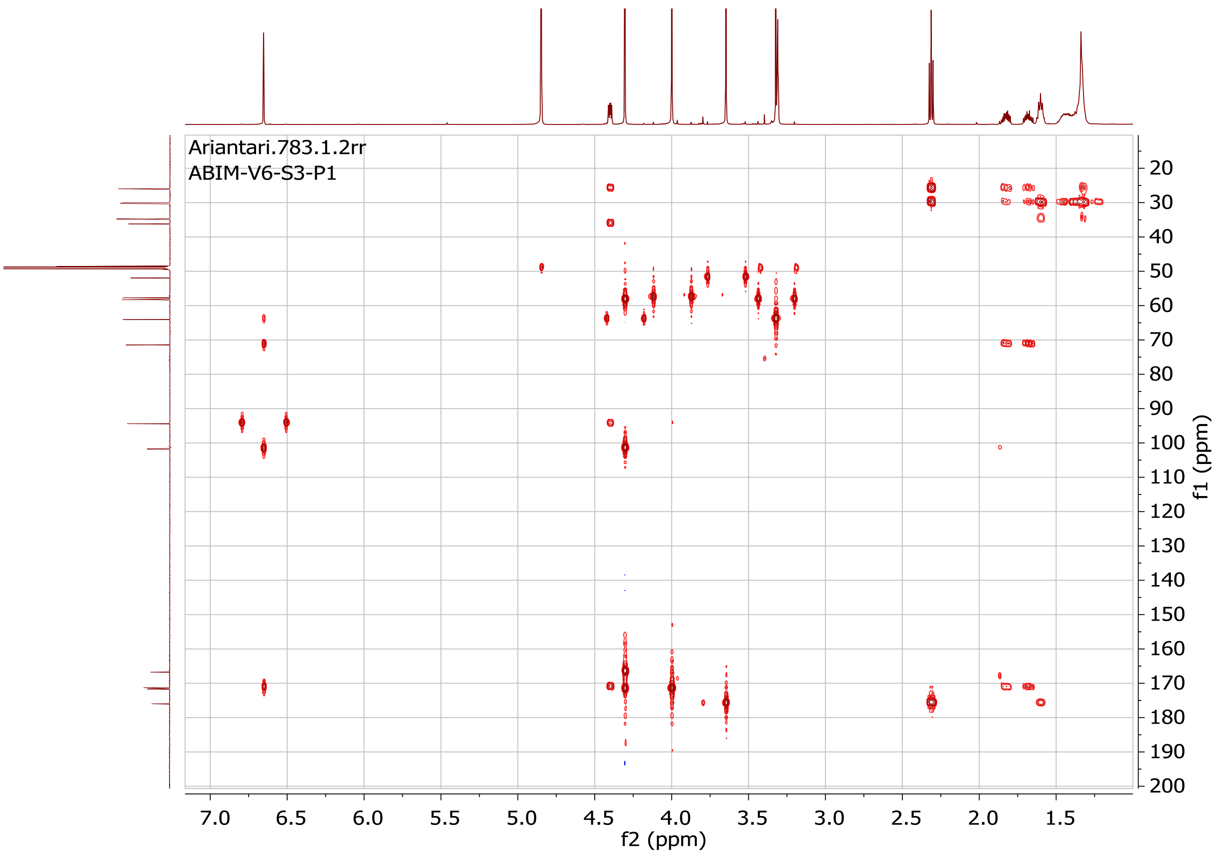

Figure S72. HMBC (600 and 150 MHz, MeOH-d_4) spectrum of compound **10**.

(A)

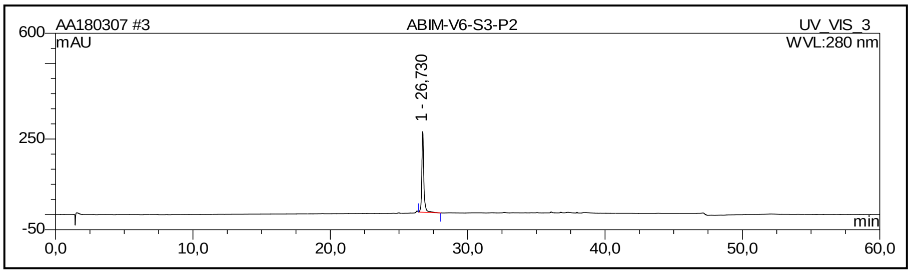

(B)

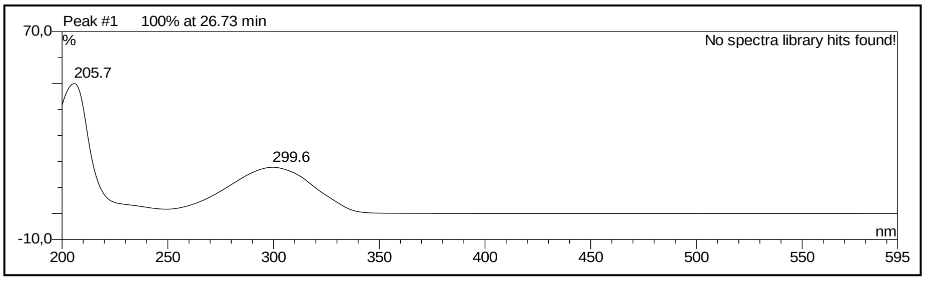

Figure S73. HPLC chromatogram (A) and UV spectrum (B) of compound **11**.

Acquisition Parameter

Source Type	ESI	Ion Polarity	Positive	Set Nebulizer	0.3 Bar
Focus	Not active	Set Capillary	4000 V	Set Dry Heater	180 °C
Scan Begin	50 m/z	Set End Plate Offset	-500 V	Set Dry Gas	4.0 l/min
Scan End	1500 m/z	Set Collision Cell RF	600.0 Vpp	Set Divert Valve	Source

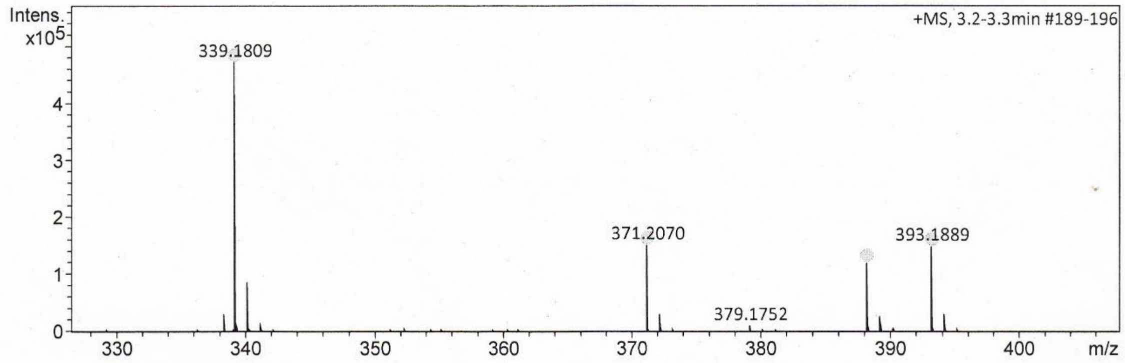

Meas. m/z	#	Ion Formula	m/z	err [ppm]	mSigma	# mSigma	Score	rdb	e⁻ Conf	N-Rule
339.1809	1	C18H27O6	339.1802	-2.0	11.6	1	100.00	5.5	even	ok
	2	C19H23N4O2	339.1816	2.0	25.1	2	75.40	10.5	even	ok
371.2070	1	C19H31O7	371.2064	-1.4	4.6	1	100.00	4.5	even	ok
388.2334	1	C19H34NO7	388.2330	-1.1	1.9	1	100.00	3.5	even	ok
393.1889	1	C19H30NaO7	393.1884	-1.3	2.0	1	100.00	4.5	even	ok
	2	C20H26N4NaO3	393.1897	2.1	15.2	2	67.19	9.5	even	ok

Figure S74. HRESIMS spectrum of compound **11**.

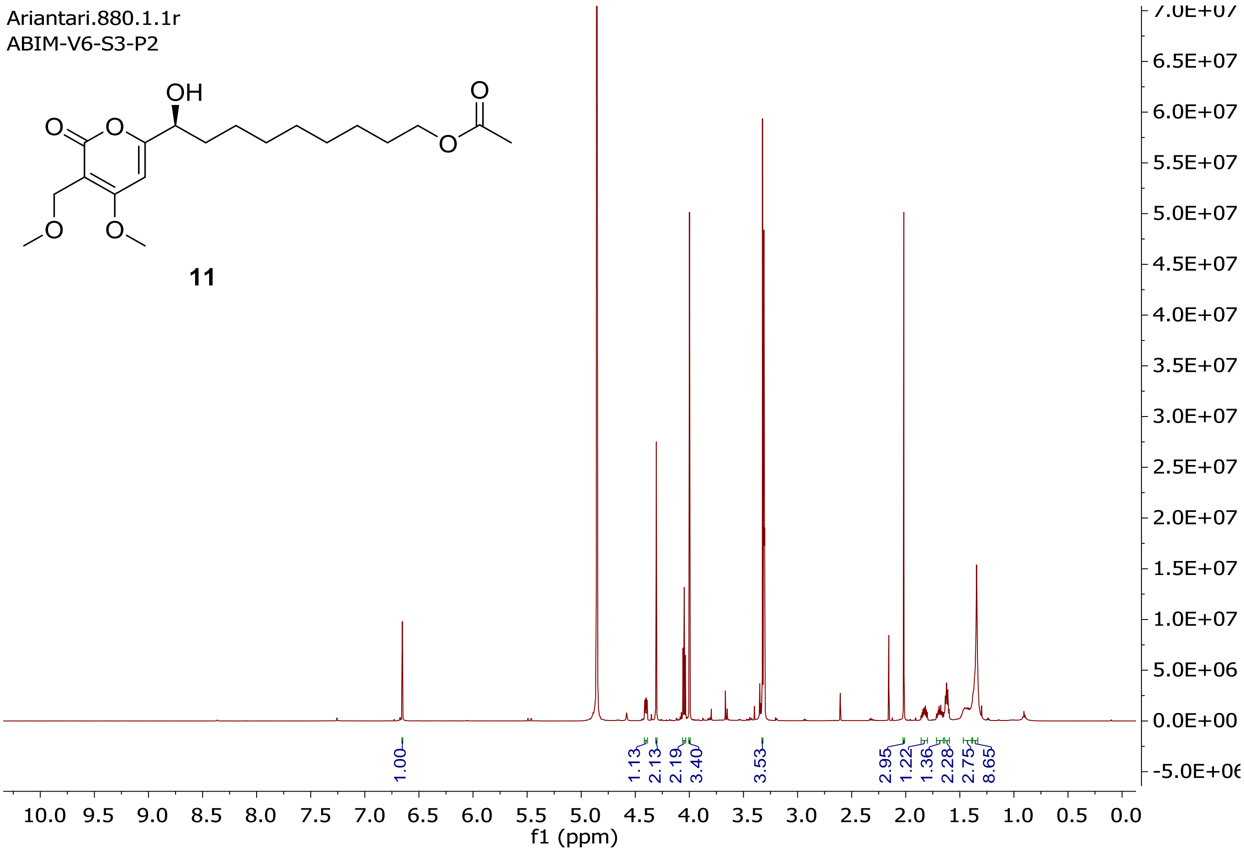

Figure S75. ^{1}H NMR (600 MHz, MeOH-d_4) spectrum of compound **11**.

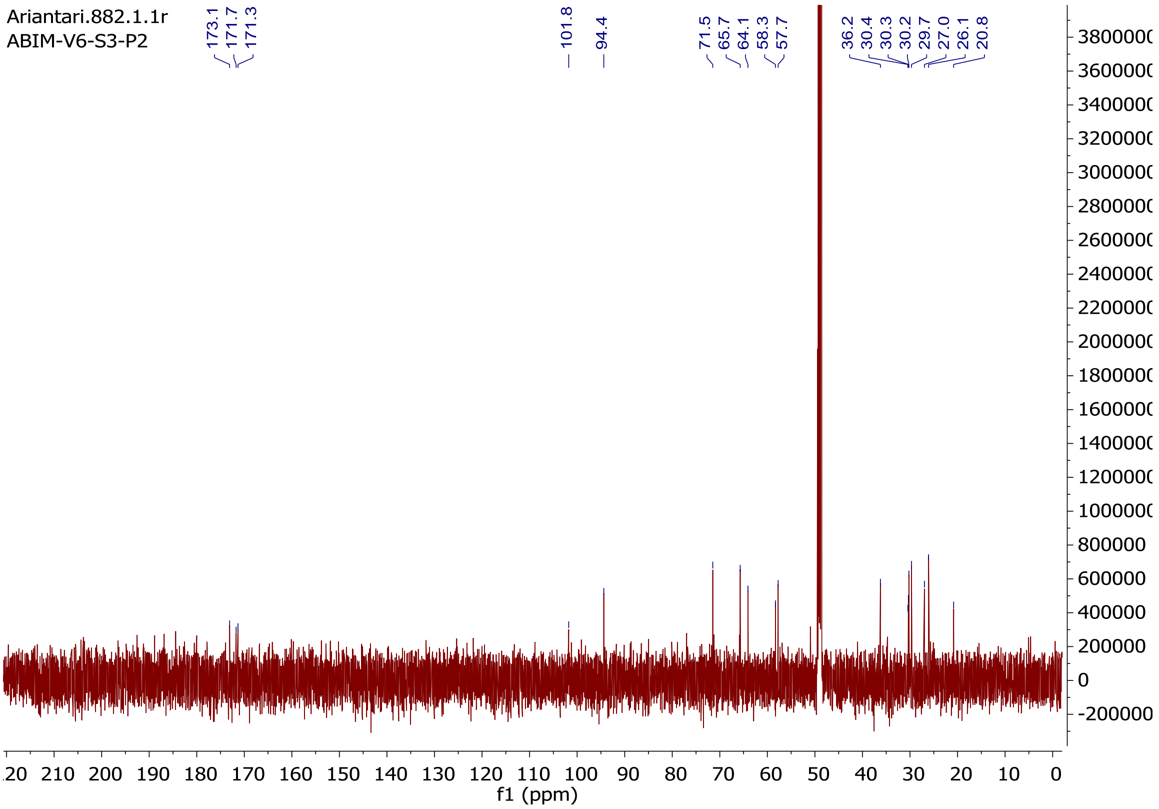

Figure S76. ^{13}C NMR (150 MHz, MeOH-d_4) spectrum of compound **11**.
Note: δ_C 166.7 was hidden in the baseline, but could be extracted from the HMBC spectrum.

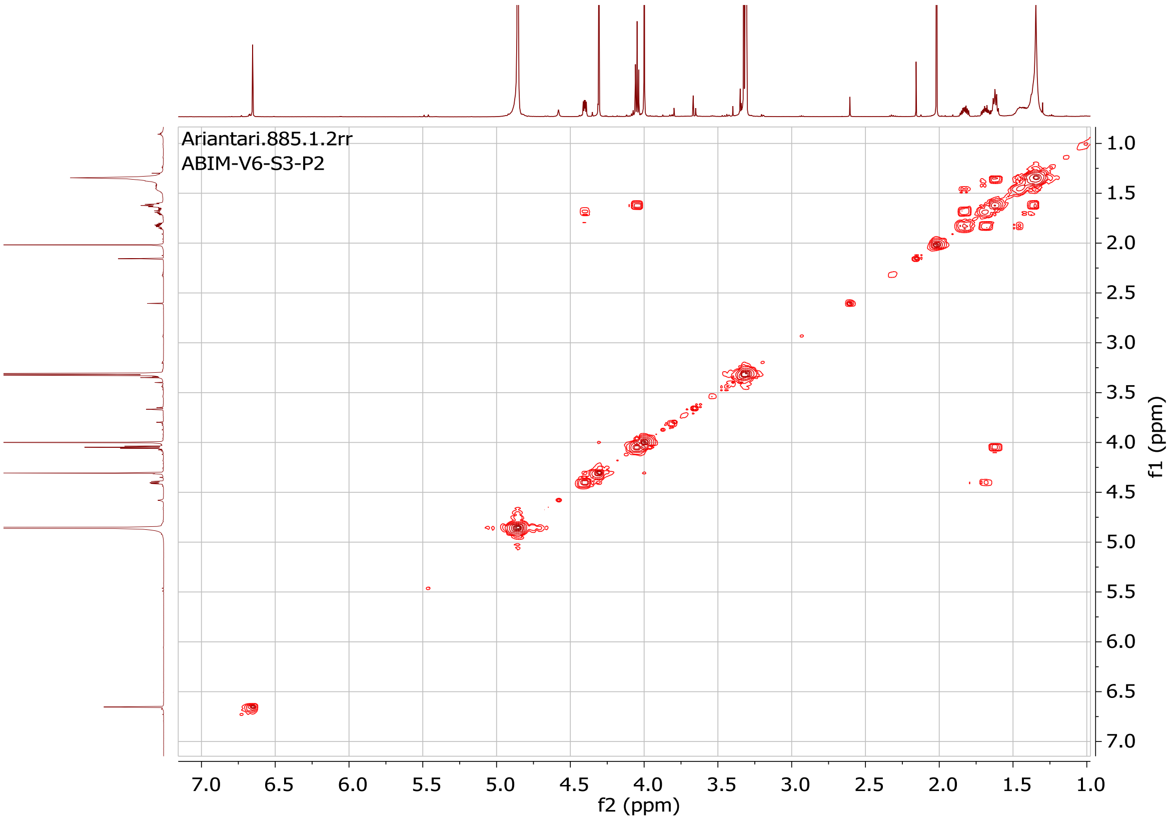

Figure S77. ^{1}H-^{1}H COSY (600 MHz, MeOH-d_4) spectrum of compound **11**.

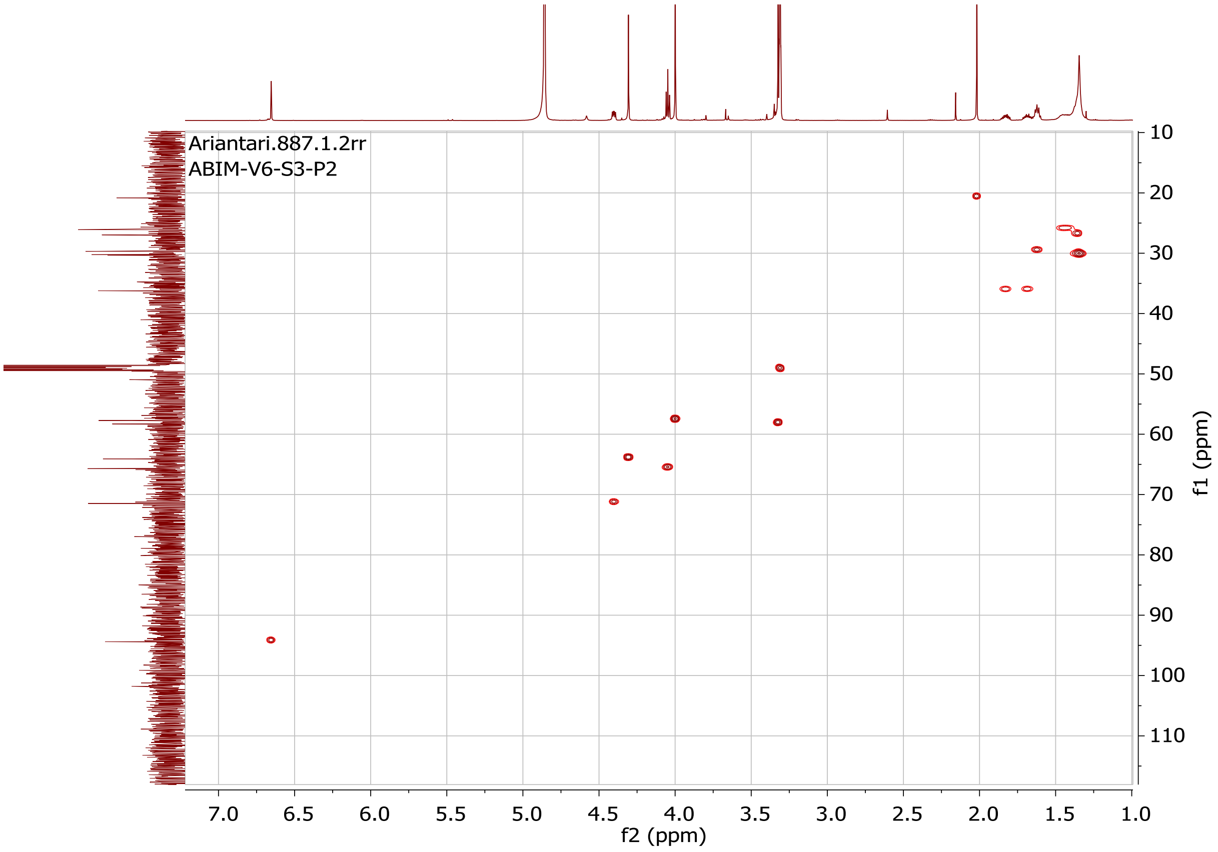

Figure S78. HSQC (600 and 150 MHz, MeOH-d_4) spectrum of compound **11**.

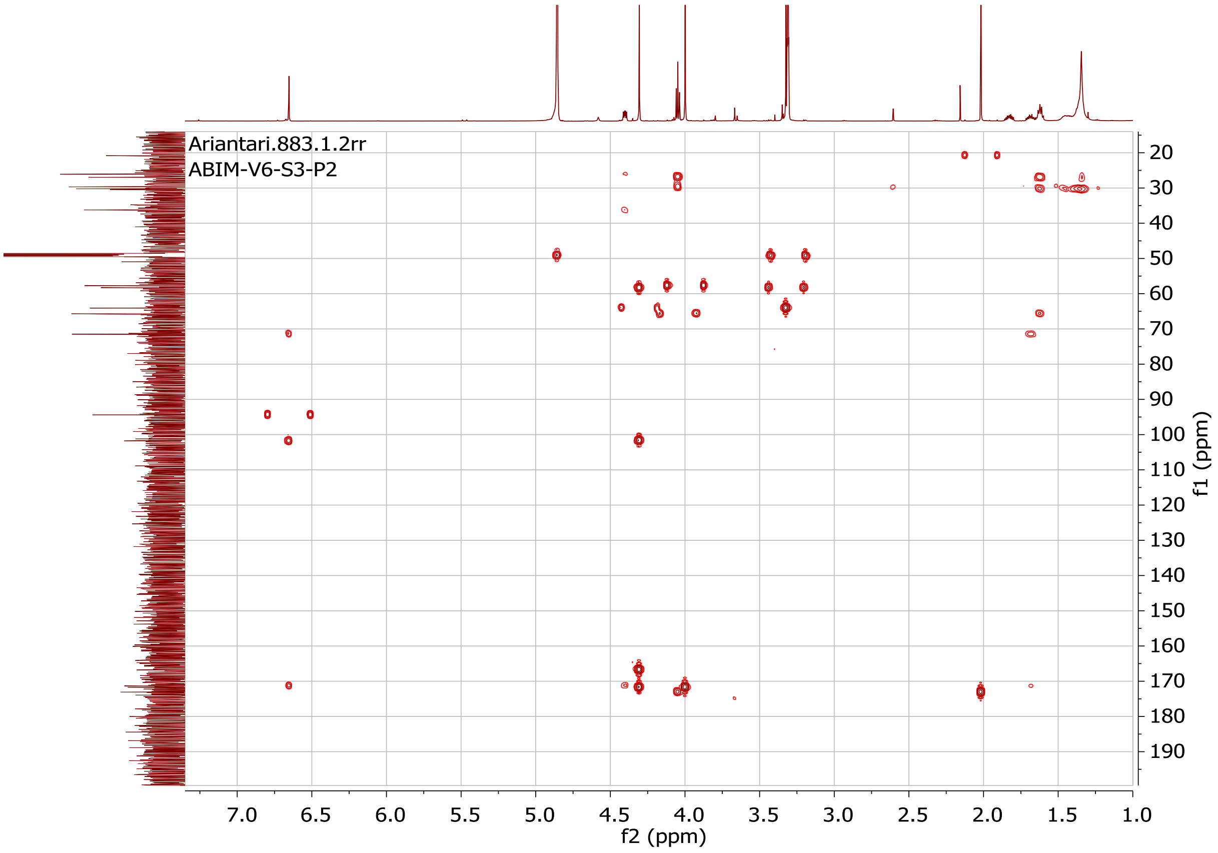

Figure S79. HMBC (600 and 150 MHz, MeOH-d_4) spectrum of compound **11**.

(A)

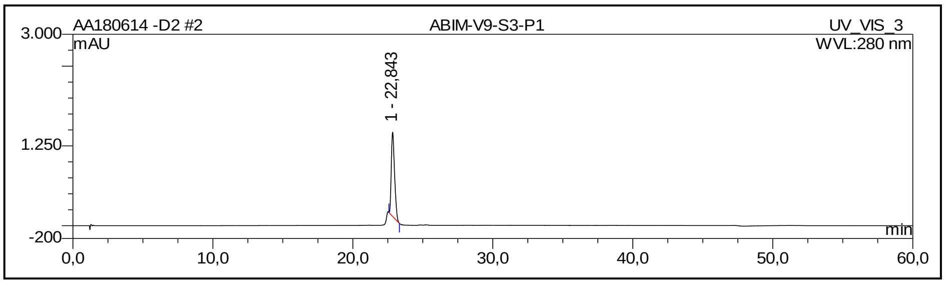

(B)

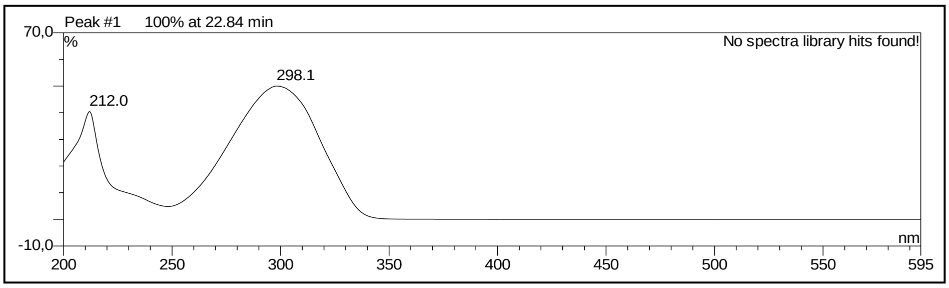

Figure S80. HPLC chromatogram (A) and UV spectrum (B) of compound **12**.

Acquisition Parameter

Source Type	ESI	Ion Polarity	Positive	Set Nebulizer	0.3 Bar
Focus	Not active	Set Capillary	4000 V	Set Dry Heater	180 °C
Scan Begin	50 m/z	Set End Plate Offset	-500 V	Set Dry Gas	4.0 l/min
Scan End	1500 m/z	Set Collision Cell RF	600.0 Vpp	Set Divert Valve	Source

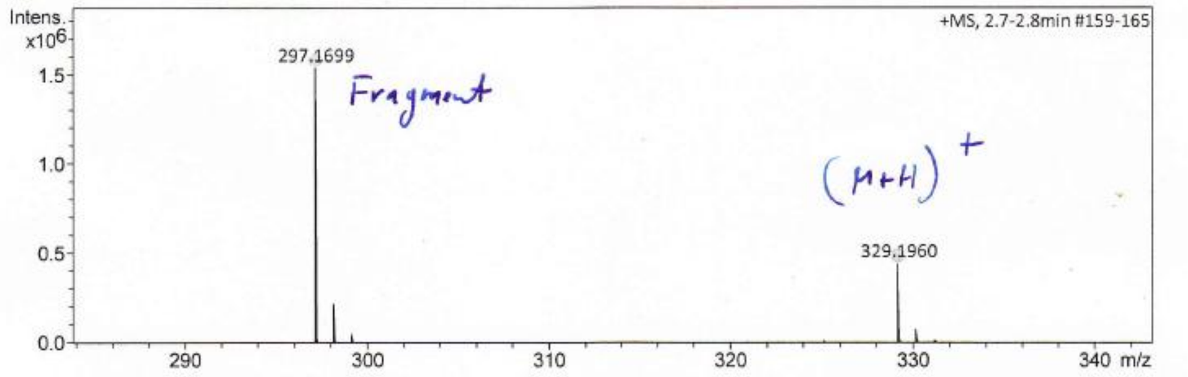

Meas. m/z	#	Ion Formula	m/z	err [ppm]	mSigma	# mSigma	Score	rdb	e⁻ Conf	N-Rule
297.1699	1	C16H25O5	297.1697	-0.8	22.6	1	100.00	4.5	even	ok
329.1960	1	C17H29O6	329.1959	-0.4	11.1	1	100.00	3.5	even	ok

Figure S81. HRESIMS spectrum of compound **12**.

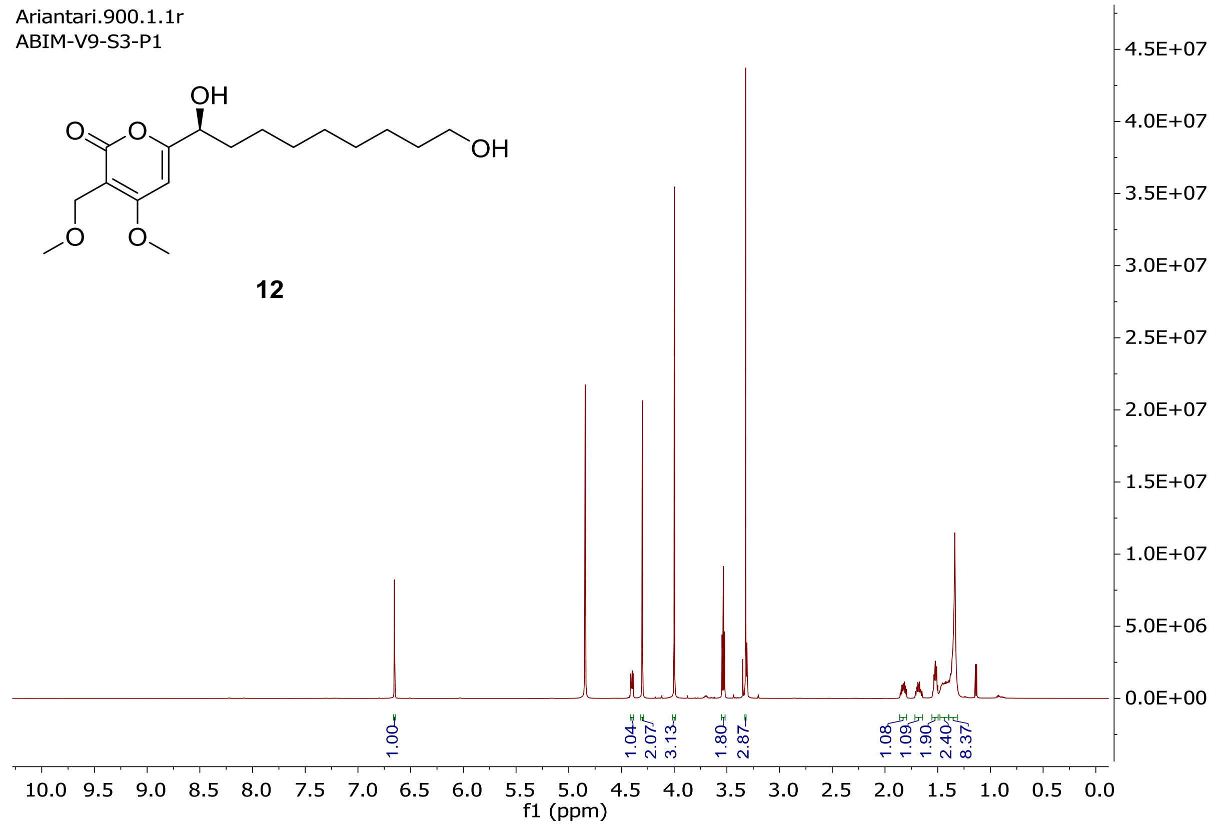

Figure S82. ^{1}H NMR (600 MHz, MeOH-d_4) spectrum of compound **12**.

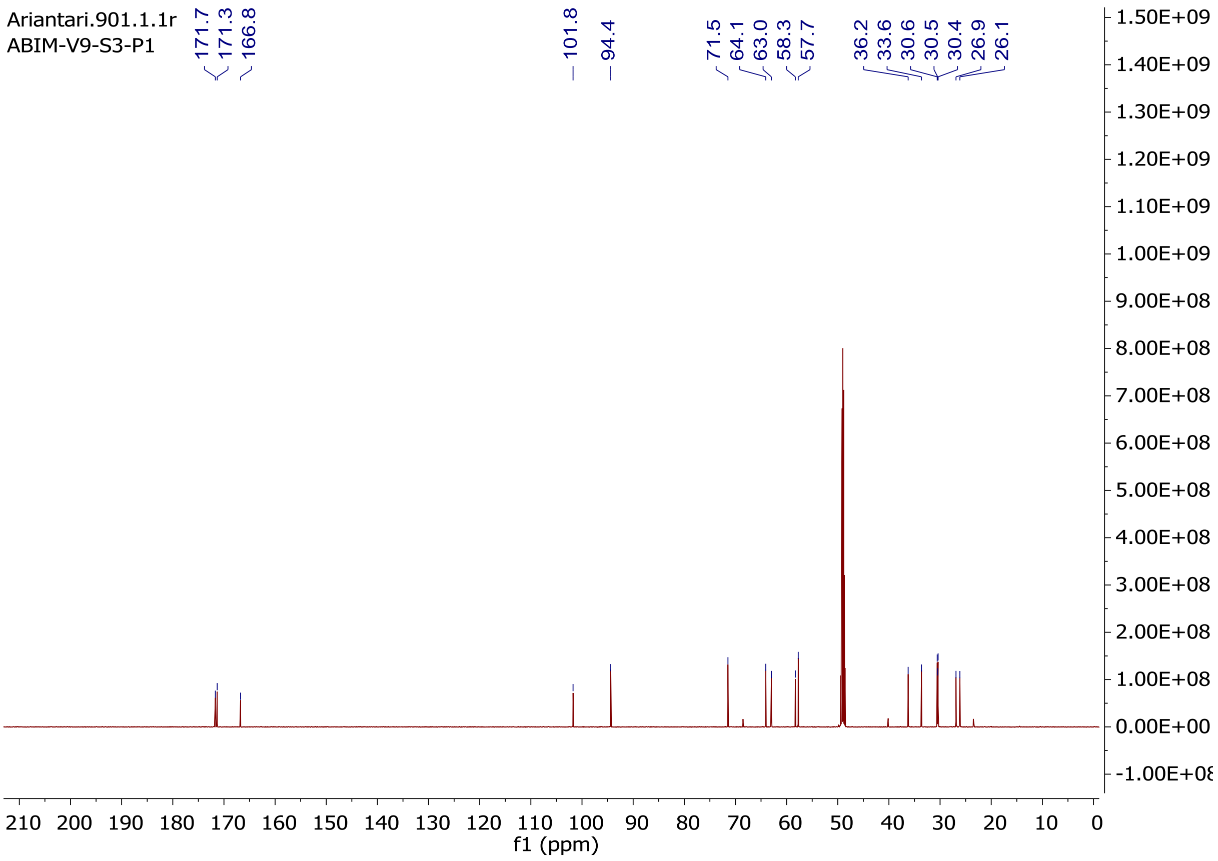

Figure S83. ^{13}C NMR (150 MHz, MeOH-d_4) spectrum of compound **12**.

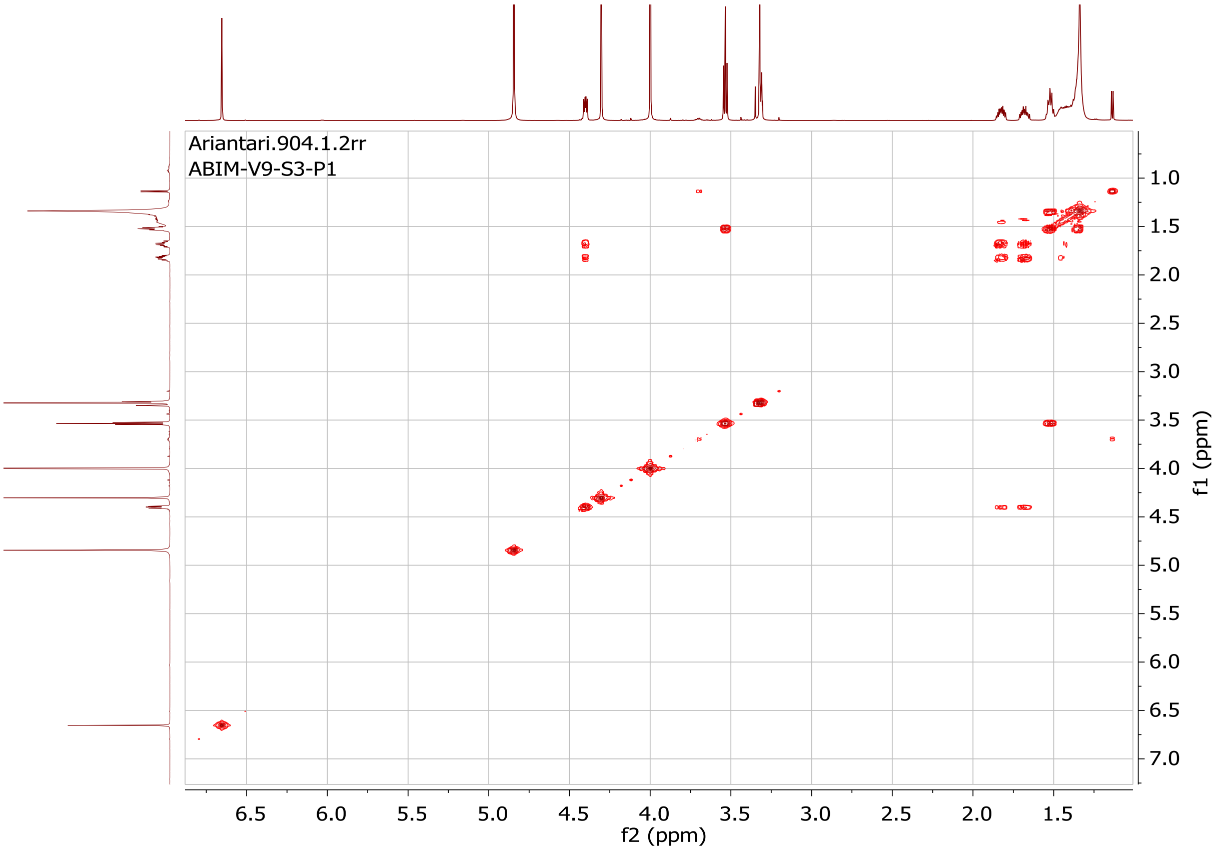

Figure S84. ^{1}H-^{1}H COSY (600 MHz, MeOH-d_4) spectrum of compound **12**.

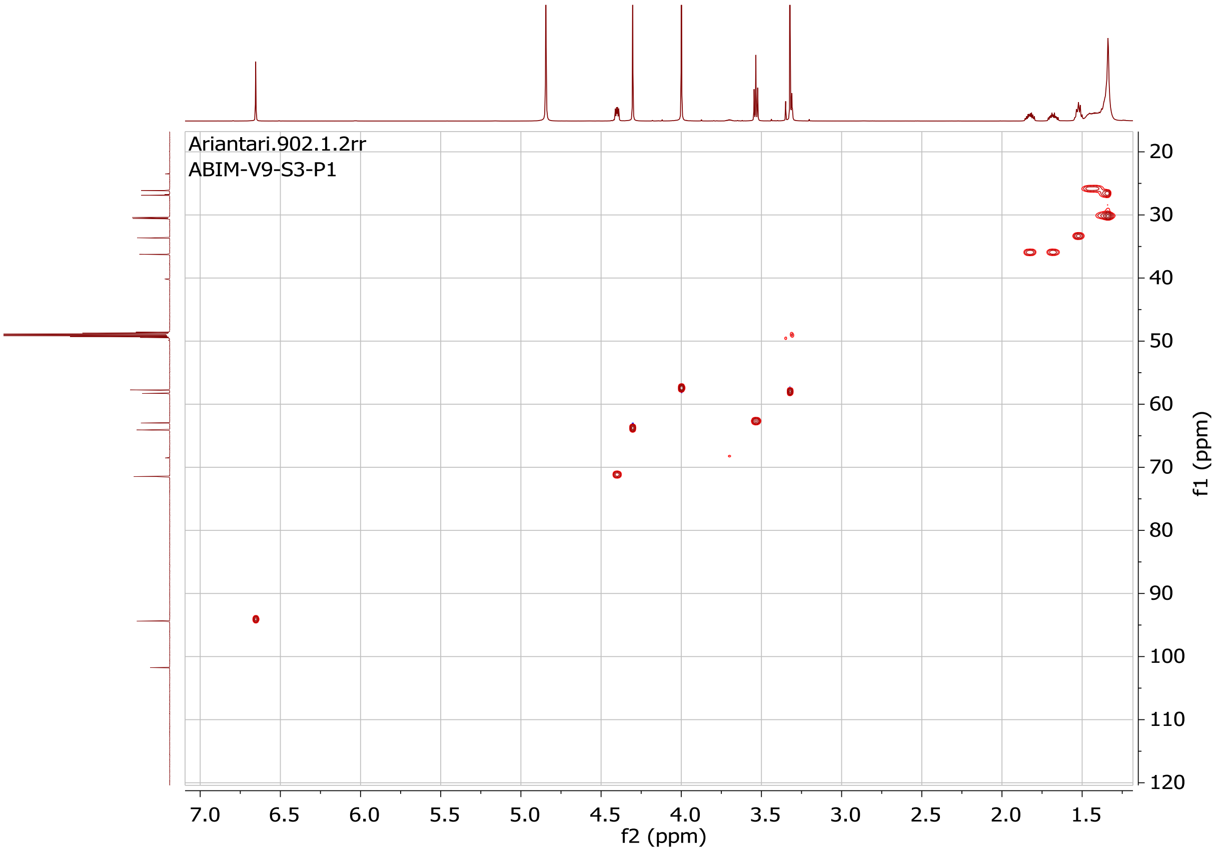

Figure S85. HSQC (600 and 150 MHz, MeOH-d_4) spectrum of compound **12**.

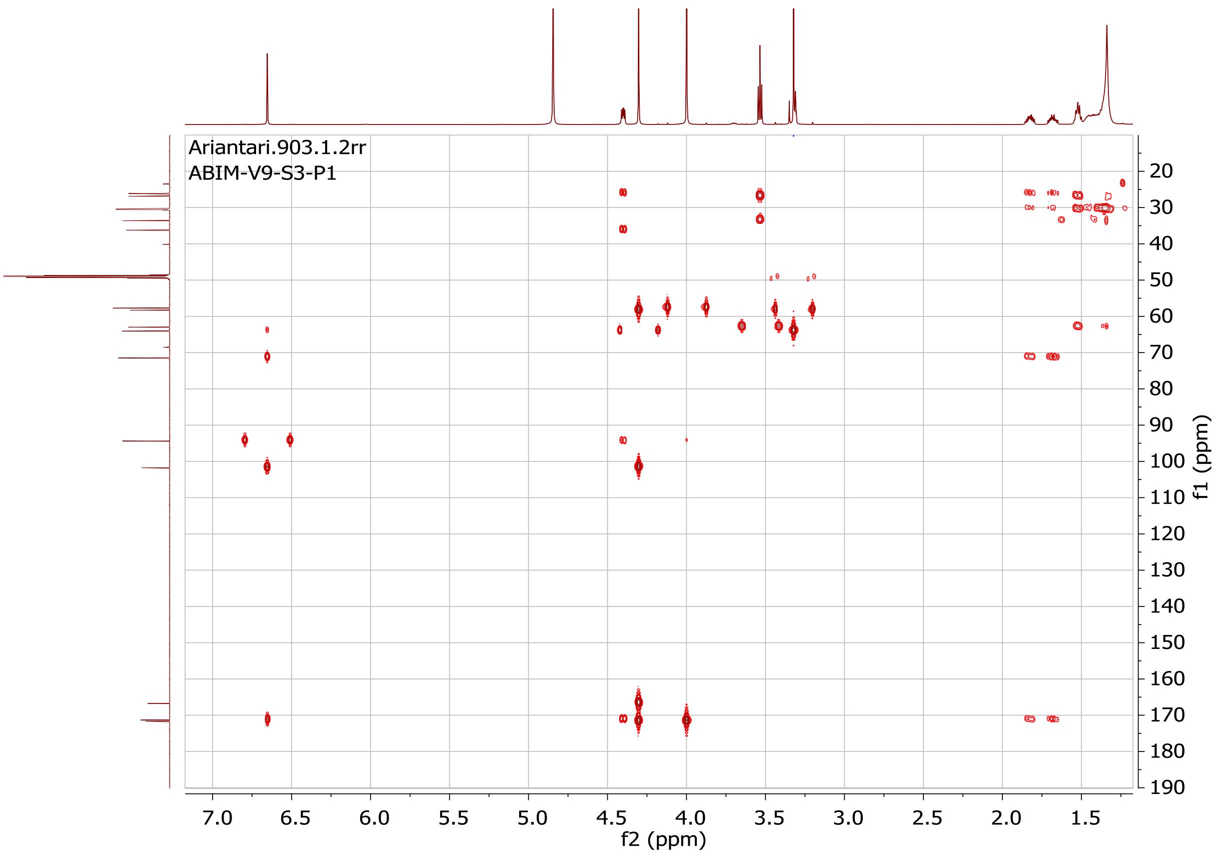

Figure S86. HMBC (600 and 150 MHz, MeOH-d_4) spectrum of compound **12**.

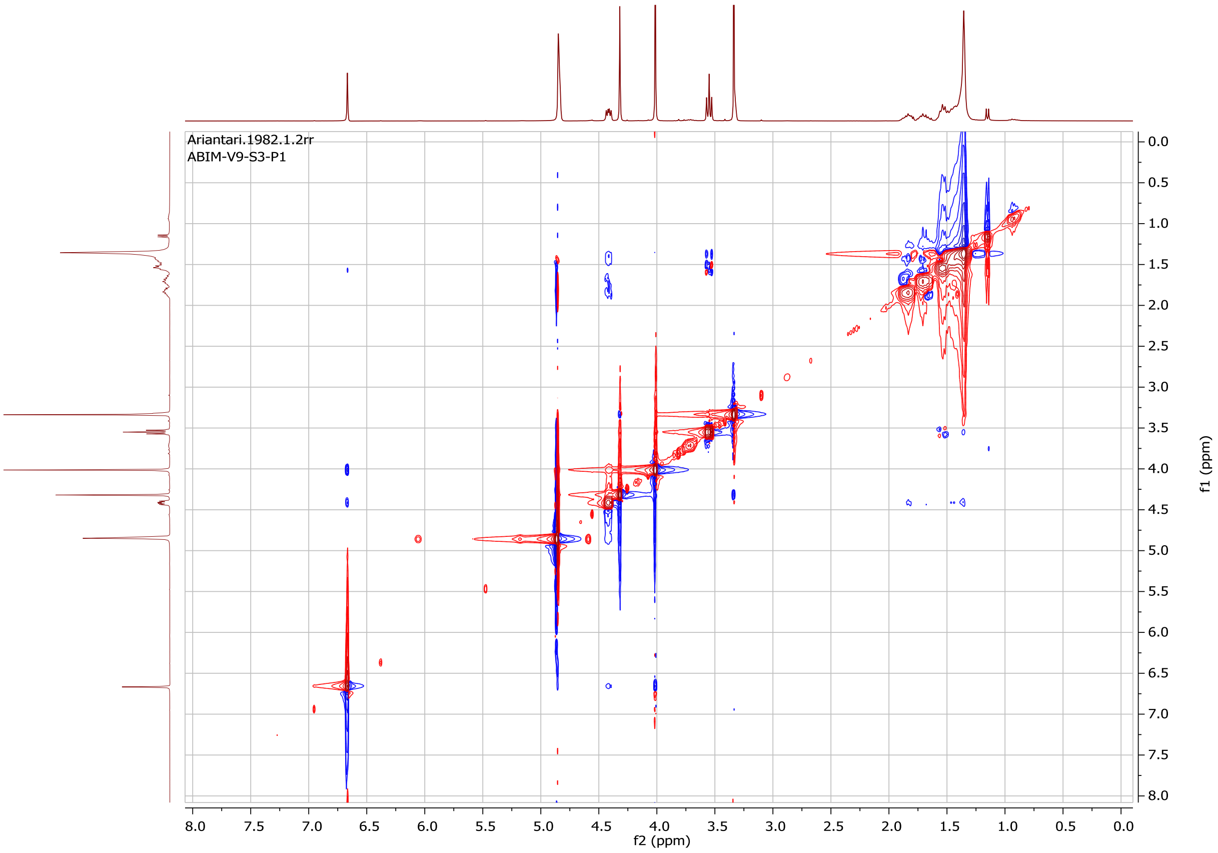

Figure S87. NOESY (300 MHz, MeOH-d_4) spectrum of compound **12**.

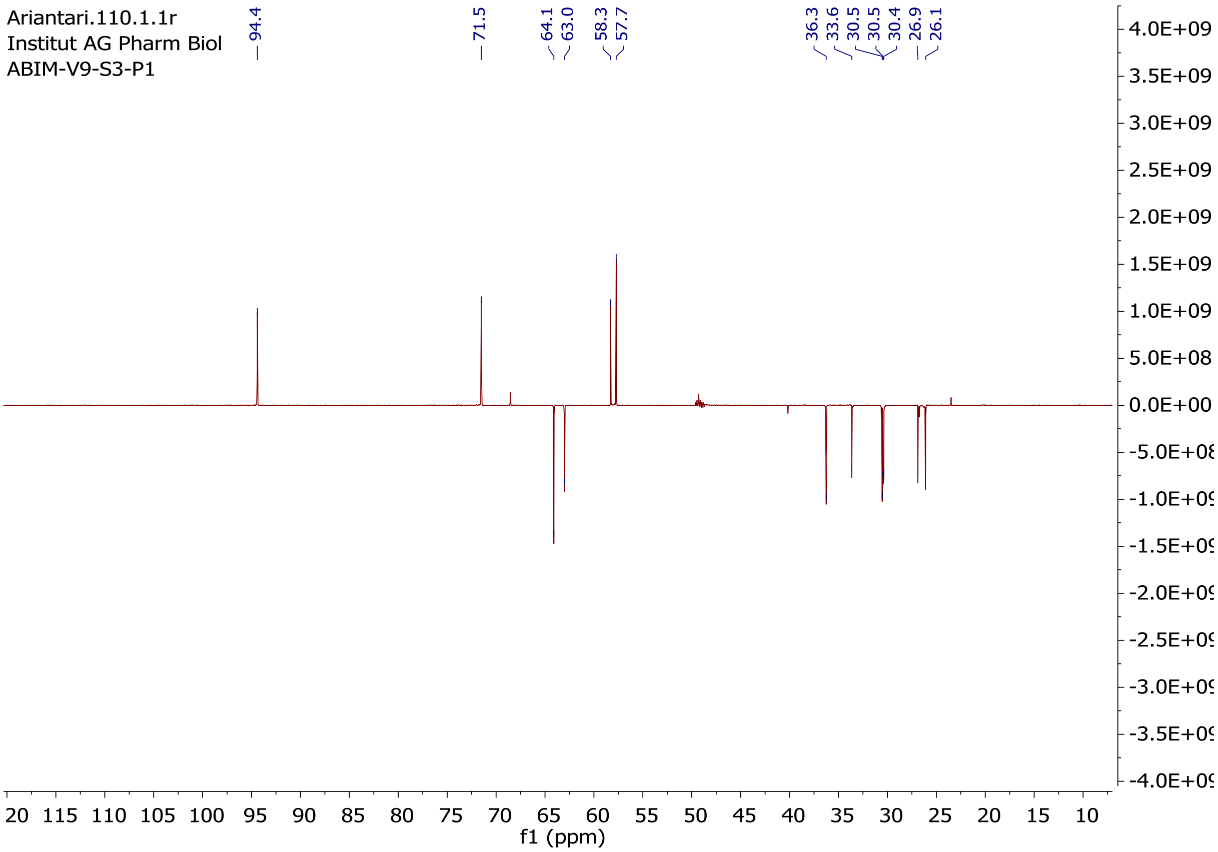

Figure S88. DEPT 135 (125 MHz, MeOH-d_4) spectrum of compound **12**.

(A)

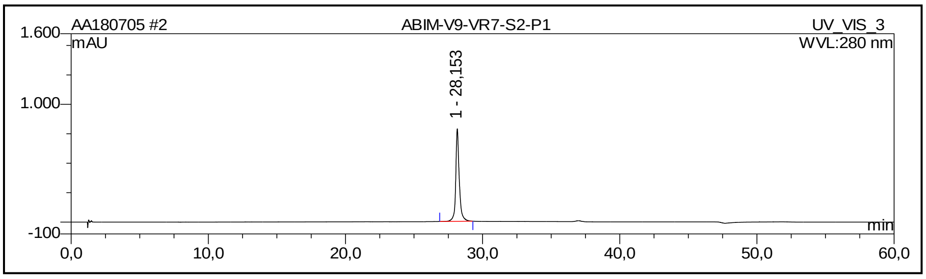

(B)

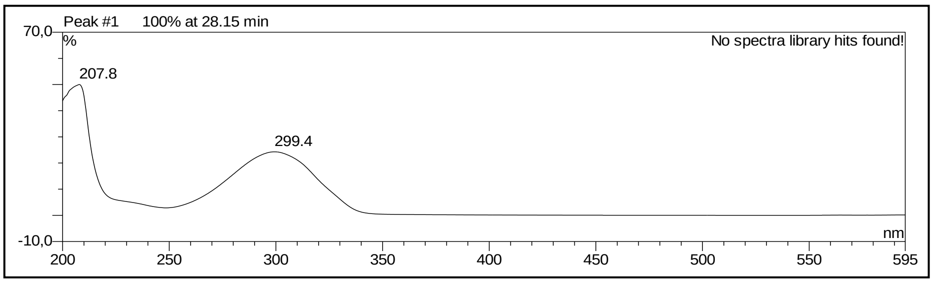

Figure S89. HPLC chromatogram (A) and UV spectrum (B) of compound **13**.

Acquisition Parameter

Source Type	ESI	Ion Polarity	Positive	Set Nebulizer	0.3 Bar
Focus	Not active	Set Capillary	4000 V	Set Dry Heater	180 °C
Scan Begin	50 m/z	Set End Plate Offset	-500 V	Set Dry Gas	4.0 l/min
Scan End	1500 m/z	Set Collision Cell RF	600.0 Vpp	Set Divert Valve	Source

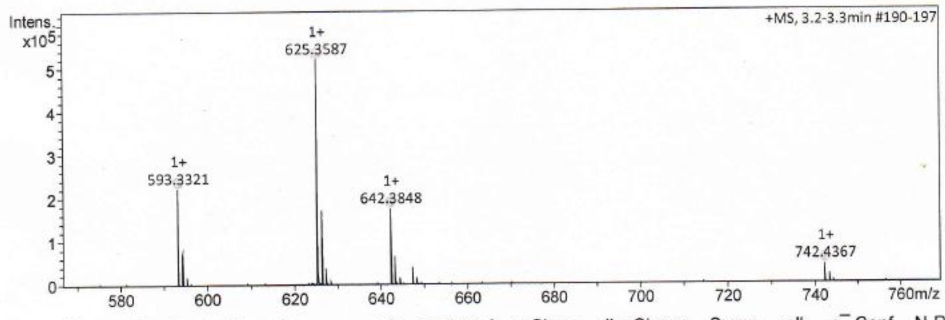

Meas. m/z	#	Ion Formula	m/z	err [ppm]	mSigma	# mSigma	Score	rdb	e⁻ Conf	N-Rule
297.1696	1	C16H25O5	297.1697	0.2	3.9	1	100.00	4.5	even	ok
329.1958	1	C17H29O6	329.1959	0.3	17.1	1	100.00	3.5	even	ok
371.3155	1	C22H43O4	371.3156	0.2	21.4	1	100.00	1.5	even	ok
388.3418	1	C22H46NO4	388.3421	0.9	23.2	1	100.00	0.5	even	ok
419.3154	1	C26H43O4	419.3156	0.5	2.6	1	100.00	5.5	even	ok
593.3321	1	C32H49O10	593.3320	-0.2	7.0	1	100.00	8.5	even	ok
	2	C33H45N4O6	593.3334	2.1	18.7	2	44.83	13.5	even	ok
	3	C30H37N14	593.3320	-0.2	20.3	3	76.73	19.5	even	ok
625.3587	1	C33H53O11	625.3582	-0.7	19.9	1	100.00	7.5	even	ok
	2	C31H41N14O	625.3582	-0.7	31.0	2	77.04	18.5	even	ok
	3	C34H49N4O7	625.3596	1.5	31.3	3	59.82	12.5	even	ok
	4	C46H45N2	625.3577	-1.5	93.9	4	6.71	25.5	even	ok
642.3848	1	C33H56NO11	642.3848	-0.1	3.6	1	100.00	6.5	even	ok
	2	C34H52N5O7	642.3861	2.0	8.9	2	47.50	11.5	even	ok
	3	C31H44N15O	642.3848	-0.1	12.2	3	85.16	17.5	even	ok
	4	C46H48N3	642.3843	-0.9	70.8	4	13.24	24.5	even	ok
742.4367	1	C36H52N15O3	742.4372	0.7	10.0	1	100.00	18.5	even	ok
	2	C38H64NO13	742.4372	0.7	14.2	2	91.91	7.5	even	ok
	3	C35H56N11O7	742.4359	-1.1	16.8	3	77.02	13.5	even	ok
	4	C51H56N3O2	742.4367	0.1	61.3	4	32.79	25.5	even	ok

Figure S90. HRESIMS spectrum of compound **13**.

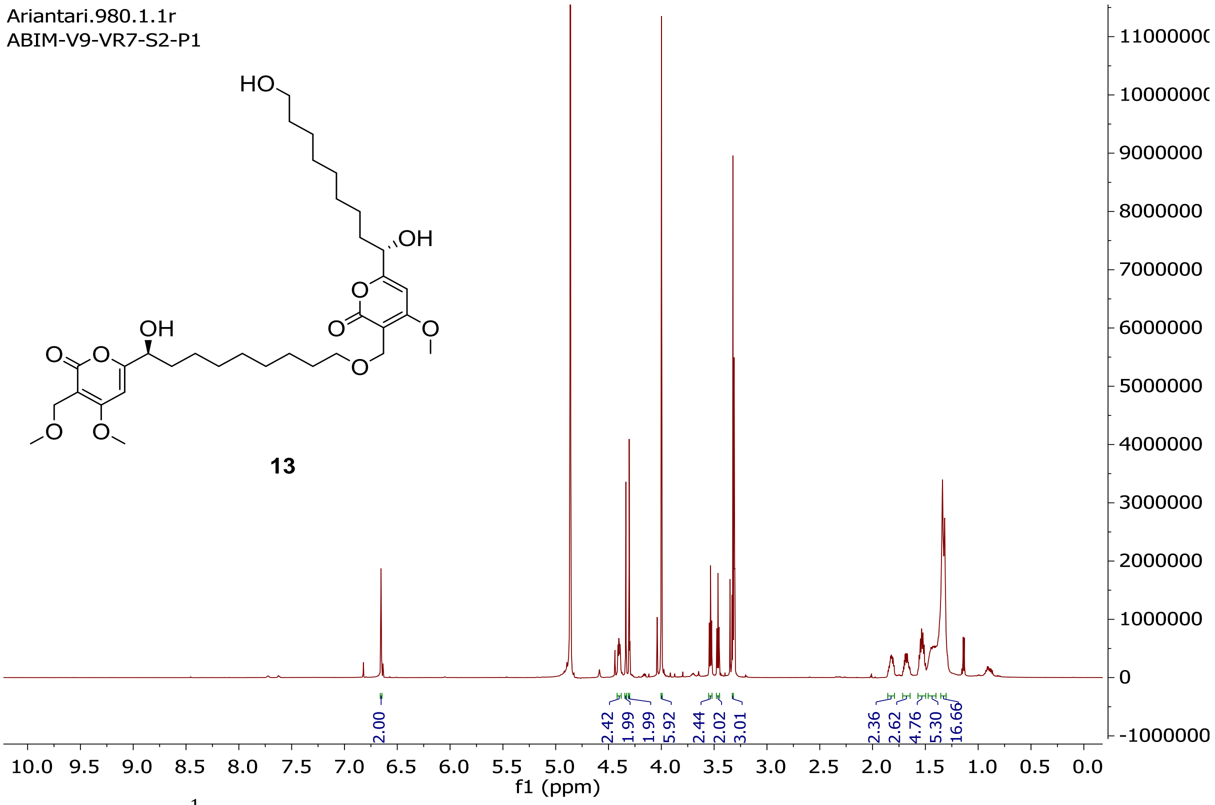

Figure S91. ^{1}H NMR (600 MHz, MeOH-d_4) spectrum of compound **13**.

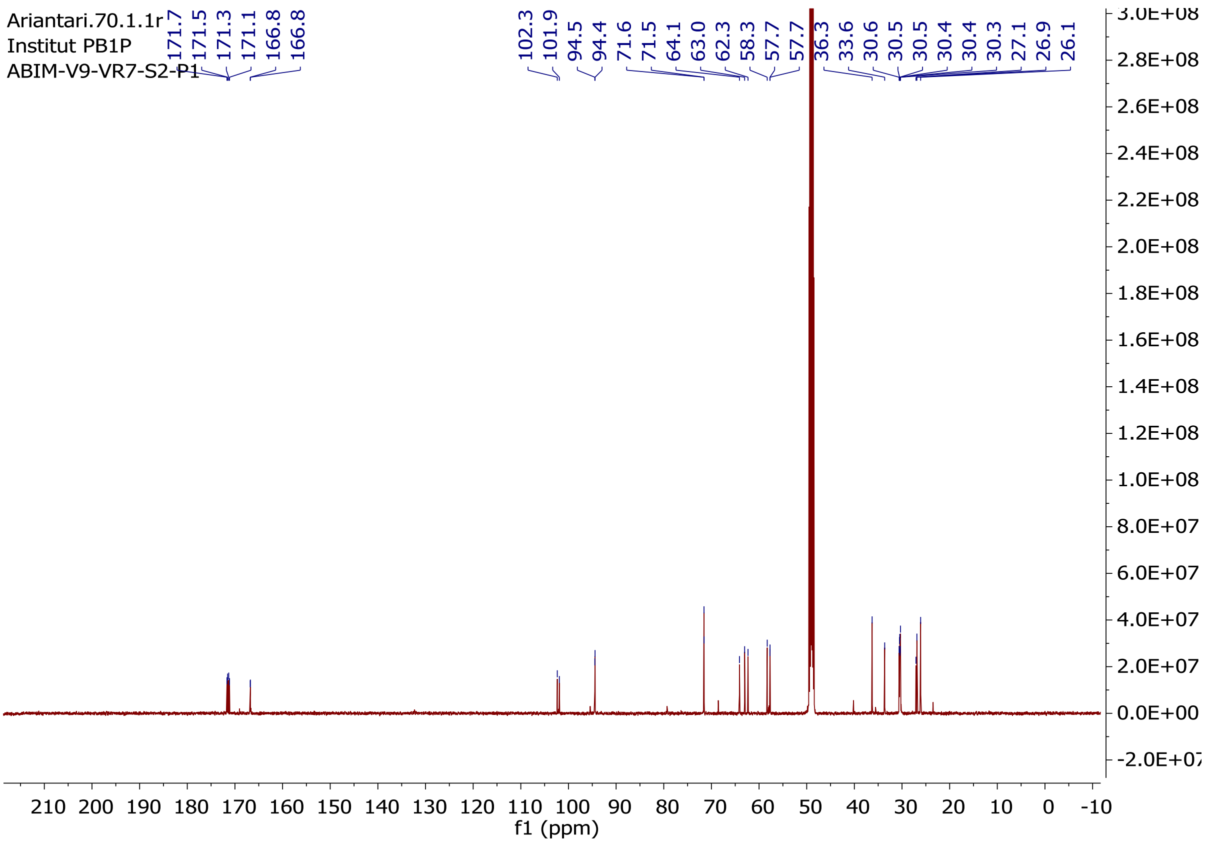

Figure S92. ^{13}C NMR (125 MHz, MeOH-d_4) spectrum of compound **13**.

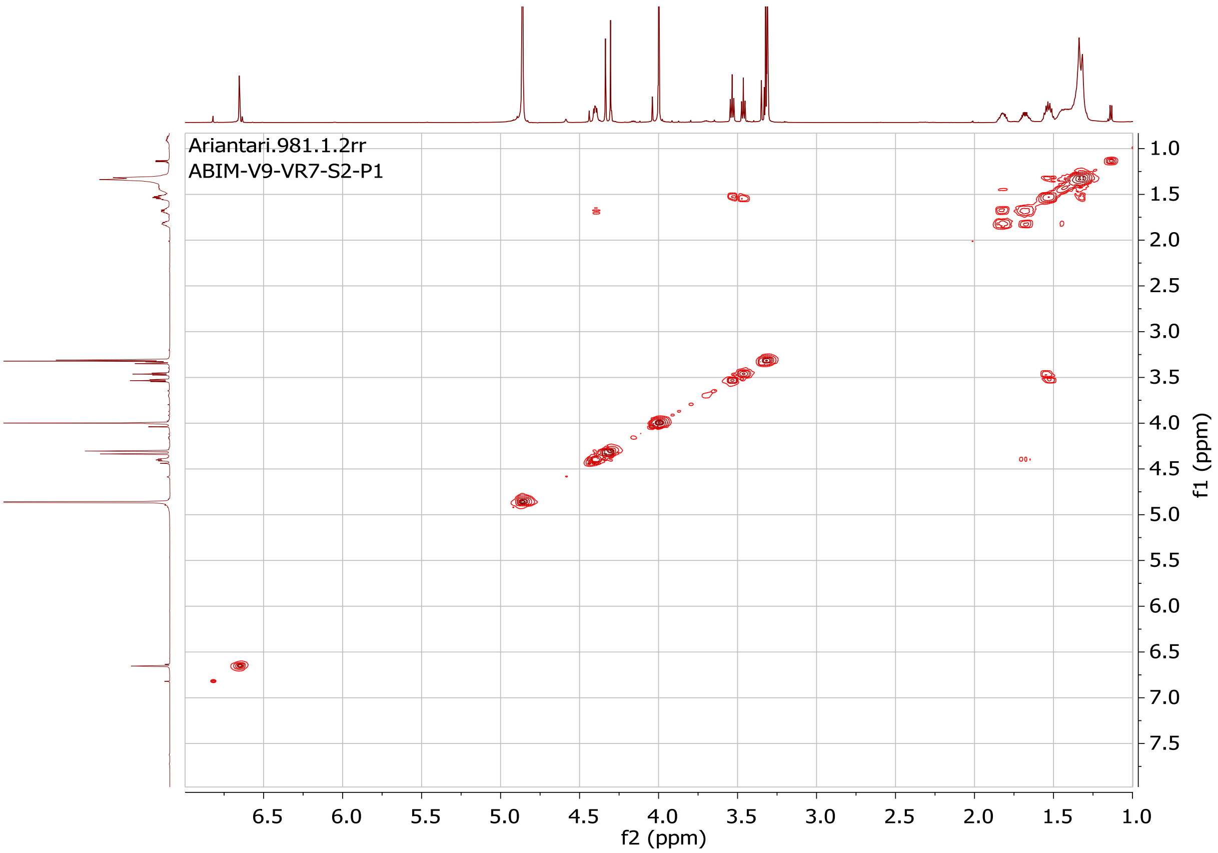

Figure S93. ^{1}H-^{1}H COSY (600 MHz, MeOH-d_4) spectrum of compound **13**.

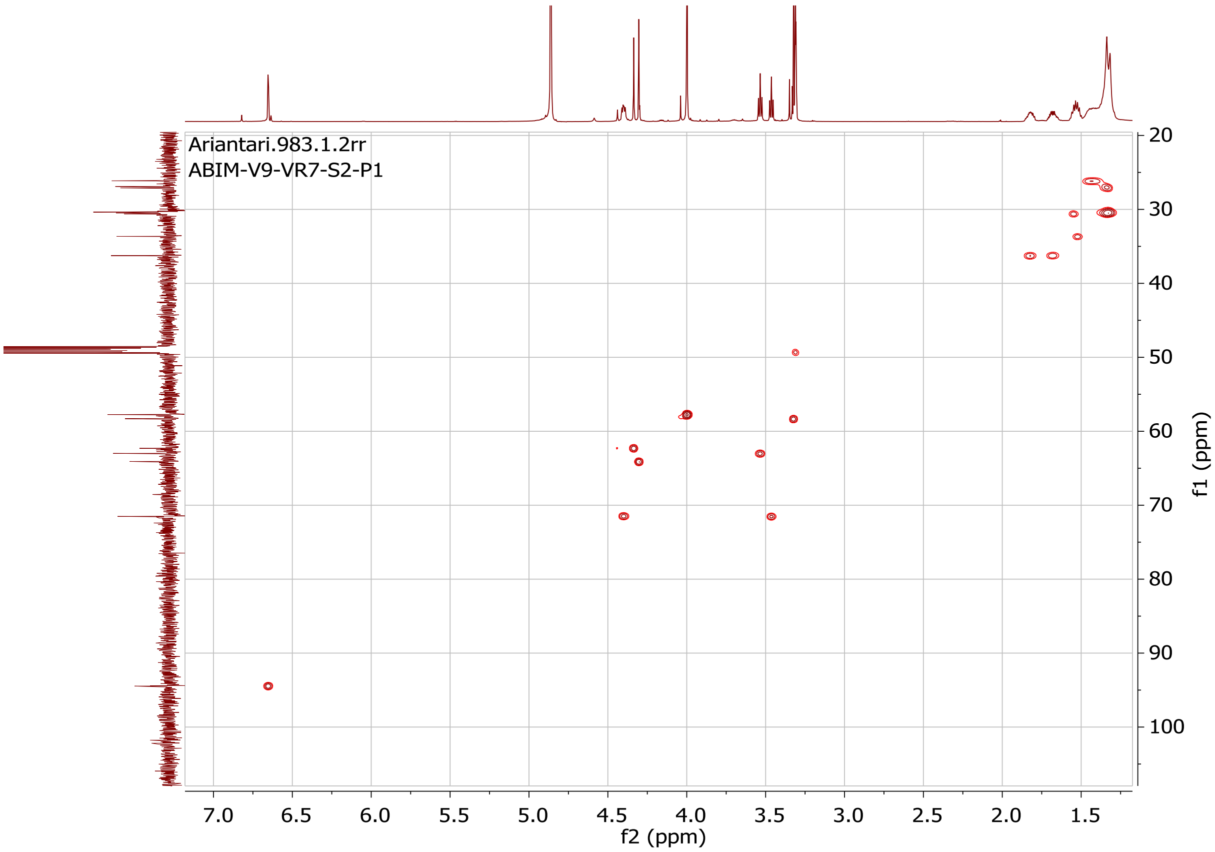

Figure S94. HSQC (600 and 150 MHz, MeOH-d_4) spectrum of compound **13**.

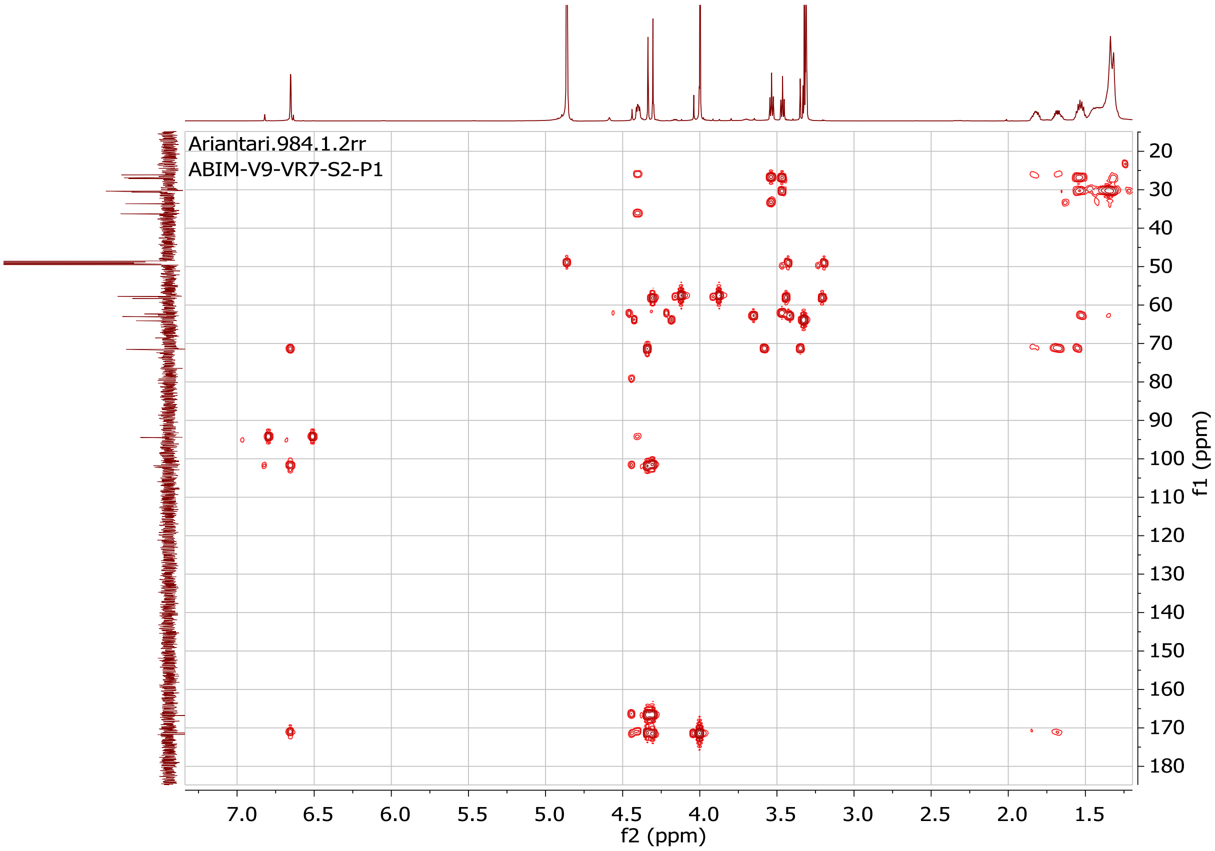

Figure S95. HMBC (600 and 150 MHz, MeOH-d_4) spectrum of compound **13**.

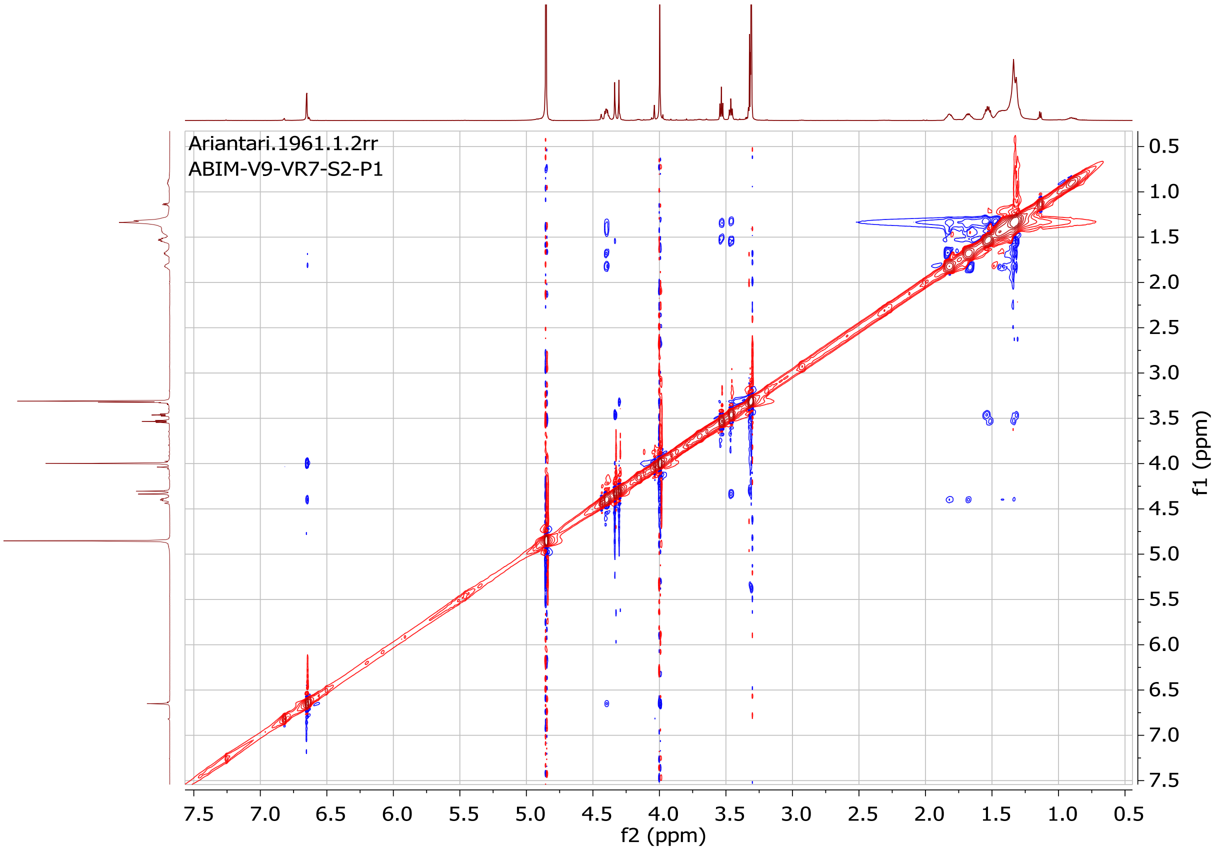

Figure S96. NOESY (600 MHz, MeOH-d_4) spectrum of compound **13**.

(A)

(B)

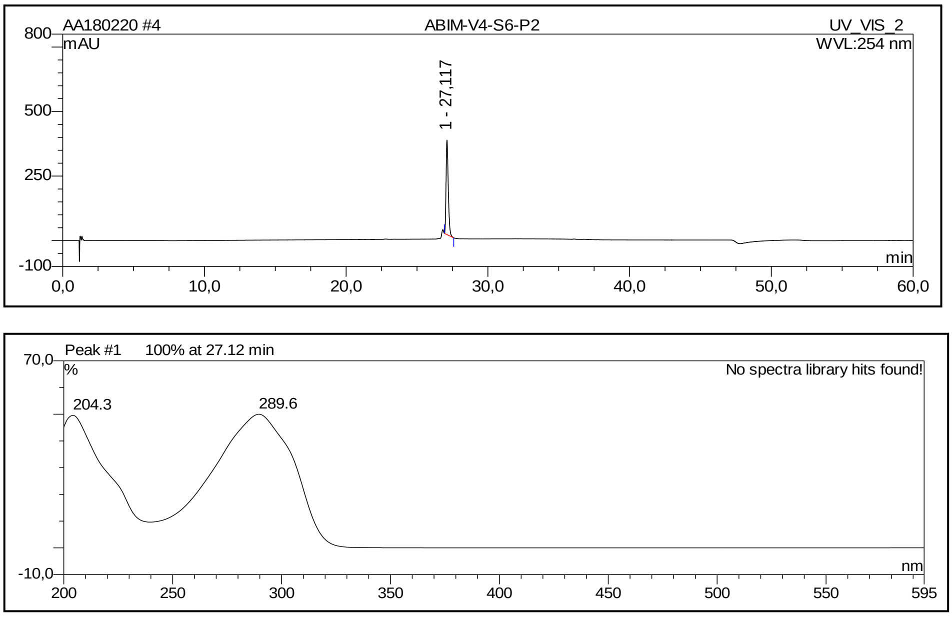

Figure S97. HPLC chromatogram (A) and UV spectrum (B) of compound **16**.

Acquisition Parameter

Source Type	ESI	Ion Polarity	Positive	Set Nebulizer	0.3 Bar
Focus	Not active	Set Capillary	4000 V	Set Dry Heater	180 °C
Scan Begin	50 m/z	Set End Plate Offset	-500 V	Set Dry Gas	4.0 l/min
Scan End	1500 m/z	Set Collision Cell RF	600.0 Vpp	Set Divert Valve	Source

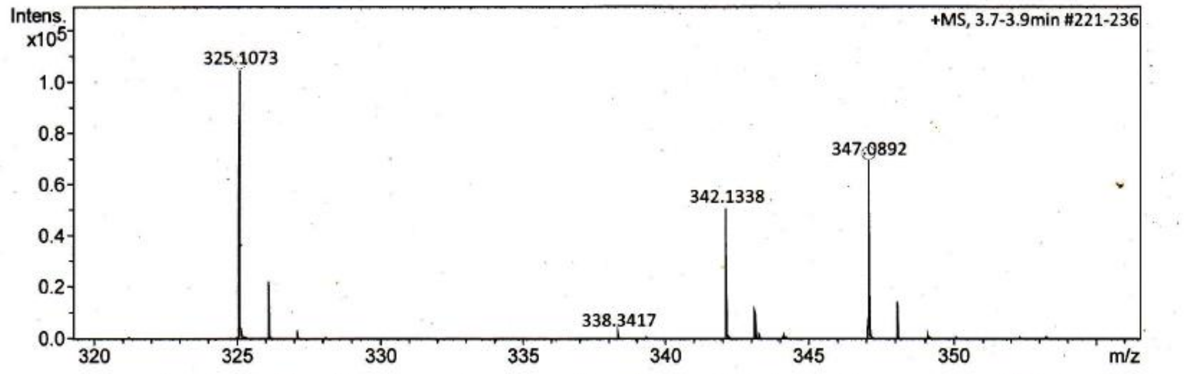

Meas. m/z	#	Ion Formula	m/z	err [ppm]	mSigma	# mSigma	Score	rdb	e⁻ Conf	N-Rule
325.1073	1	C19H17O5	325.1071	-0.8	3.2	1	100.00	11.5	even	ok
347.0892	1	C19H16NaO5	347.0890	-0.6	1.3	1	100.00	11.5	even	ok

Figure S98. HRESIMS spectrum of compound **16**.

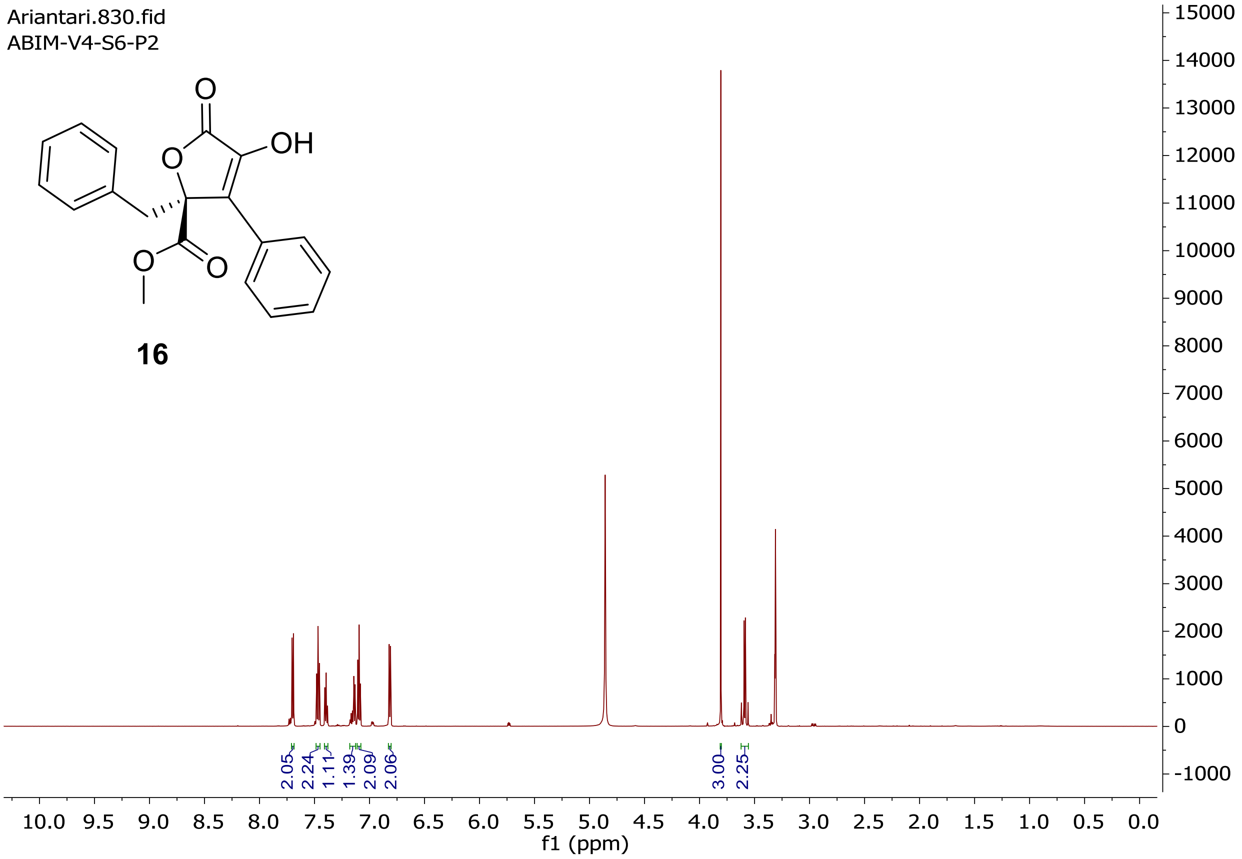

Figure S99. ^{1}H NMR (600 MHz, MeOH-d_4) spectrum of compound **16**.

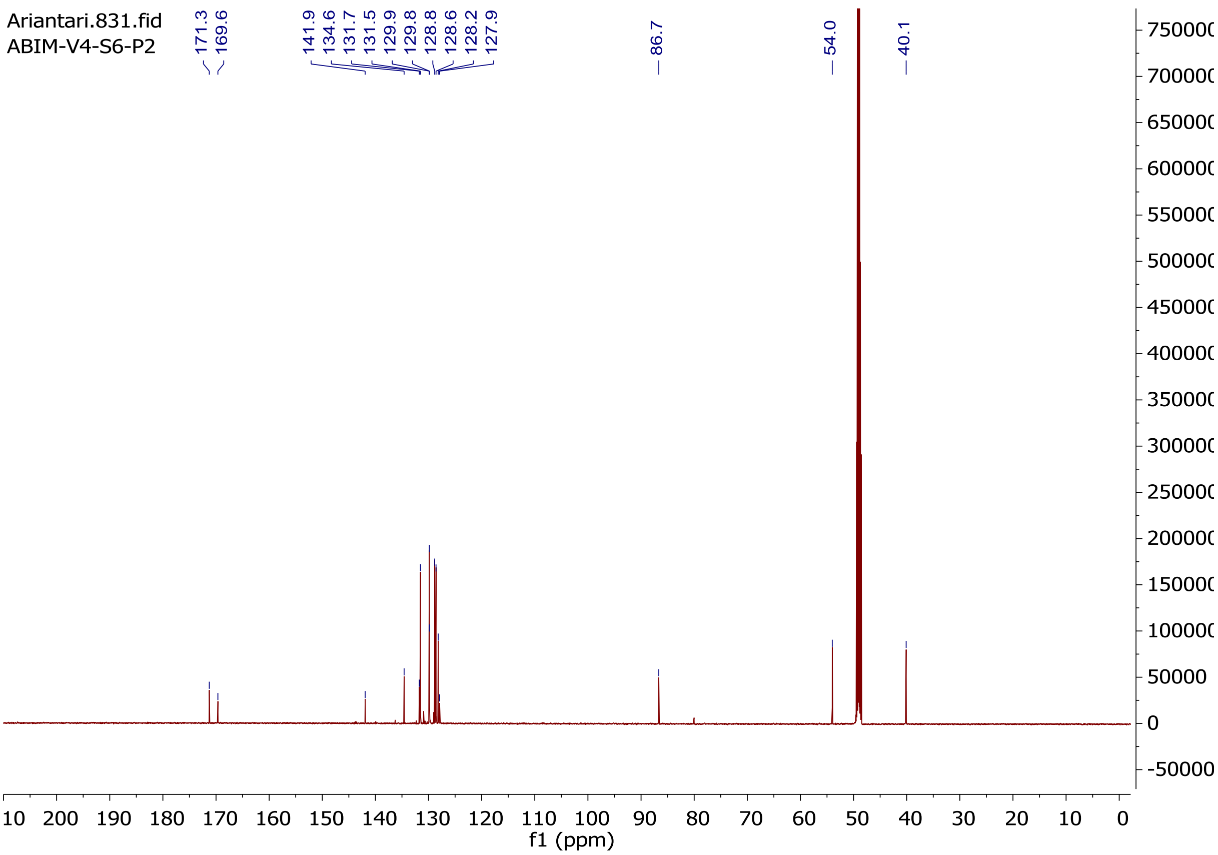

Figure S100. ^{13}C NMR (150 MHz, MeOH-d_4) spectrum of compound **16**.

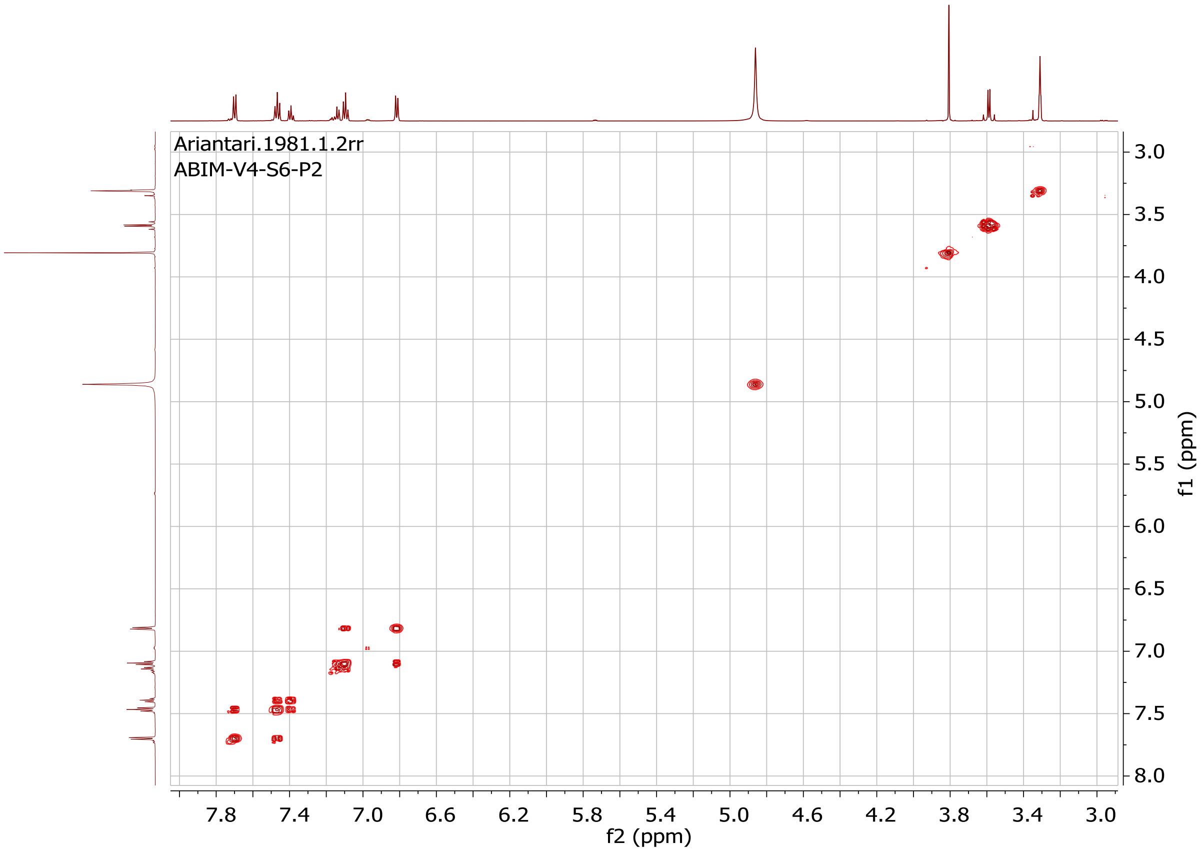

Figure S101. ^{1}H-^{1}H COSY (600 MHz, MeOH-d_4) spectrum of compound **16**.

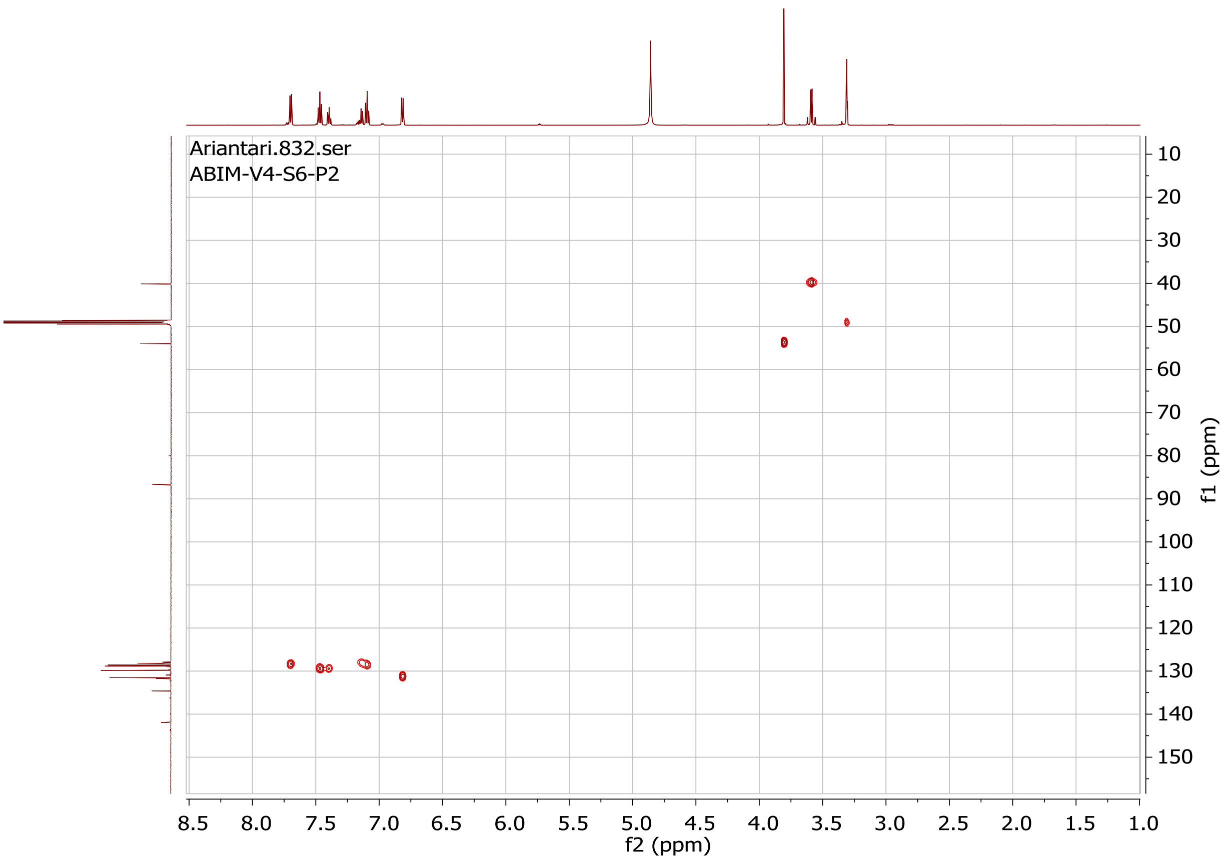

Figure S102. HSQC (600 and 150 MHz, MeOH-d_4) spectrum of compound **16**.

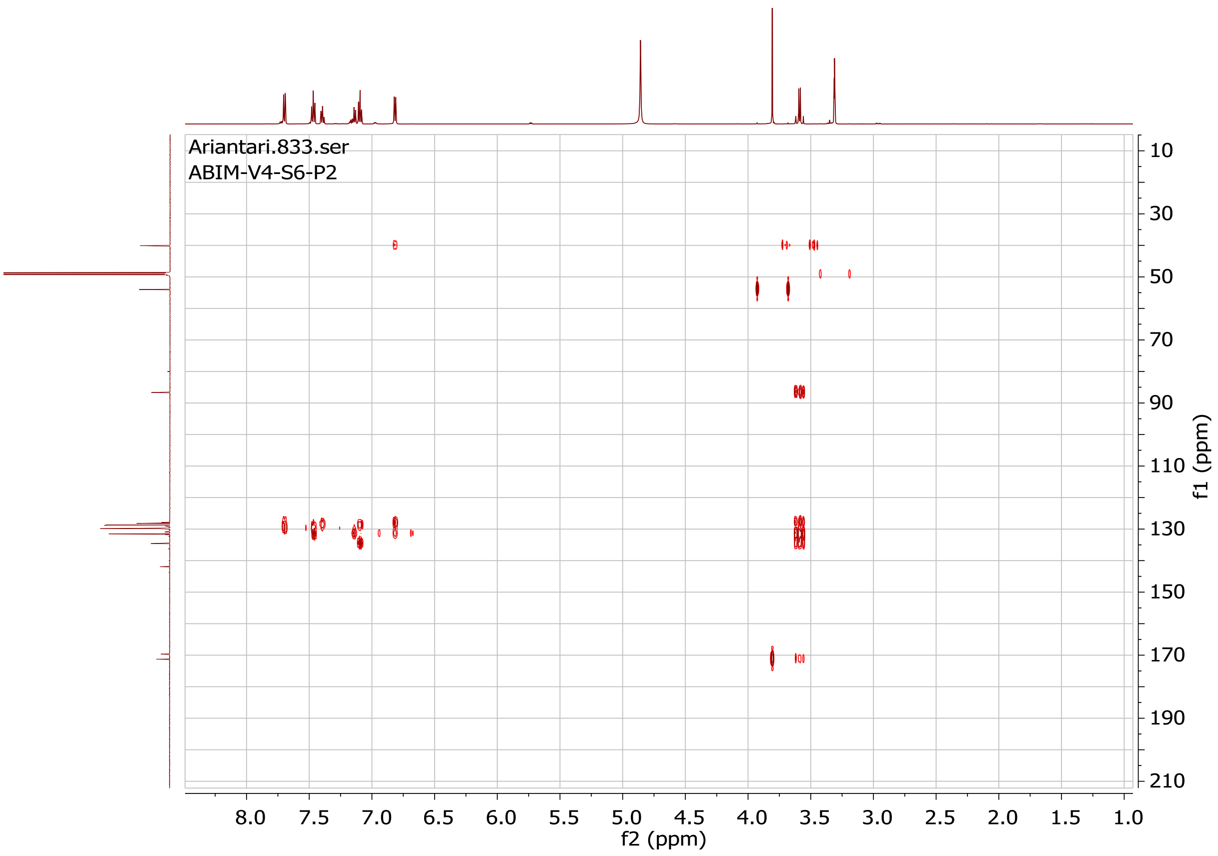

Figure S103. HMBC (600 and 150 MHz, MeOH-d_4) spectrum of compound **16**.

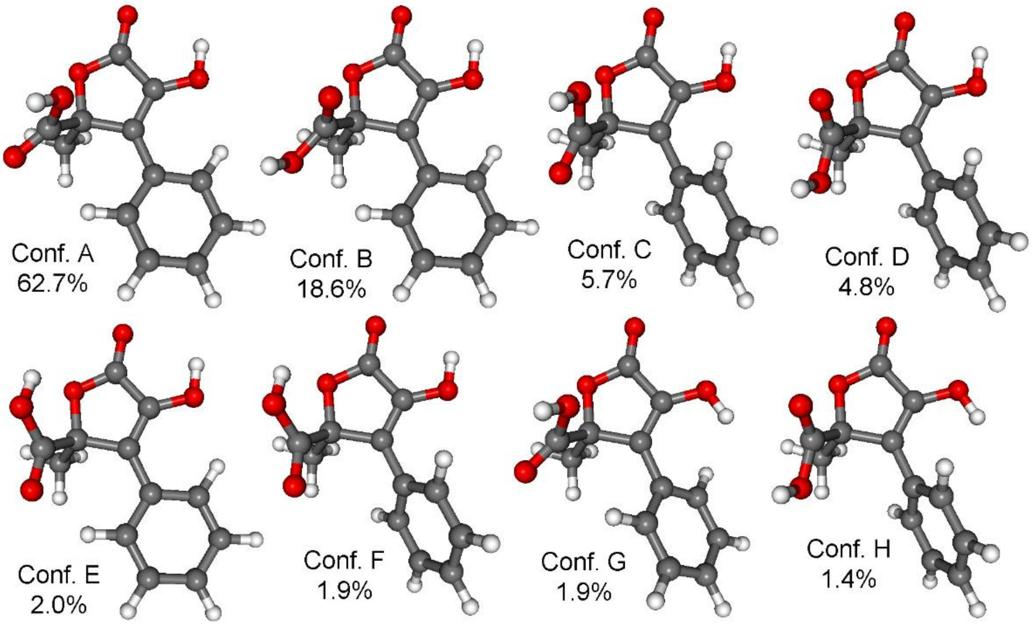

Figure S104. Structure and population of the low-energy CAM-B3LYP/TZVP PCM/MeCN conformers (≥ 1%) of (*S*)-**1**.

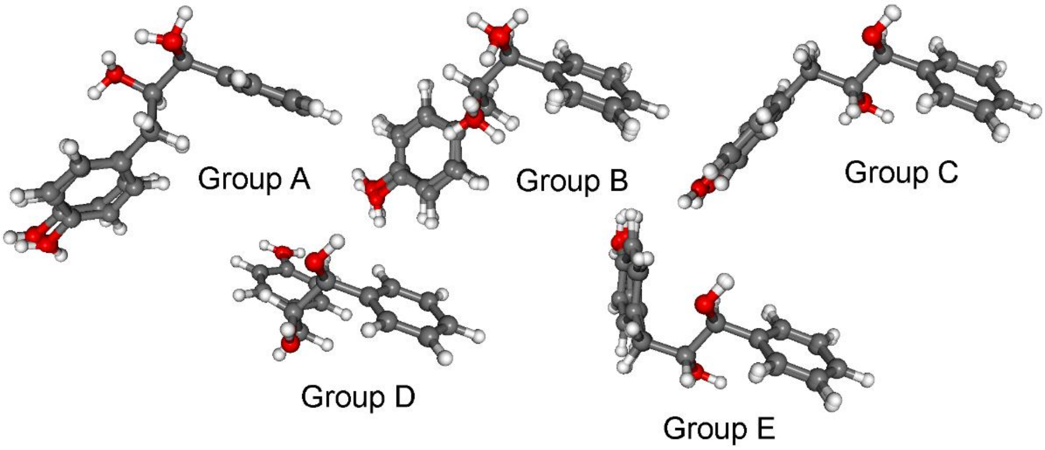

Figure S105. Classification of the twenty-two low-energy (≥ 1%) CAM-B3LYP/TZVP PCM/MeCN conformers of (8*R*,9*S*)-**4**. Group A (44.9%) contains conformers A, B, E, F; group B (27.5%) contains conformers C, D, G, H, K, L, Q, R; group C (10.1%) contains conformers I, J, M, N; group D (3.2%) contains conformers O, P; group E (4.4%) contains conformers S, T, U and V.

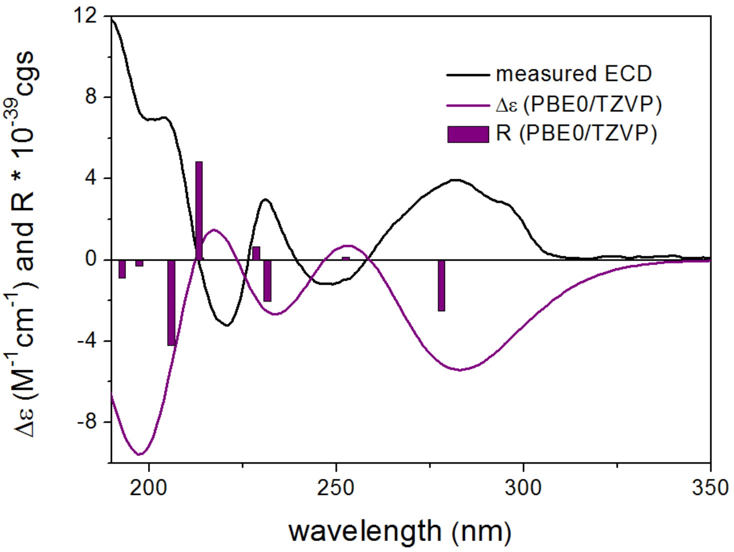

Figure S106. Experimental ECD spectrum (black) of **6** in MeCN compared with the Boltzmann-weighted PBE0/TZVP PCM/MeCN ECD spectrum (purple) of (*R*)-**6** computed for the 6 low-energy CAM-B3LYP/TZVP PCM/MeCN conformers. The bars represent the rotational strength of the lowest-energy conformer.

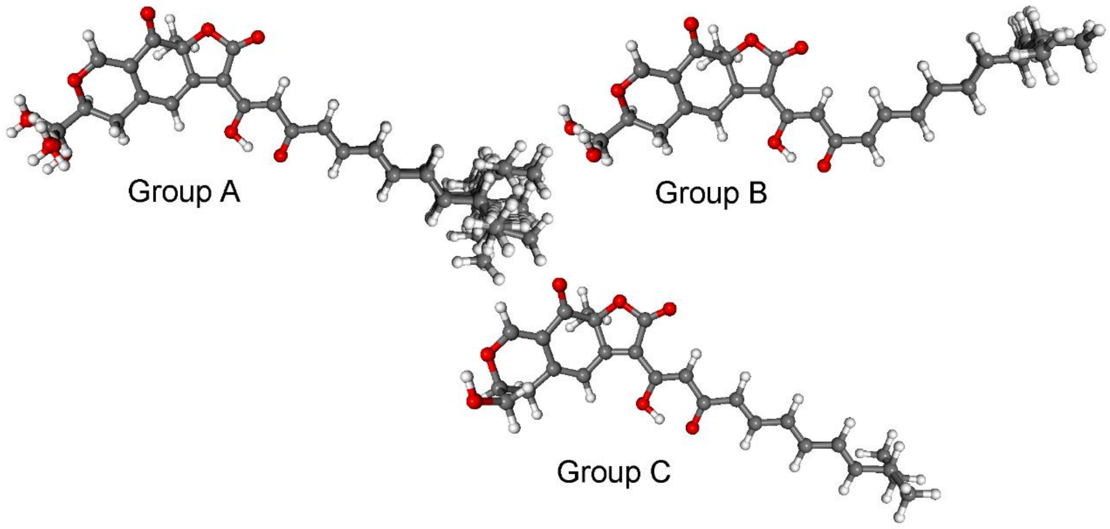

Figure S107. Classification of the twenty-six low-energy (≥ 1%) CAM-B3LYP/TZVP PCM/MeCN conformers of (3*S*,11*S*,23*S*)-**14** into conformer groups. Group A (72.5%) contains conformers A, B, C, D, E, F, G, I, J, K, M, N, O, Q, R, S, T, U, V, W, Y, Z; group B (5.0%) contains conformers H, L, X; group C (1.6%) contains conformer P.
